# Low tumour PPM1H indicates poor prognosis in colorectal cancer via activation of cancer-associated fibroblasts

**DOI:** 10.1038/s41416-019-0450-5

**Published:** 2019-04-16

**Authors:** Xiaowen Xu, Li Zhu, Yun Yang, Yamin Pan, Zhuo Feng, Ye Li, Wenjun Chang, Jinke Sui, Fuao Cao

**Affiliations:** 1 Department of Digestive Endoscopy, Shuguang Hospital, Shanghai University of Traditional Chinese Medicine, 528 Zhangheng Road, Shanghai, 201203 China; 2 Department of Colorectal Surgery, Changhai Hospital, Second Military Medical University, 168 Changhai Rd., Shanghai, 200433 China; 3 Department of Dermatology, First Affiliated Hospital, Wenzhou Medical University, Zhejiang, 325000 China; 4 The Third Department of Hepatic Surgery, Eastern Hepatobiliary Surgery Hospital, 225 Changhai Rd., Shanghai, 200438 China; 5 Department of Environmental Hygiene, Second Military Medical University, 800 Xiangyin Road, Shanghai, 200433 China

**Keywords:** Tumour biomarkers, Cancer microenvironment

## Abstract

**Background:**

Vimentin (VIM) is considered a prognostic marker in colorectal cancer (CRC). Our aim is to identify genes that fulfil a “X-low implies VIM-high” Boolean relationship and to evaluate their prognostic value and potential mechanism.

**Methods:**

Potential biomarkers related to VIM expression were searched using a bioinformatics approach across gene-expression arrays. Based on subgroup analysis of 2 CRC cohorts, the selected gene was tested for its association with patient’s survival outcomes. The regulatory link between the selected gene and VIM was further examined with in vitro models.

**Results:**

PPM1H was identified as the top candidate in our search. Patients with PPM1H-low tumours have a lower 5-year disease-free survival rate than patients with PPM1H-high tumours in 2 independent cohorts. In multivariate Cox analysis, patients with PPM1H-low tumours were independently associated with relapse in both the discovery cohort (hazard ratio [HR], 1.362; 95% confidence interval [CI], 1.015–1.826; *P* = 0.039) and the validation cohort (HR for DFS, 4.052; 95% CI, 2.634–6.234; *P* < 0.001). PPM1H knockdown in CRC cells and growth in the corresponding conditional medium increased VIM expression and colon fibroblast proliferation, indicating a transformation of cancer-association fibroblasts (CAFs). Conversely, educated CAFs also facilitated the growth of CRC cells with low PPM1H expression.

**Conclusions:**

Lack of tumour PPM1H expression identifies a patient subgroup with a high relapse risk, and CRC cells with low expression of PPM1H activate CAFs and inversely get promoted by CAFs.

## Background

Colorectal cancer (CRC) is one of the most common and lethal malignancies worldwide, accounting for approximately 1 in 10 cancer cases and deaths.^1^ Patients with localised and regional CRC generally receive surgical resection.^2^ However, a subset of these patients will relapse or develop metachronous metastases, which often lead to high mortality.^3^ Microarray-based gene-expression profiling has identified several prognostic signatures^3,4^ and molecular subtypes^5–8^ in CRC, and many of these profiles consistently emphasises that expanded mesenchymal components surrounding tumour cells, especially cancer-associated fibroblasts (CAFs), may importantly contribute to patient prognosis and drug resistance.^9–12^ CAFs are a heterogeneous and plastic population characterised by enhanced secretory phenotypes, robust autocrine activation and dynamic immunomodulatory functions that mainly originate from resident fibroblasts in tumours.^13^ The conversion of quiescent fibroblasts to CAFs is accompanied by unregulated molecule markers, such as αSMA, vimentin (VIM) and FAP.^13–15^ The interaction between CAFs and tumour cells is quite complicated. On one hand, CAFs facilitate the aggressive phenotype of tumour cells; on the other hand, tumour cells also prompt fibroblasts to transform into CAFs.^13^ Thus, CAFs represent a promising therapeutic target for tumour treatment.^16^ Currently, the process by which tumour cells educate the microenvironment is poorly understood in CRC.

**Fig. 1:**
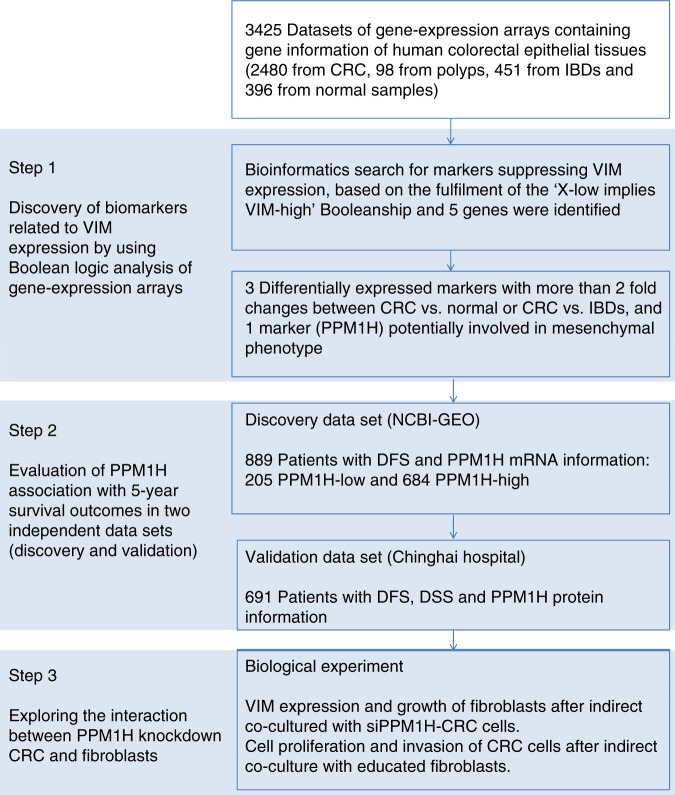
Outline diagram of this study. CRC colorectal cancer, IBD inflammatory bowel disease, DFS disease-free survival, DSS disease-specific survival

VIM is nearly always expressed in mesenchymal tissues and has been reported to have good performance for prognostic prediction of CRC.^17,18^ Therefore, we initiated a systematic search for markers that can suppress the expression of mesenchymal VIM, which may be able to suppress the activation of CAFs in CRC. The present study aimed to screen candidates that had a Boolean relationship with VIM in CRC using a bioinformatics approach^19–21^ (PPM1H was identified as the top candidate molecule) and to systematically evaluate the association between PPM1H expression and survival outcomes among CRC patients. In addition, we constructed in vitro cell models to verify the interactions between CRC cells with different levels of PPM1H expression and CAFs. The outline of this study is presented in Fig. 1.

## Materials and Methods

### Bioinformatics analysis

In total, 3425 colorectal gene expression array data sets (Supplementary Table 1) were collected and downloaded from the National Center for Biotechnology Information (NCBI) Gene Expression Omnibus (GEO) repository (www.ncbi.nlm.nih.gov/geo), containing gene expression information of colorectal tissues from 2480 CRCs, 98 polyps, 451 inflammatory bowel diseases (IBDs), and 396 normal mucosa tissues. The StepMiner approach^19^ was employed to identify the cutoff point for each gene across all the collected samples, which classified the samples into high or low expression subgroups. A bioinformatics search for candidate genes based on the fulfilment of a “X-low implies VIM-high” Boolean relationship (Supplementary Fig. 1) was implemented with 2480 human CRC gene-expression arrays using the BooleanNet software.^19–21^ The relationship between the expression of the candidates and some molecular features that are frequently observed in CRC (MSI and mutations of KRAS, BRAF, and TP53) were also explored based on the collection of available information. The details of the array collection and the bioinformatics analyses are documented in the online Data Supplement.

### Patients

Seven microarray data sets (GSE39582, GSE14333, GSE17538, GSE33113, GSE37892, GSE31595, and GSE39084) annotated with disease-free survival (DFS) information of stage I-III CRC patients were employed in this study. The baseline information of the discovery data set is documented in Table 1. The combined NCBI-GEO cohort was used as the “discovery data set”. Formalin-fixed, paraffin-embedded (FFPE) tissue specimens from 765 patients with localised CRC, which served as the validation cohort, were collected and used to construct tissue microarrays (TMAs) via a commercial company (Outdo Biotech, Shanghai, China). The details of TMA construction are described in the online Data Supplement. All specimen donors received curative surgery in Changhai Hospital, Second Military Medical University (Shanghai, China) between January 2008 and October 2011. The flow diagram and selection criteria of 691 study patients with stage I-III CRC are presented in Supplementary Fig. 2. The baseline information of patients is provided in Table 1. Less than 5% of patients with rectal cancer received preoperative radiotherapy in the cohort. DFS and disease-specific survival (DSS) information was followed-up at an interval of 6 months by 2 investigators (J. Sui and F. Cao). This work was approved by the Institutional Review Board of Changhai Hospital. Written informed consent was obtained from each patient.

**Table 1:** Characteristics of patient with CRC dichotomised by PPM1H expression in the discovery and validation data sets

Characteristics	Discovery data set (*n* = 889)			Validation data set (*n* = 691)		
	PPM1H-low (*n* = 205)	PPM1H-high (*n* = 684)	*P* value^a^	PPM1H-low (*n* = 229)	PPM1H-high (*n* = 462)	*P* value^a^
*Age (years), mean (SD)*	68.37 (12.91)	67.08 (13.11)	0.215^b^	60.42 (12.64)	60.92 (12.16)	0.615^b^
*Sex (n (%))*						
Women	102 (49.8)	312 (45.6)	0.297	108 (47.2)	193 (41.8)	0.179
Men	103 (50.2)	372 (54.4)		121 (52.8)	269 (58.2)	
*Disease location (n (%))*						
Colon	—	—		111 (48.5)	248 (53.7)	0.197
Rectum	—	—		118 (51.5)	214 (46.3)	
*Differentiation grade (n (%))*						
Well	—	—		6 (2.6)	9 (2.0)	0.139^c^
Moderately	—	—		150 (65.5)	334 (72.3)	
Poorly	—	—		73 (31.9)	119 (25.7)	
*Number of lymph nodes resected at surgery (n (%))*						
<12	—	—		47 (20.5)	103 (22.3)	0.595
≥12	—	—		182 (79.5)	359 (77.7)	
*TNM stage (n (%))*						
I	4 (2.0)	78 (11.4)	0.006^c^	38 (16.6)	93 (20.1)	0.046^c^
II	116 (56.6)	362 (52.9)		102 (44.5)	225 (48.7)	
III	85 (41.4)	244 (35.7)		89 (38.9)	144 (31.2)	
*Adjuvant chemotherapy (n (%))*						
Yes	80 (39.0)	215 (31.4)	0.001	161 (70.3)	291 (63.0)	0.055
No	77 (37.6)	369 (54.0)		51 (22.3)	133 (28.8)	
Missing	48 (23.4)	100 (14.6)		17 (7.4)	38 (8.2)	
*Serum CEA (ng/mL), median (range)*	—	—		3.91 (0.52–707.0)	3.25 (0–185.2)	0.011^c^
*Serum CA19–9 (U/mL), median (range)*	—	—		15.09 (0.6–4060.0)	11.5 (0–1200.0)	0.007^c^

^a^*χ*^2^ test or Fisher’s exact test

^b^Student *t* test

^c^Mann–Whitney *U* test (non-parametric). Missing values are excluded for all statistic tests

*CA19–9* carbohydrate antigen 19–9, *CEA* carcinoembryonic antigen, *TNM* tumour-node-metastasis

### Immunohistochemistry

The details of immunohistochemistry examination, scoring and analysis are described in the online Data Supplement.

### Survival analysis

Survival analyses of the subgroups in the discovery and validation data sets were performed using Kaplan–Meier curves and log-rank tests. A multivariate Cox proportional hazards model was used to evaluate the prognosis power of the gene-expression classification with other available factors (age, sex, tumour location, TNM stage, grade, chemotherapy, resected lymph nodes, serum CEA and CA199) as covariates. The association between PPM1H expression and survival outcome was analysed by an investigator (Z. Feng) who did not participate in the scoring process.

### Cell culture, RNA interference, quantitative RT-PCR, western blot, cell proliferation assay and invasion assay

The details are described in the online Data Supplement.

### Indirect cell co-culture

Briefly, siPPM1H-CRC and siControl-CRC cells were obtained 48 h after siRNA duplex transfection. The original medium was replaced with serum-free DMEM for an additional 24 h, and the corresponding supernatants were sterile filtered and mixed with FBS at a final concentration of 10% to generate the CRC conditional medium. Conditional medium from siPPM1H-CRC or siControl-CRC was used to culture CCD-18Co cells. After 5 days of culture, CCD18-Co cells in different culture media were harvested to examine the expression of VIM by qPCR and Western blotting. Previous studies reported that once normal fibroblasts are activated, they maintain their features in vitro. We expanded the educated CCD18-Co cells by culturing them in medium from siPPM1H-CRC cells or siControl-CRC cells and named them activated-CCD18-Co and CCD18-Co-control, respectively. Then, the conditional medium was collected to culture CRC cells as described above. CRC cell proliferation and invasion in response to conditional media from activated-CCD18-Co and CCD18-Co-control were further evaluated.

### Statistical analysis

Patient subgroups stratified by gene or protein expression were compared for survival outcomes using both Kaplan–Meier curves and a multivariate Cox proportional hazards model. Differences in the Kaplan–Meier curves were assayed with the log-rank test to assess significance. The interactions between PPM1H status and adjuvant chemotherapy were examined using the Cox model with a 2 × 2 factorial design, which explored the presence of a multiplicative or additive effect between the hazards rates caused by each of the two variables individually. Cell proliferation, invasiveness or gene expression levels between the different treatments were tested with independent samples Student *t* test. All statistics were two-sided and conducted using R (http://www.r-project.org/) and SPSS V.16.0.2 for Windows (SPSS, Chicago, Illinois, USA). Significance was set as P<0.05.

## Results

### PPM1H identified by bioinformatics analysis

The search for genes that fulfil a “X-low implies VIM-high” Boolean relationship revealed 95 genes with a false-discovery rate (FDR) < 0.005. Of these genes, 90 genes were identified based on samples from less than 40% of the 2480 cancer arrays. Thus, we selected another 5 genes (PCGF1, NFE2L3, GTF2IRD1, PPM1H, and CIRH1A) for further evaluation (Supplementary Table 2). Three genes (NFE2L3, GTF2IRD1, and PPM1H) were significantly differentially expressed between cancer and normal cells or between cancer and IBDs with greater than 2-fold changes (all *P* < 0.001) (Supplementary Fig. 3). GTF2IRD1 is associated with higher-grade tumours and poor prognosis and has been identified as a tumour promoting gene in breast cancer.^22^ NFE2L3 is a basic-region leucine zipper transcription factor and has a protective effect against lymphomagenesis induced by benzo[a]pyrene (B[a]P), suggesting that it plays a role in carcinogenesis of haematopoietic malignancies.^23^ Interestingly, PPM1H suppresses epithelial-mesenchymal transition (EMT)^24^ and mesenchymal differentiation,^25^ consistent with the finding that knockdown of PPM1H elevates VIM expression in pancreatic cancer.^24^ Moreover, PPM1H can sensitize cells to trastuzumab for HER2-targeted therapy.^26^ Based on these findings, we selected PPM1H as the candidate gene for further evaluation.

The combined gene expression pattern of PPM1H and VIM indicates the existence of three subgroups: PPM1H-low and VIM-high, PPM1H-high and VIM-high, and PPM1H-high and VIM-low (Supplementary Fig. 4). Patients with PPM1H-low expression were restricted to a small subgroup in the CRC gene-expression array data set (22.6%, 561/2480) (Supplementary Fig. 4). This subgroup was characterised by high levels of VIM expression and significantly overlapped with the patient subset with BRAF mutations (Supplementary Fig. 5A) and the subset with MSI (Supplementary Fig. 5B).

### Low PPM1H expression predicts unfavourable survival in the NCBI-GEO discovery data set

**Fig. 2:**
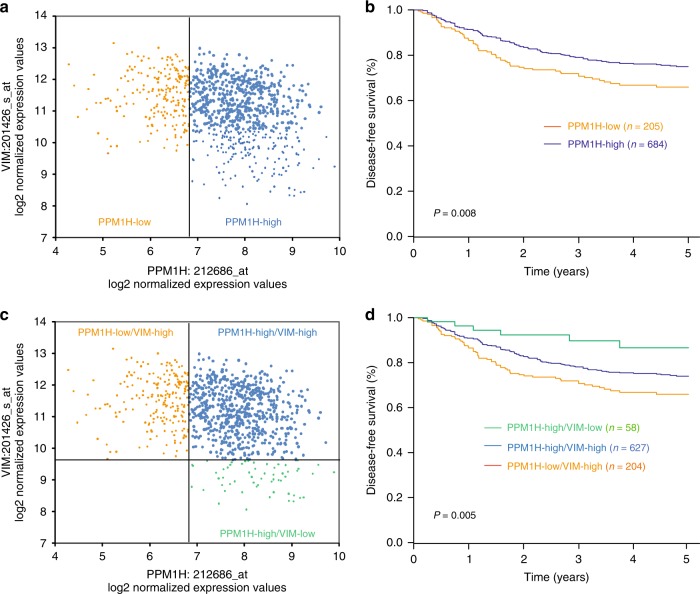
Relationship between the patient subgroups classified by mRNA expression of PPM1H and VIM and disease-free survival in the discovery data set. (**a**) Classification of the subgroups with high or low PPM1H mRNA expression; (**b**) survival analysis between patients with PPM1H-high tumours or PPM1H-low tumours; (**c**) three patient subgroups defined by PPM1H and VIM mRNA expression; (**d**) survival analysis among the subgroups defined by the PPM1H/VIM system. The location of the cut lines was defined according to StepMiner analysis

We classified the 889 patients with stage I-III CRC into PPM1H-low or PPM1H-high subgroups in the NCBI-GEO discovery data set (Fig. 2a) and then evaluated the association between PPM1H expression and DFS. The result showed that the 5-year DFS rate was lower among patients with PPM1H-negative tumours than patients with PPM1H-high tumours (65.9% vs. 75.0%, *P* = 0.008) (Fig. 2b). Upon multivariate analysis, patients with PPM1H-low tumour had a higher risk of CRC relapse compared with patients with PPM1H-high tumours (HR, 1.370; 95% CI, 1.032 to 1.818; *P* = 0.029) (Table 2).

**Table 2:** Cox regression analysis of immunohistochemistry PPM1H expression and clinicopathological covariates in the discovery and validation data sets

Characteristics	Disease-free survival				Disease-specific survival			
	Univariate		Multivariate		Univariate		Multivariate	
	HR (95% CI)	*P* value	HR (95% CI)	*P* value	HR (95% CI)	*P* value	HR (95% CI)	*P* value
Discovery data set (*n* = 889)								
PPM1H-low vs. PPM1H-high	1.520 (1.177–1.963)	0.001	1.370 (1.032–1.818)	0.029	—	—	—	—
Age ( > = 60 vs*.* *<* 60)	1.001 (0.990–1.011)	0.910	1.181 (0.848–1.645)	0.326	—	—	—	—
Sex (male vs. female)	1.163 (0.887–1.525)	0.275	1.230 (0.915–1.653)	0.169	—	—	—	—
Tumour stage, per increase in stage	2.315 (1.819–2.945)	<0.001	2.266 (1.656–3.101)	<0.001	—	—	—	—
Chemo (yes vs. no)	0.574 (0.429–0.769)	<0.001	0.936 (0.655–1.338)	0.718	—	—	—	—
*Validation data set* (*n* = 691)								
PPM1H-low vs. PPM1H-high	3.878 (2.664–5.643)	0.000	4.052 (2.634–6.234)	0.000	2.525 (1.379–4.625)	0.002	2.608 (1.327–5.128)	0.005
Age ( > = 60 vs. < 60)	1.016 (0.705–1.466)	0.930	1.329 (0.867–2.037)	0.192	1.183 (0.639–2.191)	0.592	1.077 (0.541–2.144)	0.833
Sex (male vs. female)	1.088 (0.753–1.573)	0.652	1.117 (0.734–1.699)	0.606	1.197 (0.646–2.216)	0.568	1.123 (0.565–2.229)	0.741
Location (colon vs. rectal)	0.929 (0.645–1.340)	0.695	0.866 (0.571–1.315)	0.500	1.331 (0.723–2.451)	0.357	1.360 (0.685–2.699)	0.379
TNM, per increase in stage	1.818 (1.373–2.406)	<0.001	1.496 (1.027–2.179)	0.036	1.069 (0.686–1.665)	0.769	1.392 (0.691–2.802)	0.355
Grade (poorly vs.others)	2.039 (1.427–2.915)	<0.001	1.626 (1.072–2.468)	0.022	1.670 (0.913–3.054)	0.094	1.329 (0.670–2.635)	0.416
Chemo (yes vs. no)	0.721 (0.443–1.172)	0.185	0.799 (0.426–1.500)	0.485	0.948 (0.454–1.978)	0.887	0.897 (0.311–2.588)	0.840
Resected lymph node ( ≥ 12 vs*.* *<* 12)	3.747 (1.951–7.196)	<0.001	3.544 (1.815–6.918)	< 0.001	2.977 (1.236–7.169)	0.011	2.756 (1.110–6.844)	0.029
Serum CEA (ng/ml)	1.558 (1.082–2.243)	0.016	1.419 (0.928–2.170)	0.107	1.627 (0.888–2.981)	0.112	2.018 (1.011–4.029)	0.047
Serum CA199 (U/ml)	1.992 (1.287–3.082)	0.002	1.128 (0.653–1.949)	0.666	1.209 (0.509–2.870)	0.667	1.400 (0.473–4.149)	0.543

*HR* hazard ratio, *CI* confidence interval

High VIM expression is associated with poor prognosis in CRC.^14,15^ When VIM is defined as a continuous variable, we found that patients with VIM-high tumours had worse outcomes compared with patients with VIM-low tumours (HR, 1.451; 95% CI, 1.230–1.713; *P* < 0.001) in the NCBI-GEO data set. Next, we evaluated whether the association between low PPM1H expression and a low DFS rate could be explained by the finding that most patients with low PPM1H expression simultaneously exhibited high VIM expression. To this end, we stratified the discovery population into three subgroups (PPM1H^low^/VIM^high^, PPM1H^high^/VIM^high^, and PPM1H^high^/VIM^low^) and then compared their clinical outcomes (Fig. 2c). The results showed that patients with PPM1H^low^/VIM^high^ had the lowest rate of 5-year DFS among the 3 subgroups and that the group of PPM1H^high^/VIM^high^ exhibited intermediate DFS, as expected (Fig. 2d). Moreover, multivariate Cox analysis showed that the PPM1H/VIM grouping system is an independent risk factor for CRC relapse when age, sex, and the TNM stage were considered as confounding variables (Supplementary Table 3).

### Validation of the prognostic role of PPM1H in the Changhai validation set

**Fig. 3:**
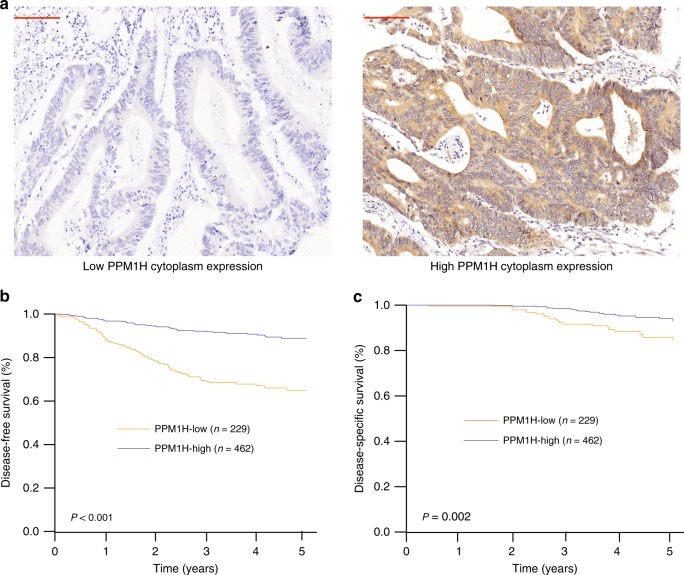
Relationship between the patient subgroups classified by PPM1H protein expression and survival outcomes in the validation data set. (**a**) Represented protein expression of cytoplasmic PPM1H; (**b**) DFS analysis between patient subgroups with high or low PPM1H protein expression; (**c**) DSS analysis between patient subgroups with high or low PPM1H protein expression. The red bar in the figure represents 100 μM. DFS, disease-free survival; DSS, disease-specific survival

The findings described above were further tested to assess their robustness. The CRC TMAs of the Changhai validation cohort were examined by IHC. PPM1H staining was mainly distributed in the cytoplasm (Fig. 3a) and partly observed in the nucleus of colorectal epithelial cells. Nuclear staining is not used in this study due to specificity (Supplementary Fig. 6). Based on the staining scores of all sections, we confirmed the presence of 2 different subgroups with low (33.1% [229/691]) or high (66.9% [462/691]) cytoplasmic PPM1H staining in malignant epithelial cells (Supplementary Fig. 7). Survival analysis showed that PPM1H-low tumours were associated with worse prognosis compared with PPM1H-high tumours, with 5-year DFS of 64.9% vs. 88.8% (P<0.001) and 5-year DSS of 84.4% vs. 92.9% (*P* = 0.002) (Fig. 3b, c). The association remained significant in multivariate analysis. Patients with PPM1H-low tumours were at higher risk of recurrence based on DFS (HR, 4.052; 95% CI, 2.634 to 6.234; P<0.001) and higher risk of death based on DSS (HR, 2.608; 95% CI, 1.327–5.128; *P* = 0.005) (Table 2).

### PPM1H expression predicts the prognosis of patients with early stage CRC

The association between PPM1H expression and DFS among patients with earlier stage CRC (I and II stage) was further explored. Low PPM1H expression was associated with lower 5-year DFS compared with high PPM1H expression in both the discovery (68.3% vs. 83.9%, *P* < 0.001) and validation data sets (71.4% vs. 91.8%, *P* < 0.001) (Supplementary Fig. 8A). Similar results for 5-year DSS were also found in the validation cohort (84.5% vs. 93.2%, *P* = 0.013) (Supplementary Fig. 8A). For stage II disease alone, patients with PPM1H-low tumours had lower DFS in both the discovery data set (*P* = 0.002) and validation data set (*P* < 0.001) (Supplementary Fig. 8B).

### PPM1H expression and benefit from adjuvant chemotherapy

Next, we investigated the relationship between PPM1H expression status and survival outcomes among patients who did or did not receive chemotherapy (Supplementary Fig. 9). For stage II CRC, the results showed no obvious correlation between PPM1H expression status and the benefit from adjuvant chemotherapy in both the discovery and validation cohorts. For stage III CRC, patients with PPM1H-high tumours tended to receive benefits from chemotherapy exclusively in the validation cohort. Moreover, the multiplicative or additive effects between PPM1H status and chemotherapy treatment were also not found in stage II or stage III CRC (all *P* > 0.05).

### PPM1H-low CRC cells promote CAF activation

**Fig. 4:**
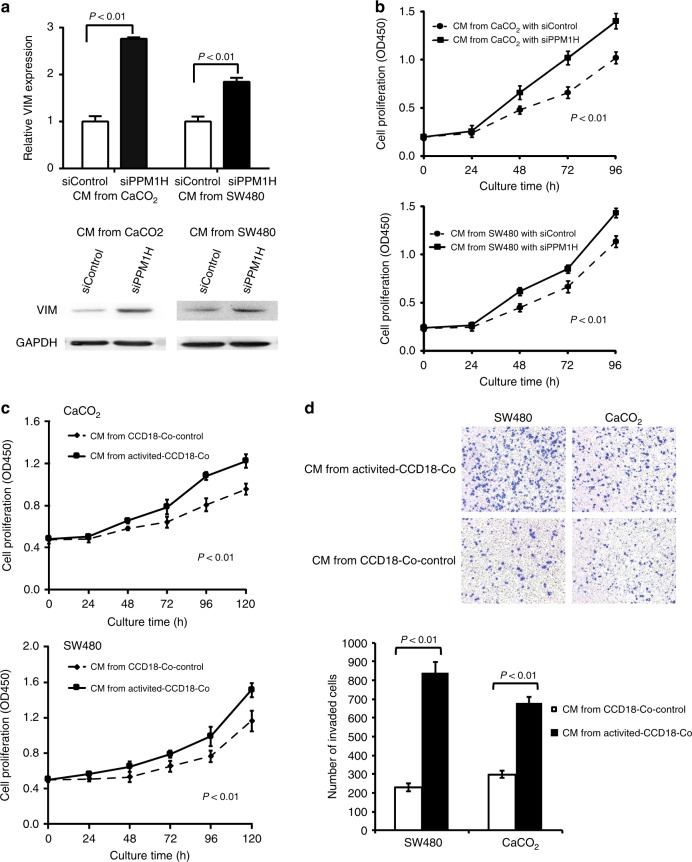
Knockdown of PPM1H in CRC cells increased VIM expression in fibroblasts and its proliferation, and activated fibroblasts facilitated CRC cell proliferation and invasion. (**a**) VIM mRNA or protein expression of CCD-18Co with respect to the different CMs from the indicated CRC cells after siPPM1H or siControl interference; (**b**) proliferation of CCD-18Co with respect to the different CMs from the indicated CRC cells after siPPM1H or siControl interference; (**c**) proliferation of SW480 and CaCO2 cultured with the different CMs from activated CCD-18Co and CCD-18Co-control; (**d**) cell invasion of SW480 and CaCO2 cultured with different CMs from activated CCD-18Co and CCD-18Co-control. CMs, conditional media. Each assay was repeated thrice

VIM is mainly expressed in the mesenchymal cells of CRC^13,14^ and frequently serves as a marker of CAFs. Activated resident fibroblastic cells are the largest source of CAFs.^13^ Therefore, the effects of tumour PPM1H on VIM expression in fibroblasts and on the activation of CAFs were further assessed with a co-culture system in vitro. The CRC cell lines CaCO2 and SW480, in which PPM1H expression is at a high level, were selected (Supplementary Fig. 10A). PPM1H was knocked down in CRC cell lines; thus, siPPM1H-CRC cell lines were established (Supplementary Fig. 10B). When cultured with the conditional medium from siPPM1H-CRC cells, colonic fibroblast cells CCD18-Co showed significantly elevated expression of VIM (Fig. 4a) and increased proliferation (Fig. 4b) compared with cells cultured in the conditional medium from siControl-CRC cells. The results indicated that PPM1H-low CRC cells might play a role in promoting the transition of CCD18-Co cells into activated CAFs. Then, the effects of activated CCD18-Co cells on the phenotype of CRC cells were evaluated. Interestingly, when CRC cells were co-cultured with medium from activated CCD18-Co cells, the proliferation and invasiveness of CRC cells were enhanced (Fig. 4c, d), indicating that CCD-18Co cells educated by PPM1H-low CRC cells secret some cytokines to promote cancer aggressiveness. Therefore, our results clearly demonstrate that PPM1H-low tumour cells may activate CAFs and are subsequently supported by CAFs.

## Discussion

The CRC subtype with enriched mesenchymal components is associated with poor prognosis and chemo-resistance.^5–12,14,15^ As an important mesenchymal marker, VIM is popularly used in various types of cancers,^5–14,16^ including CRC. In this study, we proposed that there are some markers for which low expression implied high expression of VIM based on a Boolean implication analysis, and these markers may be associated with the prognosis of CRC patients. Based on a bioinformatics analysis and gene background screening, we identified PPM1H as the top candidate gene for further study. PPM1H suppresses the activation of SMAD signalling and participates in the process of mesenchymal differentiation.^25^ In particular, knockdown of PPM1H in pancreatic cancer cells results in increased VIM expression and changes in other EMT markers,^24–26^ suggesting an obvious role of PPM1H in the mesenchymal phenotype. Our study results also demonstrated that Boolean logic analysis is an effective method for biomarker searches.

On the basis of the collected microarrays, we found that the subgroup with low PPM1H expression was characterised by high levels of VIM expression and that this group significantly overlapped with the subset with MSI or BRAF mutations, indicating that PPM1H is potentially involved in the aggressiveness of CRC. Using the NCBI-GEO discovery data set, we found that patients with low PPM1H expression tumours typically had shorter DFS compared with those with high PPM1H expression tumours. In multivariate Cox analysis, low PPM1H expression was an independent risk factor for CRC prognosis; however, several confounding factors existed. Expression PPM1H in the discovery data set was confined to the mRNA level. However, proteins are more relevant to biological function. Based on an IHC examination of the validation data set, we confirmed that patients with tumours that expressed low PPM1H at the protein level still had shorter DFS and DSS. Similar results were also obtained in multivariate Cox model analysis independent of the covariates.

For many types of cancers, tumour stage has been widely proven to be an important prognosis factor. We assayed the prognostic relevance with respect to the PPM1H status in early stage (stage I–II) CRC patients. Different PPM1H expression statuses can discriminate the survival outcomes of CRC patients consistently in both discovery and validation data sets, suggesting that PPM1H is a predictive marker for prognosis in early stage CRC. Some stage II CRC patients with risk factors and all stage III CRC patients are routinely assigned to receive chemotherapy regimens, so the association between PPM1H expression and the efficiency of chemotherapy was explored in this study. Our analyses showed that no significant benefit could be obtained from the chemotherapy regimens in patients with stage II disease. For stage III CRC, patients with tumours that expressed high PPM1H seemed to benefit more from chemotherapy in the validation data set. PPM1H knockdown induced the reduction of the tumour suppressor p27 at the protein level,^26^ and low p27 protein expression was associated with chemo-resistance, such as cisplatin and carboplatin resistance.^27^ Thus, we proposed that the benefits of chemotherapy for patients with low PPM1H expression might be offset by the reduction of p27 in stage III CRC.

Next, we began to explore the regulatory relationship between tumour PPM1H expression and mesenchymal VIM expression. VIM is mainly sourced from fibroblasts or fibroblast-like cells.^12–18^ Fibroblast-like cells may originate from the mesenchymal conversion of tumour cells,^12,13,16^ and the process is typically referred to as EMT. With PPM1H knockdown, we found that VIM expression in CaCO2 and SW480 cells increased at both the mRNA and protein levels (Supplementary Fig. 10C-D). Meanwhile, EMT markers of E-Cadherin (CDH1) and N-Cadherin (CDH2) in CRC cells were significantly down-regulated and up-regulated, respectively (Supplementary Fig. 10C-D). These results indicate that a lack of PPM1H could drive EMT of CRC cells. Then, we examined the change in VIM expression in normal colon fibroblasts when they were cultured in the conditional medium of PPM1H-knockdown CRC cells. The results consistently demonstrated that CRC cells with low PPM1H expression could elevate VIM expression in fibroblasts and promote cell proliferation, leading to CAF activation. As expected, fibroblasts activated by low PPM1H CRC cells significantly facilitated the growth and invasion of CRC cells. Therefore, our results clearly revealed that low PPM1H expression in CRC could lead to tumour development through activating CAFs.

This study had some limitations as follows. First, batch bias of microarray experiments may have been introduced when we combined the array experiments from GEO; however, single sample fRMA^28^ was used to extract the gene expression profiles. Second, StepMiner^19^ or other methods were available to classify the samples as high or low expression for each gene, but the identified candidates that fulfilled a “X-low implies VIM-high” relationship might have varied if a different method was used. Third, we cannot exclude the bias from the loss of follow-up in our data set owing to unappreciated factors. Finally, some factors, such as MSI and extramural venous invasion, were not included in our cohort given the limited specimen resources, which led to covariate incompetence in multivariate Cox analysis.

In summary, our study suggests that low PPM1H expression in cancer cells is associated with a poor outcome in CRC (stage I-III) and is a potentially useful IHC marker for the prognosis of CRC. The preliminary experimental data show that tumour PPM1H may affect EMT of CRC cells and activation of CAFs. Further studies are required to better understand PPM1H as a prognostic marker in CRC with a prospective cohort. More detailed mechanistic studies need to be performed to discover the paracrine signalling pathway of CRC cells induced by PPM1H.

## Supplementary information

Data Supplement

## Data Availability

All the data analysed or generated in this study are included in this article and its supplementary information file.
