# Supplementary material for: Low tumour PPM1H indicates poor prognosis in colorectal cancer via activation of cancer-associated fibroblasts
Source: Br J Cancer. 2019 Apr 16;120(10):987–95. doi: 10.1038/s41416-019-0450-5 (PMC6734651; doi:10.1038/s41416-019-0450-5)
Supplement: Supplementary file 1 — Data Supplement [file 41416_2019_450_MOESM1_ESM.pdf]

Data Supplement

Low tumor PPM1H indicates poor prognosis in colorectal cancer via activation of cancer-associated fibroblasts

Xiaowen Xu, Li Zhu, Yun Yang, Yamin Pan, Zhuo Feng, Ye Li, Wenjun Chang, Jinke Sui and Fuao Cao

Table of Contents:

Supplementary Methods
Supplementary Table 1
Supplementary Table 2
Supplementary Table 3
Supplementary Table 4
Supplementary Figure 1
Supplementary Figure 2
Supplementary Figure 3
Supplementary Figure 4
Supplementary Figure 5
Supplementary Figure 6
Supplementary Figure 7
Supplementary Figure 8
Supplementary Figure 9
Supplementary Figure 10
Supplementary Figure 11
Supplementary Figure 12

Supplementary Methods

Constructed gene-expression array databases to search for candidate biomarkers. We retrieved colorectal epithelial tissue-related microarray experiments conducted on an Affymetrix HG-U133 Plus 2.0 platform (GPL570) from the National Center for Biotechnology Information (NCBI) Gene Expression Omnibus (GEO) repository (www.ncbi.nlm.nih.gov/geo) using the key words (*GPL570 AND (colon OR rectum OR colorectal)*) released up to Dec 4, 2016. A total of 273 array series were first identified. After carefully reading the details, 70 data series were retained for further review. The excluded data series met any one of the followed criteria: 1) specimens without colorectal epithelial tissue; 2) only cell line samples (usually with duplicated mRNA profile data because the same cell lines were usually used in different microarray experiments) or cell lines derived from PDX tissues (usually mixed with mouse stroma component) are available; 3) CRC PPFE tissues; 4) microdissected tissues; 5) data set without raw data available; and 6) duplicated data series. The gene expression profiles of 70 array data series were individually extracted from the raw data with *fRMA* package[28] in the R 3.0.1 environment. Normalization was performed with the quantile algorithm after the whole array collection was pooled. Jetset software[29] was also used to select the optimal microarray probe to represent a gene. Pearson coefficients among array expression profiles were calculated. Triplicate or duplicate samples (pair-wised correlation coefficient = 1.0) were removed, and 3821 samples remained. To exclude samples with significant contamination from other tissues, such as normal liver tissue, we restricted our investigation using those samples to the expression pattern of EpCAM^{pos}/Almumin^{neg}. EpCAM and albumin (ALB) were positive markers for the presence of colorectal epithelial cells and hepatocytes, respectively. EpCAM^{pos} was defined as Affymetrix probe 201839_s_ >11.478 (97.5% quantile value), and albumin^{neg} was defined as Affymetrix probe 211298_s_ < 4.201 (2.5% quantile value), as shown in Supplementary Fig.12. This operation removed 396 arrays, and

3425 arrays remained for subsequent analysis (colorectal carcinoma: n =2,480; polyps: n=98; inflammatory bowel diseases (IBDs): 451; normal colorectal mucosa: n = 396). A complete list of all the GSMIDs of the samples contained within the final database is provided in Supplementary Table 1.

Defined gene expression thresholds to discriminate negative and positive samples. The StepMiner algorithm[19] was chosen to define the thresholds of the gene expression profile from the array collection, which could be used to classify the samples as low or high expression. Briefly, for each gene, we sorted the expression values of all 3425 samples from low to high and fitted the ordered data with an increasing step function. Next, we used the StepMiner algorithm to search for the step of the largest increasing jump and identified that point as the threshold for each gene. An intermediate region was defined around the threshold using a width of 1 (0.5 below and 0.5 above the threshold), corresponding to a 2-fold change in expression, which was the minimum noise level in the collected array experiments. Low expression samples were considered when their expression was less than the intermediate region (< StepMiner threshold-0.5). Inversely, samples greater than the intermediate region (> StepMiner threshold+0.5) were considered to be high expression samples. The details are shown in Supplementary Fig.1.

Mining the pooled gene expression profile using Boolean logic rules. Based on the thresholds identified by the *StepMiner* algorithm, any 2 genes may form a four-fold table with the intermediate regions (Supplementary Fig.1). Gene pairs between VIM and each (X) of the other genes were analysed to discover the expression implications of “X-low implies VIM-high”. The implication was detected by evaluating whether the lower-left quadrant in the scatter plot of Supplementary Fig.1 was significantly sparsely populated with sample points compared with the other quadrants (intermediate values for VIM and X were ignored in this

analysis). When the false-discovery rate (FDR) of a sparsity test was < 0.005 in the lower-left quadrant of a "X vs. VIM" two-axis plot and the points in all the quadrants were greater than 40% of the cancer samples, a Boolean implication was identified. A permutation test was used to evaluate the robustness of each FDR value. The search resulted in 5 genes (Supplementary Table 2), and we found that the expression pattern of 3 genes (PPM1H, GTF2IRD1, and NFE2L3) exhibited greater than 2-fold changes between CRC and normal tissues or between CRC and IBD tissues (Supplementary Fig.3). GTF2IRD1 has been identified as a tumour promoting gene.[22] NFE2L3 has a protective role against lymphomagenesis induced by benzo[a]pyrene (B[a]P).[23] PPM1H suppresses EMT phenotypes and is involved in mesenchymal differentiation.[24,25] Therefore, we selected PPM1H for further investigation.

Association between PPM1H expression and molecular features frequently investigated in CRC. Information regarding microsatellite instability (MSI) and gene mutations (KRAS, BRAF, TP53) for NCBI-GEO CRC samples was reviewed and further investigated. Eight array series (GSE26682, GSE13294, GSE13067, GSE35896, GSE18088, GSE20540, GSE39084, and GSE34489) were pooled, and 557 independent primary tumours were identified to test the relationship between PPM1H expression and MSI. In this analysis, all samples originally classified as MSI-low were re-classified as microsatellite stable (MSS). Three array series (GSE39084, GSE35896, and GSE39582) were pooled, and a total of 602 independent primary tumours were identified to test the relationship between PPM1H mRNA expression and KRAS, BRAF (n=568), or TP53 mutations. The enrichment in PPM1H-low CRC by the involved molecular features was tested using he Pearson's X^2 test and computed odds-ratios (OR) together with their 95% confidence intervals (CI). The differences in PPM1H expression levels among different patient subsets were evaluated using box plots and were tested using a 2-sample t-test (2-tailed) for statistics significance.

Construction of the discovery dataset and its stratification of patients. The association between gene expression and patient survival was explored in a subset of patients of our collection from GEO-NCBI in which each patient was supplemented with disease-free survival (DFS) information. The subset contained 889 patients with stage I-III CRC pooled from 7 gene expression data sets (GSE39582, GSE14333, GSE17538, GSE33113, GSE37892, GSE31595, GSE39084) and served as the "discovery dataset" in our study. In this data set, 741 patients were supplemented with information regarding adjuvant chemotherapy, and only 63 patients were annotated with pathological grade information.

To investigate the association between the expression status of the selected genes (i.e., PPM1H, VIM) and the survival outcomes of patients represented within the discovery dataset, we first classified the patients into different gene expression subgroups according to the cut-offs identified by the StepMiner algorithm based on the distribution of all colorectal tissue array experiments. $PPM1H^{low}$ tumours were defined as Affymetrix probe 212686_ < 6.835, while VIM^{low} tumours were defined as Affymetrix probe 201426_s_ < 9.629 (Fig. 2). Based on the definitions, the patients from the discovery dataset were stratified into different gene-expression subgroups depending on either expression status of PPM1H alone, VIM alone, or a combination of PPM1H and VIM. Once subgroups were identified, the survival outcomes were compared among the different subgroups using Kaplan-Meier curves and multivariate Cox model.

Construction of tissue microarrays (TMAs). Seven hundred sixty-five formalin-fixed, paraffin-embedded tissue blocks were processed to make tissue microarrays. Haematoxylin and eosin (HE)-stained slides from the FFPE specimens were reviewed by qualified pathologists who circled the interested region on the corresponding blocks for coring. A 1.5 mm diameter core in the circling region was released with a needle from each block and re-embedded in a recipient block. Each recipient block contained approximately

180 cores supplemented with an in-cohort control from the same 5 blocks. TMA cutting was performed by a skilled pathologist, and all resulting slides were dipped in paraffin for preservation at 4°C.

Immunohistochemical testing. Antibody specificity to the target gene is very important for the examination of IHC analysis. We first evaluated the robustness of the antibodies to PPM1H (HPA04424, Sigma-Aldrich, St Louis, MO, USA) with cell models. Briefly, we first treated SW480 cells with validated PPM1H-siRNA duplex or negative control siRNA for 48 hours and then collected the lysates for Western blotting. As shown in Supplementary Fig.6A, there were two main bands: the lower band with a small molecular weight was PPM1H, which was affected by si-PPM1H, and the upper band represented an unknown protein, which was unaffected by si-PPM1H. Moreover, we collected the lysates from the cytoplasm part and nuclear part separately with NE-PERTM Nuclear and Cytoplasmic Extraction Reagents (Ca#78833, Thermo) for Western blot analysis and confirmed that the upper band only existed in the nuclear portion (Supplementary Fig.6B). The results clearly demonstrated that the anti-PPM1H antibody (1:200, HPA04424, Sigma-Aldrich) was only specific for cytoplasmic PPM1H. Thus, we used the antibody for IHC examination with TMAs and only considered cytoplasm staining. The immune-staining protocol was based on the manufacturer's recommendation. Notably, PPM1H antigens were retrieved with citrate buffer (pH 6.0).

Analysis of tissue microarrays. The clinical and pathological information for TMAs was obtained from Changhai Hospital. All tumours in TMAs were scored blindly by one of the authors. A detailed description of the scoring system together with representative photographs and the scoring results are provided in Supplementary Fig.7. To assess the robustness of our scoring system and control for possible bias introduced by inter-observer variability, we analysed the concordance between the scoring results

obtained by two independent investigators using contingency tables and calculating Cohen's Kappa Indices (Supplementary Fig.11). Finally, the association between PPM1H expression and survival outcomes was tested by a third investigator who did not participate in the scoring process.

Cell culture, siRNA interference, RT-PCR, and Western blot. Human CRC cell lines (CaCO2, SW480, RKO, SW620, and LoVo) and a human colonic fibroblast cell line (CCD-18Co) were purchased from Chinese Academy of Science (Shanghai, China). All the cell lines were authenticated using Short Tandem Repeat (STR) analysis (Genetic Testing Biotechnology Corporation, Suzhou, China). Cells were maintained in Dulbecco's Modified Eagle Medium (GIBCO, Grand Island, NY, USA) supplemented with 10% heat-inactivated foetal bovine serum (FBS) (GIBCO), 100 U/mL penicillin, and 100 μg/mL streptomycin in a 5% CO_2 humidified atmosphere. Negative control siRNA and siRNA targeting PPM1H were designed and synthesized at Shanghai Invitrogen (Shanghai, China). The sequences are presented in Supplementary Table 4. siRNA duplexes were transfected into CaCO2 and SW480 cells in the presence of Lipofectamine® RNAiMAX reagent (Invitrogen, Carlsbad, CA) as previously reported.[3] The expression levels of PPM1H and VIM in CRC cells and CCD-18Co cells were examined by qPCR with LightCycler® 480 II (Roche, Germany) and Western blotting. A rabbit polyclonal antibody against human PPM1H (1:500, SAB1301160, Sigma-Aldrich, St Louis, MO, USA) and VIM (1:500, AV48226, Sigma-Aldrich, St Louis, MO, USA) and rabbit polyclonal antibody against human GAPDH (1:1000, AP0063, Bioworld Technology) were used to detect corresponding protein expression by Western blotting. E-cadherin and N-cadherin were examined only in CRC cells. The PCR primers are presented in Supplementary Table 4.

Cell proliferation assay and invasion assay. Proliferation was quantified after 24, 48, 72, 96 and 120 h using a Cell Counting Kit-8 (Dojindo, Kumamoto, Japan). Cell invasion assays were performed using 24-

well tissue culture plates (8-μm pore size, Transwell, Corning, NY). The bottom of the culture inserts was coated with 20 μg of Matrigel (BD Biosciences, Bedford, MA). Cells (5×10^4) in 0.1 ml medium with 5% FBS were placed in the upper chamber, and the lower chamber was loaded with 0.2 ml conditional media. After culturing for 24 h at 37°C in 5% CO_2, the number of cells that migrated to the lower surface of filters was quantified by counting 5 independent symmetrical visual fields under the microscope. Each assay was performed in triplicate.

Reference

29. Li Q, Birkbak NJ, Gyorffy B, Szallasi Z, Eklund AC. Jetset: selecting the optimal microarray probe set to represent a gene. *BMC Bioinformatics* 2011; **12**: 474.

Supplementary Table 1. 3425 colorectal gene expression array datasets

	DataSets	GSMID	Class	DFS information	KRAS_mut	BRAF_mut	P53_mut	MSI status
1	GSE10714	GSM270790	Cancer	NA	NA	NA	NA	NA
2	GSE10714	GSM270791	Cancer	NA	NA	NA	NA	NA
3	GSE10714	GSM270792	Cancer	NA	NA	NA	NA	NA
4	GSE10714	GSM270793	Cancer	NA	NA	NA	NA	NA
5	GSE10714	GSM270794	Cancer	NA	NA	NA	NA	NA
6	GSE10714	GSM270795	Cancer	NA	NA	NA	NA	NA
7	GSE10714	GSM270796	Cancer	NA	NA	NA	NA	NA
8	GSE13059	GSM327202	Cancer	NA	NA	NA	NA	NA
9	GSE13059	GSM327206	Cancer	NA	NA	NA	NA	NA
10	GSE13059	GSM327210	Cancer	NA	NA	NA	NA	NA
11	GSE13059	GSM327214	Cancer	NA	NA	NA	NA	NA
12	GSE13059	GSM327218	Cancer	NA	NA	NA	NA	NA
13	GSE13059	GSM327221	Cancer	NA	NA	NA	NA	NA
14	GSE13059	GSM327225	Cancer	NA	NA	NA	NA	NA
15	GSE13067	GSM327293	Cancer	NA	NA	NA	NA	MSS
16	GSE13067	GSM327294	Cancer	NA	NA	NA	NA	MSS
17	GSE13067	GSM327310	Cancer	NA	NA	NA	NA	MSI
18	GSE13067	GSM327316	Cancer	NA	NA	NA	NA	MSS
19	GSE13067	GSM327326	Cancer	NA	NA	NA	NA	MSS
20	GSE13067	GSM327329	Cancer	NA	NA	NA	NA	MSS
21	GSE13067	GSM327333	Cancer	NA	NA	NA	NA	MSS
22	GSE13067	GSM327337	Cancer	NA	NA	NA	NA	MSS
23	GSE13067	GSM327342	Cancer	NA	NA	NA	NA	MSS
24	GSE13067	GSM327352	Cancer	NA	NA	NA	NA	MSI
25	GSE13294	GSM335510	Cancer	NA	NA	NA	NA	MSS
26	GSE13294	GSM335511	Cancer	NA	NA	NA	NA	MSI
27	GSE13294	GSM335512	Cancer	NA	NA	NA	NA	MSS
28	GSE13294	GSM335513	Cancer	NA	NA	NA	NA	MSS
29	GSE13294	GSM335514	Cancer	NA	NA	NA	NA	MSS
30	GSE13294	GSM335515	Cancer	NA	NA	NA	NA	MSS
31	GSE13294	GSM335516	Cancer	NA	NA	NA	NA	MSS
32	GSE13294	GSM335517	Cancer	NA	NA	NA	NA	MSS
33	GSE13294	GSM335518	Cancer	NA	NA	NA	NA	MSI
34	GSE13294	GSM335519	Cancer	NA	NA	NA	NA	MSI
35	GSE13294	GSM335520	Cancer	NA	NA	NA	NA	MSS
36	GSE13294	GSM335521	Cancer	NA	NA	NA	NA	MSS
37	GSE13294	GSM335522	Cancer	NA	NA	NA	NA	MSS
38	GSE13294	GSM335523	Cancer	NA	NA	NA	NA	MSS
39	GSE13294	GSM335524	Cancer	NA	NA	NA	NA	MSS
40	GSE13294	GSM335525	Cancer	NA	NA	NA	NA	MSI
41	GSE13294	GSM335526	Cancer	NA	NA	NA	NA	MSS
42	GSE13294	GSM335527	Cancer	NA	NA	NA	NA	MSI

43	GSE13294	GSM335528	Cancer	NA	NA	NA	NA	MSS
44	GSE13294	GSM335529	Cancer	NA	NA	NA	NA	MSI
45	GSE13294	GSM335530	Cancer	NA	NA	NA	NA	MSI
46	GSE13294	GSM335531	Cancer	NA	NA	NA	NA	MSS
47	GSE13294	GSM335532	Cancer	NA	NA	NA	NA	MSI
48	GSE13294	GSM335533	Cancer	NA	NA	NA	NA	MSS
49	GSE13294	GSM335534	Cancer	NA	NA	NA	NA	MSS
50	GSE13294	GSM335535	Cancer	NA	NA	NA	NA	MSS
51	GSE13294	GSM335536	Cancer	NA	NA	NA	NA	MSS
52	GSE13294	GSM335537	Cancer	NA	NA	NA	NA	MSS
53	GSE13294	GSM335538	Cancer	NA	NA	NA	NA	MSS
54	GSE13294	GSM335539	Cancer	NA	NA	NA	NA	MSS
55	GSE13294	GSM335540	Cancer	NA	NA	NA	NA	MSS
56	GSE13294	GSM335541	Cancer	NA	NA	NA	NA	MSI
57	GSE13294	GSM335542	Cancer	NA	NA	NA	NA	MSI
58	GSE13294	GSM335543	Cancer	NA	NA	NA	NA	MSI
59	GSE13294	GSM335544	Cancer	NA	NA	NA	NA	MSI
60	GSE13294	GSM335545	Cancer	NA	NA	NA	NA	MSI
61	GSE13294	GSM335546	Cancer	NA	NA	NA	NA	MSI
62	GSE13294	GSM335547	Cancer	NA	NA	NA	NA	MSS
63	GSE13294	GSM335548	Cancer	NA	NA	NA	NA	MSS
64	GSE13294	GSM335549	Cancer	NA	NA	NA	NA	MSS
65	GSE13294	GSM335550	Cancer	NA	NA	NA	NA	MSS
66	GSE13294	GSM335551	Cancer	NA	NA	NA	NA	MSI
67	GSE13294	GSM335552	Cancer	NA	NA	NA	NA	MSS
68	GSE13294	GSM335553	Cancer	NA	NA	NA	NA	MSS
69	GSE13294	GSM335554	Cancer	NA	NA	NA	NA	MSS
70	GSE13294	GSM335555	Cancer	NA	NA	NA	NA	MSI
71	GSE13294	GSM335556	Cancer	NA	NA	NA	NA	MSS
72	GSE13294	GSM335557	Cancer	NA	NA	NA	NA	MSS
73	GSE13294	GSM335558	Cancer	NA	NA	NA	NA	MSS
74	GSE13294	GSM335559	Cancer	NA	NA	NA	NA	MSI
75	GSE13294	GSM335560	Cancer	NA	NA	NA	NA	MSI
76	GSE13294	GSM335561	Cancer	NA	NA	NA	NA	MSS
77	GSE13294	GSM335562	Cancer	NA	NA	NA	NA	MSI
78	GSE13294	GSM335563	Cancer	NA	NA	NA	NA	MSI
79	GSE13294	GSM335564	Cancer	NA	NA	NA	NA	MSS
80	GSE13294	GSM335565	Cancer	NA	NA	NA	NA	MSI
81	GSE13294	GSM335566	Cancer	NA	NA	NA	NA	MSI
82	GSE13294	GSM335567	Cancer	NA	NA	NA	NA	MSS
83	GSE13294	GSM335568	Cancer	NA	NA	NA	NA	MSS
84	GSE13294	GSM335569	Cancer	NA	NA	NA	NA	MSS
85	GSE13294	GSM335570	Cancer	NA	NA	NA	NA	MSS
86	GSE13294	GSM335571	Cancer	NA	NA	NA	NA	MSS
87	GSE13294	GSM335572	Cancer	NA	NA	NA	NA	MSS

88	GSE13294	GSM335573	Cancer	NA	NA	NA	NA	MSI
89	GSE13294	GSM335574	Cancer	NA	NA	NA	NA	MSI
90	GSE13294	GSM335575	Cancer	NA	NA	NA	NA	MSS
91	GSE13294	GSM335576	Cancer	NA	NA	NA	NA	MSI
92	GSE13294	GSM335577	Cancer	NA	NA	NA	NA	MSI
93	GSE13294	GSM335578	Cancer	NA	NA	NA	NA	MSI
94	GSE13294	GSM335579	Cancer	NA	NA	NA	NA	MSS
95	GSE13294	GSM335580	Cancer	NA	NA	NA	NA	MSS
96	GSE13294	GSM335581	Cancer	NA	NA	NA	NA	MSS
97	GSE13294	GSM335582	Cancer	NA	NA	NA	NA	MSS
98	GSE13294	GSM335583	Cancer	NA	NA	NA	NA	MSS
99	GSE13294	GSM335584	Cancer	NA	NA	NA	NA	MSS
100	GSE13294	GSM335585	Cancer	NA	NA	NA	NA	MSI
101	GSE13294	GSM335586	Cancer	NA	NA	NA	NA	MSS
102	GSE13294	GSM335587	Cancer	NA	NA	NA	NA	MSS
103	GSE13294	GSM335588	Cancer	NA	NA	NA	NA	MSS
104	GSE13294	GSM335589	Cancer	NA	NA	NA	NA	MSS
105	GSE13294	GSM335590	Cancer	NA	NA	NA	NA	MSS
106	GSE13294	GSM335591	Cancer	NA	NA	NA	NA	MSS
107	GSE13294	GSM335592	Cancer	NA	NA	NA	NA	MSS
108	GSE13294	GSM335593	Cancer	NA	NA	NA	NA	MSS
109	GSE13294	GSM335594	Cancer	NA	NA	NA	NA	MSS
110	GSE13294	GSM335595	Cancer	NA	NA	NA	NA	MSI
111	GSE13294	GSM335596	Cancer	NA	NA	NA	NA	MSS
112	GSE13294	GSM335597	Cancer	NA	NA	NA	NA	MSI
113	GSE13294	GSM335598	Cancer	NA	NA	NA	NA	MSS
114	GSE13294	GSM335599	Cancer	NA	NA	NA	NA	MSS
115	GSE13294	GSM335600	Cancer	NA	NA	NA	NA	MSS
116	GSE13294	GSM335601	Cancer	NA	NA	NA	NA	MSS
117	GSE13294	GSM335602	Cancer	NA	NA	NA	NA	MSS
118	GSE13294	GSM335603	Cancer	NA	NA	NA	NA	MSS
119	GSE13294	GSM335604	Cancer	NA	NA	NA	NA	MSS
120	GSE13294	GSM335605	Cancer	NA	NA	NA	NA	MSS
121	GSE13294	GSM335606	Cancer	NA	NA	NA	NA	MSS
122	GSE13294	GSM335607	Cancer	NA	NA	NA	NA	MSS
123	GSE13294	GSM335608	Cancer	NA	NA	NA	NA	MSS
124	GSE13294	GSM335609	Cancer	NA	NA	NA	NA	MSS
125	GSE13294	GSM335610	Cancer	NA	NA	NA	NA	MSS
126	GSE13294	GSM335611	Cancer	NA	NA	NA	NA	MSS
127	GSE13294	GSM335612	Cancer	NA	NA	NA	NA	MSS
128	GSE13294	GSM335613	Cancer	NA	NA	NA	NA	MSS
129	GSE13294	GSM335614	Cancer	NA	NA	NA	NA	MSI
130	GSE13294	GSM335615	Cancer	NA	NA	NA	NA	MSS
131	GSE13294	GSM335616	Cancer	NA	NA	NA	NA	MSS
132	GSE13294	GSM335617	Cancer	NA	NA	NA	NA	MSS

133	GSE13294	GSM335618	Cancer	NA	NA	NA	NA	MSI
134	GSE13294	GSM335619	Cancer	NA	NA	NA	NA	MSI
135	GSE13294	GSM335620	Cancer	NA	NA	NA	NA	MSI
136	GSE13294	GSM335621	Cancer	NA	NA	NA	NA	MSI
137	GSE13294	GSM335622	Cancer	NA	NA	NA	NA	MSI
138	GSE13294	GSM335623	Cancer	NA	NA	NA	NA	MSI
139	GSE13294	GSM335624	Cancer	NA	NA	NA	NA	MSI
140	GSE13294	GSM335625	Cancer	NA	NA	NA	NA	MSI
141	GSE13294	GSM335626	Cancer	NA	NA	NA	NA	MSI
142	GSE13294	GSM335627	Cancer	NA	NA	NA	NA	MSI
143	GSE13294	GSM335628	Cancer	NA	NA	NA	NA	MSI
144	GSE13294	GSM335629	Cancer	NA	NA	NA	NA	MSI
145	GSE13294	GSM335630	Cancer	NA	NA	NA	NA	MSI
146	GSE13294	GSM335631	Cancer	NA	NA	NA	NA	MSI
147	GSE13294	GSM335632	Cancer	NA	NA	NA	NA	MSI
148	GSE13294	GSM335633	Cancer	NA	NA	NA	NA	MSI
149	GSE13294	GSM335634	Cancer	NA	NA	NA	NA	MSI
150	GSE13294	GSM335635	Cancer	NA	NA	NA	NA	MSI
151	GSE13294	GSM335636	Cancer	NA	NA	NA	NA	MSI
152	GSE13294	GSM335638	Cancer	NA	NA	NA	NA	MSI
153	GSE13294	GSM335639	Cancer	NA	NA	NA	NA	MSI
154	GSE13294	GSM335640	Cancer	NA	NA	NA	NA	MSI
155	GSE13294	GSM335641	Cancer	NA	NA	NA	NA	MSI
156	GSE13294	GSM335642	Cancer	NA	NA	NA	NA	MSI
157	GSE13294	GSM335643	Cancer	NA	NA	NA	NA	MSI
158	GSE13294	GSM335644	Cancer	NA	NA	NA	NA	MSI
159	GSE13294	GSM335645	Cancer	NA	NA	NA	NA	MSI
160	GSE13294	GSM335646	Cancer	NA	NA	NA	NA	MSI
161	GSE13294	GSM335647	Cancer	NA	NA	NA	NA	MSI
162	GSE13294	GSM335648	Cancer	NA	NA	NA	NA	MSI
163	GSE13294	GSM335649	Cancer	NA	NA	NA	NA	MSI
164	GSE13294	GSM335650	Cancer	NA	NA	NA	NA	MSI
165	GSE13294	GSM335651	Cancer	NA	NA	NA	NA	MSI
166	GSE13294	GSM335652	Cancer	NA	NA	NA	NA	MSI
167	GSE13294	GSM335653	Cancer	NA	NA	NA	NA	MSI
168	GSE13294	GSM335654	Cancer	NA	NA	NA	NA	MSI
169	GSE13294	GSM335655	Cancer	NA	NA	NA	NA	MSI
170	GSE13294	GSM335656	Cancer	NA	NA	NA	NA	MSI
171	GSE13294	GSM335657	Cancer	NA	NA	NA	NA	MSI
172	GSE13294	GSM335658	Cancer	NA	NA	NA	NA	MSI
173	GSE13294	GSM335659	Cancer	NA	NA	NA	NA	MSI
174	GSE13294	GSM335660	Cancer	NA	NA	NA	NA	MSI
175	GSE13294	GSM335661	Cancer	NA	NA	NA	NA	MSI
176	GSE13294	GSM335662	Cancer	NA	NA	NA	NA	MSI
177	GSE13294	GSM335663	Cancer	NA	NA	NA	NA	MSI

178	GSE13471	GSM339562	Cancer	NA	NA	NA	NA	NA
179	GSE13471	GSM339565	Cancer	NA	NA	NA	NA	NA
180	GSE13471	GSM339566	Cancer	NA	NA	NA	NA	NA
181	GSE13471	GSM339567	Cancer	NA	NA	NA	NA	NA
182	GSE14333	GSM358341	Cancer	Avaliable	NA	NA	NA	NA
183	GSE14333	GSM358342	Cancer	Avaliable	NA	NA	NA	NA
184	GSE14333	GSM358343	Cancer	Avaliable	NA	NA	NA	NA
185	GSE14333	GSM358344	Cancer	Avaliable	NA	NA	NA	NA
186	GSE14333	GSM358345	Cancer	Avaliable	NA	NA	NA	NA
187	GSE14333	GSM358346	Cancer	Avaliable	NA	NA	NA	NA
188	GSE14333	GSM358347	Cancer	Avaliable	NA	NA	NA	NA
189	GSE14333	GSM358348	Cancer	Avaliable	NA	NA	NA	NA
190	GSE14333	GSM358349	Cancer	Avaliable	NA	NA	NA	NA
191	GSE14333	GSM358350	Cancer	Avaliable	NA	NA	NA	NA
192	GSE14333	GSM358351	Cancer	Avaliable	NA	NA	NA	NA
193	GSE14333	GSM358352	Cancer	Avaliable	NA	NA	NA	NA
194	GSE14333	GSM358353	Cancer	Avaliable	NA	NA	NA	NA
195	GSE14333	GSM358354	Cancer	Avaliable	NA	NA	NA	NA
196	GSE14333	GSM358355	Cancer	Avaliable	NA	NA	NA	NA
197	GSE14333	GSM358356	Cancer	Avaliable	NA	NA	NA	NA
198	GSE14333	GSM358357	Cancer	Avaliable	NA	NA	NA	NA
199	GSE14333	GSM358358	Cancer	Avaliable	NA	NA	NA	NA
200	GSE14333	GSM358359	Cancer	Avaliable	NA	NA	NA	NA
201	GSE14333	GSM358360	Cancer	Avaliable	NA	NA	NA	NA
202	GSE14333	GSM358361	Cancer	Avaliable	NA	NA	NA	NA
203	GSE14333	GSM358362	Cancer	Avaliable	NA	NA	NA	NA
204	GSE14333	GSM358363	Cancer	Avaliable	NA	NA	NA	NA
205	GSE14333	GSM358364	Cancer	Avaliable	NA	NA	NA	NA
206	GSE14333	GSM358365	Cancer	Avaliable	NA	NA	NA	NA
207	GSE14333	GSM358366	Cancer	Avaliable	NA	NA	NA	NA
208	GSE14333	GSM358367	Cancer	Avaliable	NA	NA	NA	NA
209	GSE14333	GSM358368	Cancer	Avaliable	NA	NA	NA	NA
210	GSE14333	GSM358369	Cancer	NA	NA	NA	NA	NA
211	GSE14333	GSM358370	Cancer	Avaliable	NA	NA	NA	MSS
212	GSE14333	GSM358371	Cancer	Avaliable	NA	NA	NA	MSS
213	GSE14333	GSM358372	Cancer	NA	NA	NA	NA	MSS
214	GSE14333	GSM358373	Cancer	Avaliable	NA	NA	NA	MSS
215	GSE14333	GSM358374	Cancer	Avaliable	NA	NA	NA	MSS
216	GSE14333	GSM358375	Cancer	Avaliable	NA	NA	NA	MSS
217	GSE14333	GSM358376	Cancer	Avaliable	NA	NA	NA	NA
218	GSE14333	GSM358377	Cancer	Avaliable	NA	NA	NA	NA
219	GSE14333	GSM358378	Cancer	Avaliable	NA	NA	NA	NA
220	GSE14333	GSM358379	Cancer	NA	NA	NA	NA	NA
221	GSE14333	GSM358380	Cancer	Avaliable	NA	NA	NA	NA
222	GSE14333	GSM358381	Cancer	Avaliable	NA	NA	NA	NA

223	GSE14333	GSM358382	Cancer	Avaliable	NA	NA	NA	NA
224	GSE14333	GSM358384	Cancer	Avaliable	NA	NA	NA	NA
225	GSE14333	GSM358385	Cancer	Avaliable	NA	NA	NA	NA
226	GSE14333	GSM358386	Cancer	Avaliable	NA	NA	NA	NA
227	GSE14333	GSM358387	Cancer	Avaliable	NA	NA	NA	NA
228	GSE14333	GSM358388	Cancer	Avaliable	NA	NA	NA	NA
229	GSE14333	GSM358389	Cancer	Avaliable	NA	NA	NA	NA
230	GSE14333	GSM358390	Cancer	Avaliable	NA	NA	NA	NA
231	GSE14333	GSM358391	Cancer	Avaliable	NA	NA	NA	NA
232	GSE14333	GSM358392	Cancer	Avaliable	NA	NA	NA	NA
233	GSE14333	GSM358393	Cancer	Avaliable	NA	NA	NA	NA
234	GSE14333	GSM358394	Cancer	Avaliable	NA	NA	NA	NA
235	GSE14333	GSM358395	Cancer	Avaliable	NA	NA	NA	NA
236	GSE14333	GSM358396	Cancer	Avaliable	NA	NA	NA	NA
237	GSE14333	GSM358397	Cancer	Avaliable	NA	NA	NA	NA
238	GSE14333	GSM358398	Cancer	Avaliable	NA	NA	NA	NA
239	GSE14333	GSM358399	Cancer	Avaliable	NA	NA	NA	NA
240	GSE14333	GSM358400	Cancer	Avaliable	NA	NA	NA	NA
241	GSE14333	GSM358401	Cancer	Avaliable	NA	NA	NA	NA
242	GSE14333	GSM358402	Cancer	Avaliable	NA	NA	NA	NA
243	GSE14333	GSM358403	Cancer	Avaliable	NA	NA	NA	NA
244	GSE14333	GSM358404	Cancer	Avaliable	NA	NA	NA	NA
245	GSE14333	GSM358405	Cancer	Avaliable	NA	NA	NA	NA
246	GSE14333	GSM358406	Cancer	Avaliable	NA	NA	NA	NA
247	GSE14333	GSM358407	Cancer	Avaliable	NA	NA	NA	NA
248	GSE14333	GSM358408	Cancer	Avaliable	NA	NA	NA	NA
249	GSE14333	GSM358409	Cancer	Avaliable	NA	NA	NA	NA
250	GSE14333	GSM358410	Cancer	Avaliable	NA	NA	NA	NA
251	GSE14333	GSM358411	Cancer	Avaliable	NA	NA	NA	NA
252	GSE14333	GSM358412	Cancer	Avaliable	NA	NA	NA	NA
253	GSE14333	GSM358413	Cancer	Avaliable	NA	NA	NA	NA
254	GSE14333	GSM358414	Cancer	Avaliable	NA	NA	NA	NA
255	GSE14333	GSM358415	Cancer	Avaliable	NA	NA	NA	NA
256	GSE14333	GSM358416	Cancer	Avaliable	NA	NA	NA	NA
257	GSE14333	GSM358417	Cancer	Avaliable	NA	NA	NA	NA
258	GSE14333	GSM358418	Cancer	Avaliable	NA	NA	NA	NA
259	GSE14333	GSM358419	Cancer	Avaliable	NA	NA	NA	NA
260	GSE14333	GSM358420	Cancer	Avaliable	NA	NA	NA	NA
261	GSE14333	GSM358421	Cancer	Avaliable	NA	NA	NA	NA
262	GSE14333	GSM358422	Cancer	Avaliable	NA	NA	NA	NA
263	GSE14333	GSM358423	Cancer	Avaliable	NA	NA	NA	NA
264	GSE14333	GSM358424	Cancer	Avaliable	NA	NA	NA	NA
265	GSE14333	GSM358425	Cancer	Avaliable	NA	NA	NA	NA
266	GSE14333	GSM358426	Cancer	Avaliable	NA	NA	NA	NA
267	GSE14333	GSM358427	Cancer	Avaliable	NA	NA	NA	NA

268	GSE14333	GSM358428	Cancer	Avaliable	NA	NA	NA	NA
269	GSE14333	GSM358429	Cancer	Avaliable	NA	NA	NA	NA
270	GSE14333	GSM358430	Cancer	Avaliable	NA	NA	NA	NA
271	GSE14333	GSM358431	Cancer	Avaliable	NA	NA	NA	NA
272	GSE14333	GSM358432	Cancer	Avaliable	NA	NA	NA	NA
273	GSE14333	GSM358433	Cancer	Avaliable	NA	NA	NA	NA
274	GSE14333	GSM358434	Cancer	Avaliable	NA	NA	NA	NA
275	GSE14333	GSM358435	Cancer	Avaliable	NA	NA	NA	NA
276	GSE14333	GSM358436	Cancer	Avaliable	NA	NA	NA	NA
277	GSE14333	GSM358437	Cancer	Avaliable	NA	NA	NA	NA
278	GSE14333	GSM358438	Cancer	Avaliable	NA	NA	NA	NA
279	GSE14333	GSM358439	Cancer	Avaliable	NA	NA	NA	NA
280	GSE14333	GSM358440	Cancer	Avaliable	NA	NA	NA	NA
281	GSE14333	GSM358441	Cancer	Avaliable	NA	NA	NA	NA
282	GSE14333	GSM358442	Cancer	Avaliable	NA	NA	NA	MSI
283	GSE14333	GSM358443	Cancer	Avaliable	NA	NA	NA	MSS
284	GSE14333	GSM358444	Cancer	Avaliable	NA	NA	NA	MSS
285	GSE14333	GSM358445	Cancer	Avaliable	NA	NA	NA	MSS
286	GSE14333	GSM358446	Cancer	Avaliable	NA	NA	NA	MSS
287	GSE14333	GSM358447	Cancer	Avaliable	NA	NA	NA	MSS
288	GSE14333	GSM358448	Cancer	Avaliable	NA	NA	NA	NA
289	GSE14333	GSM358449	Cancer	Avaliable	NA	NA	NA	MSS
290	GSE14333	GSM358450	Cancer	Avaliable	NA	NA	NA	MSS
291	GSE14333	GSM358451	Cancer	Avaliable	NA	NA	NA	MSS
292	GSE14333	GSM358452	Cancer	Avaliable	NA	NA	NA	MSS
293	GSE14333	GSM358453	Cancer	Avaliable	NA	NA	NA	MSI
294	GSE14333	GSM358454	Cancer	Avaliable	NA	NA	NA	MSI
295	GSE14333	GSM358455	Cancer	Avaliable	NA	NA	NA	MSS
296	GSE14333	GSM358456	Cancer	Avaliable	NA	NA	NA	MSS
297	GSE14333	GSM358457	Cancer	Avaliable	NA	NA	NA	MSI
298	GSE14333	GSM358458	Cancer	Avaliable	NA	NA	NA	MSI
299	GSE14333	GSM358459	Cancer	Avaliable	NA	NA	NA	NA
300	GSE14333	GSM358460	Cancer	Avaliable	NA	NA	NA	NA
301	GSE14333	GSM358461	Cancer	Avaliable	NA	NA	NA	NA
302	GSE14333	GSM358462	Cancer	Avaliable	NA	NA	NA	NA
303	GSE14333	GSM358463	Cancer	Avaliable	NA	NA	NA	NA
304	GSE14333	GSM358464	Cancer	Avaliable	NA	NA	NA	MSS
305	GSE14333	GSM358465	Cancer	Avaliable	NA	NA	NA	MSS
306	GSE14333	GSM358466	Cancer	Avaliable	NA	NA	NA	MSS
307	GSE14333	GSM358467	Cancer	Avaliable	NA	NA	NA	NA
308	GSE14333	GSM358468	Cancer	Avaliable	NA	NA	NA	NA
309	GSE14333	GSM358469	Cancer	Avaliable	NA	NA	NA	NA
310	GSE14333	GSM358470	Cancer	Avaliable	NA	NA	NA	NA
311	GSE14333	GSM358471	Cancer	Avaliable	NA	NA	NA	NA
312	GSE14333	GSM358472	Cancer	Avaliable	NA	NA	NA	NA

313	GSE14333	GSM358473	Cancer	Avaliable	NA	NA	NA	NA
314	GSE14333	GSM358474	Cancer	Avaliable	NA	NA	NA	NA
315	GSE14333	GSM358475	Cancer	Avaliable	NA	NA	NA	NA
316	GSE14333	GSM358476	Cancer	Avaliable	NA	NA	NA	NA
317	GSE14333	GSM358477	Cancer	Avaliable	NA	NA	NA	NA
318	GSE14333	GSM358478	Cancer	Avaliable	NA	NA	NA	NA
319	GSE14333	GSM358479	Cancer	Avaliable	NA	NA	NA	NA
320	GSE14333	GSM358480	Cancer	Avaliable	NA	NA	NA	NA
321	GSE14333	GSM358481	Cancer	Avaliable	NA	NA	NA	NA
322	GSE14333	GSM358482	Cancer	Avaliable	NA	NA	NA	NA
323	GSE14333	GSM358483	Cancer	Avaliable	NA	NA	NA	NA
324	GSE14333	GSM358484	Cancer	Avaliable	NA	NA	NA	NA
325	GSE14333	GSM358485	Cancer	Avaliable	NA	NA	NA	NA
326	GSE14333	GSM358486	Cancer	Avaliable	NA	NA	NA	NA
327	GSE14333	GSM358487	Cancer	Avaliable	NA	NA	NA	NA
328	GSE14333	GSM358488	Cancer	Avaliable	NA	NA	NA	NA
329	GSE14333	GSM358489	Cancer	Avaliable	NA	NA	NA	NA
330	GSE14333	GSM358490	Cancer	Avaliable	NA	NA	NA	NA
331	GSE14333	GSM358491	Cancer	Avaliable	NA	NA	NA	NA
332	GSE14333	GSM358492	Cancer	Avaliable	NA	NA	NA	NA
333	GSE14333	GSM358493	Cancer	Avaliable	NA	NA	NA	NA
334	GSE14333	GSM358494	Cancer	Avaliable	NA	NA	NA	NA
335	GSE14333	GSM358495	Cancer	Avaliable	NA	NA	NA	NA
336	GSE14333	GSM358496	Cancer	Avaliable	NA	NA	NA	NA
337	GSE14333	GSM358497	Cancer	Avaliable	NA	NA	NA	NA
338	GSE14333	GSM358498	Cancer	Avaliable	NA	NA	NA	NA
339	GSE14333	GSM358499	Cancer	Avaliable	NA	NA	NA	NA
340	GSE14333	GSM358500	Cancer	Avaliable	NA	NA	NA	NA
341	GSE14333	GSM358501	Cancer	Avaliable	NA	NA	NA	NA
342	GSE14333	GSM358502	Cancer	Avaliable	NA	NA	NA	NA
343	GSE14333	GSM358503	Cancer	Avaliable	NA	NA	NA	NA
344	GSE14333	GSM358504	Cancer	Avaliable	NA	NA	NA	NA
345	GSE14333	GSM358505	Cancer	Avaliable	NA	NA	NA	NA
346	GSE14333	GSM358506	Cancer	Avaliable	NA	NA	NA	NA
347	GSE14333	GSM358507	Cancer	Avaliable	NA	NA	NA	NA
348	GSE14333	GSM358508	Cancer	Avaliable	NA	NA	NA	NA
349	GSE14333	GSM358509	Cancer	Avaliable	NA	NA	NA	NA
350	GSE14333	GSM358511	Cancer	Avaliable	NA	NA	NA	NA
351	GSE14333	GSM358512	Cancer	Avaliable	NA	NA	NA	NA
352	GSE14333	GSM358513	Cancer	Avaliable	NA	NA	NA	NA
353	GSE14333	GSM358514	Cancer	Avaliable	NA	NA	NA	NA
354	GSE14333	GSM358515	Cancer	Avaliable	NA	NA	NA	NA
355	GSE14333	GSM358516	Cancer	Avaliable	NA	NA	NA	NA
356	GSE14333	GSM358517	Cancer	Avaliable	NA	NA	NA	NA
357	GSE14333	GSM358518	Cancer	Avaliable	NA	NA	NA	NA

358	GSE14333	GSM358519	Cancer	Avaliable	NA	NA	NA	NA
359	GSE14333	GSM358520	Cancer	Avaliable	NA	NA	NA	NA
360	GSE14333	GSM358521	Cancer	Avaliable	NA	NA	NA	NA
361	GSE14333	GSM358522	Cancer	Avaliable	NA	NA	NA	NA
362	GSE14333	GSM358523	Cancer	Avaliable	NA	NA	NA	NA
363	GSE14333	GSM358524	Cancer	Avaliable	NA	NA	NA	NA
364	GSE14333	GSM358525	Cancer	Avaliable	NA	NA	NA	NA
365	GSE14333	GSM358526	Cancer	Avaliable	NA	NA	NA	NA
366	GSE14333	GSM358527	Cancer	Avaliable	NA	NA	NA	NA
367	GSE14333	GSM358528	Cancer	Avaliable	NA	NA	NA	NA
368	GSE14333	GSM358529	Cancer	Avaliable	NA	NA	NA	NA
369	GSE14333	GSM358530	Cancer	Avaliable	NA	NA	NA	NA
370	GSE14333	GSM358531	Cancer	Avaliable	NA	NA	NA	NA
371	GSE14333	GSM358532	Cancer	Avaliable	NA	NA	NA	NA
372	GSE14333	GSM358533	Cancer	Avaliable	NA	NA	NA	NA
373	GSE14333	GSM358534	Cancer	Avaliable	NA	NA	NA	NA
374	GSE14333	GSM358535	Cancer	Avaliable	NA	NA	NA	NA
375	GSE14333	GSM358536	Cancer	Avaliable	NA	NA	NA	NA
376	GSE14333	GSM358537	Cancer	Avaliable	NA	NA	NA	NA
377	GSE14333	GSM358538	Cancer	Avaliable	NA	NA	NA	NA
378	GSE14333	GSM358539	Cancer	Avaliable	NA	NA	NA	NA
379	GSE14333	GSM358540	Cancer	Avaliable	NA	NA	NA	MSS
380	GSE14333	GSM358541	Cancer	Avaliable	NA	NA	NA	MSS
381	GSE14333	GSM358542	Cancer	Avaliable	NA	NA	NA	MSS
382	GSE14333	GSM358543	Cancer	Avaliable	NA	NA	NA	MSS
383	GSE14333	GSM358544	Cancer	Avaliable	NA	NA	NA	MSS
384	GSE14333	GSM358545	Cancer	Avaliable	NA	NA	NA	MSS
385	GSE14333	GSM358546	Cancer	Avaliable	NA	NA	NA	MSI
386	GSE14333	GSM358547	Cancer	Avaliable	NA	NA	NA	MSS
387	GSE14333	GSM358548	Cancer	Avaliable	NA	NA	NA	MSS
388	GSE14333	GSM358549	Cancer	Avaliable	NA	NA	NA	MSS
389	GSE14333	GSM358550	Cancer	Avaliable	NA	NA	NA	MSS
390	GSE14333	GSM358551	Cancer	Avaliable	NA	NA	NA	MSI
391	GSE14333	GSM358552	Cancer	Avaliable	NA	NA	NA	MSS
392	GSE14333	GSM358553	Cancer	Avaliable	NA	NA	NA	MSS
393	GSE14333	GSM358554	Cancer	Avaliable	NA	NA	NA	MSI
394	GSE14333	GSM358555	Cancer	Avaliable	NA	NA	NA	MSS
395	GSE14333	GSM358556	Cancer	Avaliable	NA	NA	NA	MSS
396	GSE14333	GSM358557	Cancer	Avaliable	NA	NA	NA	MSS
397	GSE14333	GSM358558	Cancer	Avaliable	NA	NA	NA	MSS
398	GSE14333	GSM358559	Cancer	Avaliable	NA	NA	NA	MSI
399	GSE14333	GSM358560	Cancer	Avaliable	NA	NA	NA	MSS
400	GSE14333	GSM358561	Cancer	Avaliable	NA	NA	NA	MSS
401	GSE14333	GSM358562	Cancer	Avaliable	NA	NA	NA	MSS
402	GSE14333	GSM358563	Cancer	Avaliable	NA	NA	NA	MSS

403	GSE14333	GSM358564	Cancer	Avaliable	NA	NA	NA	MSS
404	GSE14333	GSM358565	Cancer	Avaliable	NA	NA	NA	NA
405	GSE14333	GSM358566	Cancer	Avaliable	NA	NA	NA	NA
406	GSE14333	GSM358567	Cancer	Avaliable	NA	NA	NA	NA
407	GSE14333	GSM358568	Cancer	Avaliable	NA	NA	NA	NA
408	GSE14333	GSM358569	Cancer	Avaliable	NA	NA	NA	NA
409	GSE14333	GSM358570	Cancer	NA	NA	NA	NA	NA
410	GSE14333	GSM358571	Cancer	NA	NA	NA	NA	NA
411	GSE14333	GSM358572	Cancer	NA	NA	NA	NA	NA
412	GSE14333	GSM358573	Cancer	NA	NA	NA	NA	NA
413	GSE14333	GSM358574	Cancer	NA	NA	NA	NA	NA
414	GSE14333	GSM358575	Cancer	NA	NA	NA	NA	NA
415	GSE14333	GSM358576	Cancer	NA	NA	NA	NA	NA
416	GSE14333	GSM358577	Cancer	NA	NA	NA	NA	NA
417	GSE14333	GSM358578	Cancer	NA	NA	NA	NA	NA
418	GSE14333	GSM358579	Cancer	NA	NA	NA	NA	NA
419	GSE14333	GSM358580	Cancer	NA	NA	NA	NA	NA
420	GSE14333	GSM358581	Cancer	NA	NA	NA	NA	NA
421	GSE14333	GSM358582	Cancer	NA	NA	NA	NA	NA
422	GSE14333	GSM358583	Cancer	NA	NA	NA	NA	NA
423	GSE14333	GSM358584	Cancer	NA	NA	NA	NA	NA
424	GSE14333	GSM358585	Cancer	NA	NA	NA	NA	NA
425	GSE14333	GSM358586	Cancer	NA	NA	NA	NA	NA
426	GSE14333	GSM358588	Cancer	NA	NA	NA	NA	NA
427	GSE14333	GSM358589	Cancer	NA	NA	NA	NA	NA
428	GSE14333	GSM358590	Cancer	NA	NA	NA	NA	NA
429	GSE14333	GSM358591	Cancer	NA	NA	NA	NA	NA
430	GSE14333	GSM358592	Cancer	NA	NA	NA	NA	NA
431	GSE14333	GSM358593	Cancer	NA	NA	NA	NA	NA
432	GSE14333	GSM358595	Cancer	NA	NA	NA	NA	NA
433	GSE14333	GSM358596	Cancer	NA	NA	NA	NA	NA
434	GSE14333	GSM358597	Cancer	NA	NA	NA	NA	NA
435	GSE14333	GSM358598	Cancer	NA	NA	NA	NA	NA
436	GSE14333	GSM358599	Cancer	NA	NA	NA	NA	NA
437	GSE14333	GSM358600	Cancer	NA	NA	NA	NA	NA
438	GSE14333	GSM358601	Cancer	NA	NA	NA	NA	NA
439	GSE14333	GSM358602	Cancer	NA	NA	NA	NA	NA
440	GSE14333	GSM358603	Cancer	NA	NA	NA	NA	NA
441	GSE14333	GSM358604	Cancer	NA	NA	NA	NA	NA
442	GSE14333	GSM358605	Cancer	NA	NA	NA	NA	NA
443	GSE14333	GSM358606	Cancer	NA	NA	NA	NA	NA
444	GSE14333	GSM358607	Cancer	NA	NA	NA	NA	NA
445	GSE14333	GSM358608	Cancer	NA	NA	NA	NA	NA
446	GSE14333	GSM358609	Cancer	NA	NA	NA	NA	NA
447	GSE14333	GSM358610	Cancer	NA	NA	NA	NA	NA

448	GSE14333	GSM358611	Cancer	NA	NA	NA	NA	NA
449	GSE14333	GSM358612	Cancer	NA	NA	NA	NA	MSS
450	GSE14333	GSM358613	Cancer	NA	NA	NA	NA	MSS
451	GSE14333	GSM358614	Cancer	NA	NA	NA	NA	MSS
452	GSE14333	GSM358615	Cancer	NA	NA	NA	NA	MSS
453	GSE14333	GSM358616	Cancer	NA	NA	NA	NA	MSS
454	GSE14333	GSM358617	Cancer	NA	NA	NA	NA	MSS
455	GSE14333	GSM358618	Cancer	NA	NA	NA	NA	MSS
456	GSE14333	GSM358619	Cancer	NA	NA	NA	NA	MSS
457	GSE14333	GSM358620	Cancer	NA	NA	NA	NA	MSS
458	GSE14333	GSM358622	Cancer	NA	NA	NA	NA	MSS
459	GSE14333	GSM358623	Cancer	NA	NA	NA	NA	MSS
460	GSE14333	GSM358624	Cancer	NA	NA	NA	NA	MSS
461	GSE14333	GSM358625	Cancer	NA	NA	NA	NA	NA
462	GSE14333	GSM358626	Cancer	NA	NA	NA	NA	NA
463	GSE14333	GSM358627	Cancer	NA	NA	NA	NA	MSS
464	GSE14333	GSM358628	Cancer	NA	NA	NA	NA	NA
465	GSE14333	GSM358629	Cancer	NA	NA	NA	NA	NA
466	GSE14333	GSM358630	Cancer	NA	NA	NA	NA	NA
467	GSE17538	GSM437111	Cancer	Avaliable	NA	NA	NA	NA
468	GSE17538	GSM437123	Cancer	NA	NA	NA	NA	NA
469	GSE17538	GSM437127	Cancer	Avaliable	NA	NA	NA	NA
470	GSE17538	GSM437145	Cancer	Avaliable	NA	NA	NA	NA
471	GSE17538	GSM437153	Cancer	Avaliable	NA	NA	NA	NA
472	GSE17538	GSM437165	Cancer	NA	NA	NA	NA	NA
473	GSE17538	GSM437270	Cancer	Avaliable	NA	NA	NA	NA
474	GSE17538	GSM437271	Cancer	Avaliable	NA	NA	NA	NA
475	GSE17538	GSM437272	Cancer	Avaliable	NA	NA	NA	NA
476	GSE17538	GSM437273	Cancer	Avaliable	NA	NA	NA	NA
477	GSE17538	GSM437274	Cancer	Avaliable	NA	NA	NA	NA
478	GSE17538	GSM437275	Cancer	Avaliable	NA	NA	NA	NA
479	GSE17538	GSM437276	Cancer	Avaliable	NA	NA	NA	NA
480	GSE17538	GSM437277	Cancer	Avaliable	NA	NA	NA	NA
481	GSE17538	GSM437278	Cancer	Avaliable	NA	NA	NA	NA
482	GSE17538	GSM437279	Cancer	Avaliable	NA	NA	NA	NA
483	GSE17538	GSM437280	Cancer	Avaliable	NA	NA	NA	NA
484	GSE17538	GSM437281	Cancer	Avaliable	NA	NA	NA	NA
485	GSE17538	GSM437282	Cancer	Avaliable	NA	NA	NA	NA
486	GSE17538	GSM437283	Cancer	Avaliable	NA	NA	NA	NA
487	GSE17538	GSM437284	Cancer	Avaliable	NA	NA	NA	NA
488	GSE17538	GSM437285	Cancer	Avaliable	NA	NA	NA	NA
489	GSE17538	GSM437287	Cancer	Avaliable	NA	NA	NA	NA
490	GSE17538	GSM437288	Cancer	Avaliable	NA	NA	NA	NA
491	GSE17538	GSM437289	Cancer	Avaliable	NA	NA	NA	NA
492	GSE17538	GSM437290	Cancer	NA	NA	NA	NA	NA

493	GSE17538	GSM437291	Cancer	Avaliable	NA	NA	NA	NA
494	GSE17538	GSM437292	Cancer	NA	NA	NA	NA	NA
495	GSE17538	GSM437293	Cancer	NA	NA	NA	NA	NA
496	GSE17538	GSM437294	Cancer	Avaliable	NA	NA	NA	NA
497	GSE17538	GSM437295	Cancer	NA	NA	NA	NA	NA
498	GSE17538	GSM437296	Cancer	Avaliable	NA	NA	NA	NA
499	GSE17538	GSM437297	Cancer	Avaliable	NA	NA	NA	NA
500	GSE17538	GSM437298	Cancer	Avaliable	NA	NA	NA	NA
501	GSE17538	GSM437299	Cancer	Avaliable	NA	NA	NA	NA
502	GSE17538	GSM437300	Cancer	Avaliable	NA	NA	NA	NA
503	GSE17538	GSM437301	Cancer	NA	NA	NA	NA	NA
504	GSE17538	GSM437302	Cancer	NA	NA	NA	NA	NA
505	GSE17538	GSM437303	Cancer	Avaliable	NA	NA	NA	NA
506	GSE17538	GSM437304	Cancer	NA	NA	NA	NA	NA
507	GSE17538	GSM437305	Cancer	NA	NA	NA	NA	NA
508	GSE17538	GSM437306	Cancer	Avaliable	NA	NA	NA	NA
509	GSE17538	GSM437308	Cancer	NA	NA	NA	NA	NA
510	GSE17538	GSM437309	Cancer	Avaliable	NA	NA	NA	NA
511	GSE17538	GSM437310	Cancer	Avaliable	NA	NA	NA	NA
512	GSE17538	GSM437311	Cancer	Avaliable	NA	NA	NA	NA
513	GSE17538	GSM437312	Cancer	Avaliable	NA	NA	NA	NA
514	GSE17538	GSM437313	Cancer	Avaliable	NA	NA	NA	NA
515	GSE17538	GSM437314	Cancer	Avaliable	NA	NA	NA	NA
516	GSE17538	GSM437317	Cancer	NA	NA	NA	NA	NA
517	GSE17538	GSM437318	Cancer	NA	NA	NA	NA	NA
518	GSE17538	GSM437319	Cancer	NA	NA	NA	NA	NA
519	GSE17538	GSM437323	Cancer	Avaliable	NA	NA	NA	NA
520	GSE17538	GSM437324	Cancer	Avaliable	NA	NA	NA	NA
521	GSE17538	GSM472192	Cancer	NA	NA	NA	NA	NA
522	GSE17538	GSM472193	Cancer	NA	NA	NA	NA	NA
523	GSE17538	GSM472194	Cancer	NA	NA	NA	NA	NA
524	GSE17538	GSM472195	Cancer	NA	NA	NA	NA	NA
525	GSE17538	GSM472196	Cancer	NA	NA	NA	NA	NA
526	GSE17538	GSM472197	Cancer	NA	NA	NA	NA	NA
527	GSE18088	GSM452148	Cancer	NA	NA	NA	NA	MSI
528	GSE18088	GSM452149	Cancer	NA	NA	NA	NA	MSS
529	GSE18088	GSM452150	Cancer	NA	NA	NA	NA	MSS
530	GSE18088	GSM452151	Cancer	NA	NA	NA	NA	MSS
531	GSE18088	GSM452152	Cancer	NA	NA	NA	NA	MSS
532	GSE18088	GSM452153	Cancer	NA	NA	NA	NA	MSI
533	GSE18088	GSM452154	Cancer	NA	NA	NA	NA	MSS
534	GSE18088	GSM452155	Cancer	NA	NA	NA	NA	MSS
535	GSE18088	GSM452156	Cancer	NA	NA	NA	NA	MSS
536	GSE18088	GSM452157	Cancer	NA	NA	NA	NA	MSI
537	GSE18088	GSM452158	Cancer	NA	NA	NA	NA	MSS

538	GSE18088	GSM452159	Cancer	NA	NA	NA	NA	MSI
539	GSE18088	GSM452160	Cancer	NA	NA	NA	NA	MSS
540	GSE18088	GSM452161	Cancer	NA	NA	NA	NA	MSS
541	GSE18088	GSM452162	Cancer	NA	NA	NA	NA	MSS
542	GSE18088	GSM452163	Cancer	NA	NA	NA	NA	MSI
543	GSE18088	GSM452164	Cancer	NA	NA	NA	NA	MSS
544	GSE18088	GSM452165	Cancer	NA	NA	NA	NA	MSS
545	GSE18088	GSM452166	Cancer	NA	NA	NA	NA	MSS
546	GSE18088	GSM452167	Cancer	NA	NA	NA	NA	MSS
547	GSE18088	GSM452168	Cancer	NA	NA	NA	NA	MSI
548	GSE18088	GSM452169	Cancer	NA	NA	NA	NA	MSI
549	GSE18088	GSM452170	Cancer	NA	NA	NA	NA	MSS
550	GSE18088	GSM452171	Cancer	NA	NA	NA	NA	MSS
551	GSE18088	GSM452172	Cancer	NA	NA	NA	NA	MSS
552	GSE18088	GSM452173	Cancer	NA	NA	NA	NA	MSI
553	GSE18088	GSM452174	Cancer	NA	NA	NA	NA	MSI
554	GSE18088	GSM452175	Cancer	NA	NA	NA	NA	MSI
555	GSE18088	GSM452176	Cancer	NA	NA	NA	NA	MSS
556	GSE18088	GSM452177	Cancer	NA	NA	NA	NA	MSS
557	GSE18088	GSM452178	Cancer	NA	NA	NA	NA	MSS
558	GSE18088	GSM452179	Cancer	NA	NA	NA	NA	MSI
559	GSE18088	GSM452180	Cancer	NA	NA	NA	NA	MSI
560	GSE18088	GSM452181	Cancer	NA	NA	NA	NA	MSS
561	GSE18088	GSM452182	Cancer	NA	NA	NA	NA	MSI
562	GSE18088	GSM452183	Cancer	NA	NA	NA	NA	MSI
563	GSE18088	GSM452184	Cancer	NA	NA	NA	NA	MSI
564	GSE18088	GSM452185	Cancer	NA	NA	NA	NA	MSS
565	GSE18088	GSM452186	Cancer	NA	NA	NA	NA	MSS
566	GSE18088	GSM452187	Cancer	NA	NA	NA	NA	MSS
567	GSE18088	GSM452188	Cancer	NA	NA	NA	NA	MSI
568	GSE18088	GSM452189	Cancer	NA	NA	NA	NA	MSI
569	GSE18088	GSM452191	Cancer	NA	NA	NA	NA	MSS
570	GSE18088	GSM452192	Cancer	NA	NA	NA	NA	MSS
571	GSE18088	GSM452193	Cancer	NA	NA	NA	NA	MSS
572	GSE18088	GSM452194	Cancer	NA	NA	NA	NA	MSS
573	GSE18088	GSM452195	Cancer	NA	NA	NA	NA	MSS
574	GSE18088	GSM452196	Cancer	NA	NA	NA	NA	MSS
575	GSE18088	GSM452197	Cancer	NA	NA	NA	NA	MSI
576	GSE18088	GSM452198	Cancer	NA	NA	NA	NA	MSS
577	GSE18088	GSM452199	Cancer	NA	NA	NA	NA	MSS
578	GSE18088	GSM452200	Cancer	NA	NA	NA	NA	MSI
579	GSE18105	GSM452646	Cancer	NA	NA	NA	NA	NA
580	GSE18105	GSM452647	Cancer	NA	NA	NA	NA	NA
581	GSE18105	GSM452648	Cancer	NA	NA	NA	NA	NA
582	GSE18105	GSM452649	Cancer	NA	NA	NA	NA	NA

583	GSE18105	GSM452650	Cancer	NA	NA	NA	NA	NA
584	GSE18105	GSM452651	Cancer	NA	NA	NA	NA	NA
585	GSE18105	GSM452652	Cancer	NA	NA	NA	NA	NA
586	GSE18105	GSM452653	Cancer	NA	NA	NA	NA	NA
587	GSE18105	GSM452654	Cancer	NA	NA	NA	NA	NA
588	GSE18105	GSM452655	Cancer	NA	NA	NA	NA	NA
589	GSE18105	GSM452656	Cancer	NA	NA	NA	NA	NA
590	GSE18105	GSM452657	Cancer	NA	NA	NA	NA	NA
591	GSE18105	GSM452658	Cancer	NA	NA	NA	NA	NA
592	GSE18105	GSM452659	Cancer	NA	NA	NA	NA	NA
593	GSE18105	GSM452660	Cancer	NA	NA	NA	NA	NA
594	GSE18105	GSM452661	Cancer	NA	NA	NA	NA	NA
595	GSE18105	GSM452662	Cancer	NA	NA	NA	NA	NA
596	GSE18462	GSM459863	Cancer	NA	NA	NA	NA	NA
597	GSE19860	GSM496015	Cancer	NA	NA	NA	NA	MSI
598	GSE19860	GSM496016	Cancer	NA	NA	NA	NA	MSI
599	GSE19860	GSM496017	Cancer	NA	NA	NA	NA	MSI
600	GSE19860	GSM496019	Cancer	NA	NA	NA	NA	NA
601	GSE19860	GSM496020	Cancer	NA	NA	NA	NA	MSS
602	GSE19860	GSM496021	Cancer	NA	NA	NA	NA	NA
603	GSE19860	GSM496022	Cancer	NA	NA	NA	NA	NA
604	GSE19860	GSM496023	Cancer	NA	NA	NA	NA	NA
605	GSE19860	GSM496024	Cancer	NA	NA	NA	NA	MSI
606	GSE19860	GSM496025	Cancer	NA	NA	NA	NA	NA
607	GSE19860	GSM496026	Cancer	NA	NA	NA	NA	NA
608	GSE19860	GSM496027	Cancer	NA	NA	NA	NA	NA
609	GSE19860	GSM496028	Cancer	NA	NA	NA	NA	NA
610	GSE19860	GSM496029	Cancer	NA	NA	NA	NA	NA
611	GSE19860	GSM496030	Cancer	NA	NA	NA	NA	NA
612	GSE19860	GSM496031	Cancer	NA	NA	NA	NA	NA
613	GSE19860	GSM496032	Cancer	NA	NA	NA	NA	MSI
614	GSE19860	GSM496033	Cancer	NA	NA	NA	NA	NA
615	GSE19860	GSM496034	Cancer	NA	NA	NA	NA	MSI
616	GSE19860	GSM496035	Cancer	NA	NA	NA	NA	MSS
617	GSE19860	GSM496036	Cancer	NA	NA	NA	NA	NA
618	GSE19860	GSM496037	Cancer	NA	NA	NA	NA	MSI
619	GSE19860	GSM496038	Cancer	NA	NA	NA	NA	MSI
620	GSE19860	GSM496039	Cancer	NA	NA	NA	NA	NA
621	GSE19860	GSM496040	Cancer	NA	NA	NA	NA	NA
622	GSE19860	GSM496041	Cancer	NA	NA	NA	NA	NA
623	GSE19860	GSM496042	Cancer	NA	NA	NA	NA	NA
624	GSE19860	GSM496043	Cancer	NA	NA	NA	NA	NA
625	GSE19862	GSM496059	Cancer	NA	NA	NA	NA	NA
626	GSE19862	GSM496060	Cancer	NA	NA	NA	NA	MSI
627	GSE19862	GSM496061	Cancer	NA	NA	NA	NA	MSI

628	GSE19862	GSM496062	Cancer	NA	NA	NA	NA	NA
629	GSE19862	GSM496063	Cancer	NA	NA	NA	NA	NA
630	GSE19862	GSM496064	Cancer	NA	NA	NA	NA	NA
631	GSE19862	GSM496065	Cancer	NA	NA	NA	NA	MSI
632	GSE19862	GSM496066	Cancer	NA	NA	NA	NA	NA
633	GSE19862	GSM496068	Cancer	NA	NA	NA	NA	NA
634	GSE19862	GSM496069	Cancer	NA	NA	NA	NA	NA
635	GSE19862	GSM496070	Cancer	NA	NA	NA	NA	MSI
636	GSE19862	GSM496072	Cancer	NA	NA	NA	NA	MSI
637	GSE19862	GSM496074	Cancer	NA	NA	NA	NA	NA
638	GSE19862	GSM496075	Cancer	NA	NA	NA	NA	NA
639	GSE20916	GSM523283	Cancer	NA	NA	NA	NA	NA
640	GSE20916	GSM523284	Cancer	NA	NA	NA	NA	NA
641	GSE20916	GSM523285	Cancer	NA	NA	NA	NA	NA
642	GSE20916	GSM523287	Cancer	NA	NA	NA	NA	NA
643	GSE20916	GSM523288	Cancer	NA	NA	NA	NA	NA
644	GSE20916	GSM523292	Cancer	NA	NA	NA	NA	NA
645	GSE20916	GSM523293	Cancer	NA	NA	NA	NA	NA
646	GSE20916	GSM523294	Cancer	NA	NA	NA	NA	NA
647	GSE20916	GSM523295	Cancer	NA	NA	NA	NA	NA
648	GSE20916	GSM523296	Cancer	NA	NA	NA	NA	NA
649	GSE20916	GSM523298	Cancer	NA	NA	NA	NA	NA
650	GSE20916	GSM523300	Cancer	NA	NA	NA	NA	NA
651	GSE20916	GSM523301	Cancer	NA	NA	NA	NA	NA
652	GSE20916	GSM523302	Cancer	NA	NA	NA	NA	NA
653	GSE20916	GSM523303	Cancer	NA	NA	NA	NA	NA
654	GSE20916	GSM523305	Cancer	NA	NA	NA	NA	NA
655	GSE20916	GSM523306	Cancer	NA	NA	NA	NA	NA
656	GSE20916	GSM523307	Cancer	NA	NA	NA	NA	NA
657	GSE20916	GSM523308	Cancer	NA	NA	NA	NA	NA
658	GSE20916	GSM523309	Cancer	NA	NA	NA	NA	NA
659	GSE20916	GSM523312	Cancer	NA	NA	NA	NA	NA
660	GSE20916	GSM523313	Cancer	NA	NA	NA	NA	NA
661	GSE20916	GSM523315	Cancer	NA	NA	NA	NA	NA
662	GSE20916	GSM523316	Cancer	NA	NA	NA	NA	NA
663	GSE20916	GSM523317	Cancer	NA	NA	NA	NA	NA
664	GSE20916	GSM523318	Cancer	NA	NA	NA	NA	NA
665	GSE20916	GSM523319	Cancer	NA	NA	NA	NA	NA
666	GSE20916	GSM523320	Cancer	NA	NA	NA	NA	NA
667	GSE20916	GSM523321	Cancer	NA	NA	NA	NA	NA
668	GSE20916	GSM523322	Cancer	NA	NA	NA	NA	NA
669	GSE20916	GSM523323	Cancer	NA	NA	NA	NA	NA
670	GSE20916	GSM523325	Cancer	NA	NA	NA	NA	NA
671	GSE20916	GSM523326	Cancer	NA	NA	NA	NA	NA
672	GSE20916	GSM523327	Cancer	NA	NA	NA	NA	NA

673	GSE20916	GSM523328	Cancer	NA	NA	NA	NA	NA
674	GSE20916	GSM523329	Cancer	NA	NA	NA	NA	NA
675	GSE20916	GSM523331	Cancer	NA	NA	NA	NA	NA
676	GSE20916	GSM523332	Cancer	NA	NA	NA	NA	NA
677	GSE20916	GSM523333	Cancer	NA	NA	NA	NA	NA
678	GSE20916	GSM523334	Cancer	NA	NA	NA	NA	NA
679	GSE20916	GSM523335	Cancer	NA	NA	NA	NA	NA
680	GSE20916	GSM523336	Cancer	NA	NA	NA	NA	NA
681	GSE20916	GSM523337	Cancer	NA	NA	NA	NA	NA
682	GSE20916	GSM523340	Cancer	NA	NA	NA	NA	NA
683	GSE20916	GSM523341	Cancer	NA	NA	NA	NA	NA
684	GSE20916	GSM523342	Cancer	NA	NA	NA	NA	NA
685	GSE20916	GSM523344	Cancer	NA	NA	NA	NA	NA
686	GSE20916	GSM523345	Cancer	NA	NA	NA	NA	NA
687	GSE20916	GSM523346	Cancer	NA	NA	NA	NA	NA
688	GSE20916	GSM523347	Cancer	NA	NA	NA	NA	NA
689	GSE20916	GSM523348	Cancer	NA	NA	NA	NA	NA
690	GSE20916	GSM523350	Cancer	NA	NA	NA	NA	NA
691	GSE20916	GSM523351	Cancer	NA	NA	NA	NA	NA
692	GSE20916	GSM523352	Cancer	NA	NA	NA	NA	NA
693	GSE20916	GSM523353	Cancer	NA	NA	NA	NA	NA
694	GSE20916	GSM523354	Cancer	NA	NA	NA	NA	NA
695	GSE20916	GSM523355	Cancer	NA	NA	NA	NA	NA
696	GSE20916	GSM523356	Cancer	NA	NA	NA	NA	NA
697	GSE20916	GSM523357	Cancer	NA	NA	NA	NA	NA
698	GSE20916	GSM523359	Cancer	NA	NA	NA	NA	NA
699	GSE20916	GSM523361	Cancer	NA	NA	NA	NA	NA
700	GSE20916	GSM523362	Cancer	NA	NA	NA	NA	NA
701	GSE20916	GSM523363	Cancer	NA	NA	NA	NA	NA
702	GSE20916	GSM523366	Cancer	NA	NA	NA	NA	NA
703	GSE20916	GSM523367	Cancer	NA	NA	NA	NA	NA
704	GSE20916	GSM523368	Cancer	NA	NA	NA	NA	NA
705	GSE20916	GSM523370	Cancer	NA	NA	NA	NA	NA
706	GSE20916	GSM523371	Cancer	NA	NA	NA	NA	NA
707	GSE20916	GSM523372	Cancer	NA	NA	NA	NA	NA
708	GSE20916	GSM523374	Cancer	NA	NA	NA	NA	NA
709	GSE20916	GSM523375	Cancer	NA	NA	NA	NA	NA
710	GSE20916	GSM523376	Cancer	NA	NA	NA	NA	NA
711	GSE20916	GSM523378	Cancer	NA	NA	NA	NA	NA
712	GSE20916	GSM523379	Cancer	NA	NA	NA	NA	NA
713	GSE20916	GSM523380	Cancer	NA	NA	NA	NA	NA
714	GSE20916	GSM523381	Cancer	NA	NA	NA	NA	NA
715	GSE20916	GSM523383	Cancer	NA	NA	NA	NA	NA
716	GSE20916	GSM523384	Cancer	NA	NA	NA	NA	NA
717	GSE20916	GSM523385	Cancer	NA	NA	NA	NA	NA

718	GSE20916	GSM523386	Cancer	NA	NA	NA	NA	NA
719	GSE2109	GSM102429	Cancer	NA	NA	NA	NA	NA
720	GSE2109	GSM102431	Cancer	NA	NA	NA	NA	NA
721	GSE2109	GSM102442	Cancer	NA	NA	NA	NA	NA
722	GSE2109	GSM102453	Cancer	NA	NA	NA	NA	NA
723	GSE2109	GSM102460	Cancer	NA	NA	NA	NA	NA
724	GSE2109	GSM102472	Cancer	NA	NA	NA	NA	NA
725	GSE2109	GSM102485	Cancer	NA	NA	NA	NA	NA
726	GSE2109	GSM102486	Cancer	NA	NA	NA	NA	NA
727	GSE2109	GSM102497	Cancer	NA	NA	NA	NA	NA
728	GSE2109	GSM102501	Cancer	NA	NA	NA	NA	NA
729	GSE2109	GSM102513	Cancer	NA	NA	NA	NA	NA
730	GSE2109	GSM102516	Cancer	NA	NA	NA	NA	NA
731	GSE2109	GSM102518	Cancer	NA	NA	NA	NA	NA
732	GSE2109	GSM102519	Cancer	NA	NA	NA	NA	NA
733	GSE2109	GSM102521	Cancer	NA	NA	NA	NA	NA
734	GSE2109	GSM102524	Cancer	NA	NA	NA	NA	NA
735	GSE2109	GSM102533	Cancer	NA	NA	NA	NA	NA
736	GSE2109	GSM102540	Cancer	NA	NA	NA	NA	NA
737	GSE2109	GSM102549	Cancer	NA	NA	NA	NA	NA
738	GSE2109	GSM102551	Cancer	NA	NA	NA	NA	NA
739	GSE2109	GSM102559	Cancer	NA	NA	NA	NA	NA
740	GSE2109	GSM102561	Cancer	NA	NA	NA	NA	NA
741	GSE2109	GSM102568	Cancer	NA	NA	NA	NA	NA
742	GSE2109	GSM102572	Cancer	NA	NA	NA	NA	NA
743	GSE2109	GSM102577	Cancer	NA	NA	NA	NA	NA
744	GSE2109	GSM102579	Cancer	NA	NA	NA	NA	NA
745	GSE2109	GSM102581	Cancer	NA	NA	NA	NA	NA
746	GSE2109	GSM117577	Cancer	NA	NA	NA	NA	NA
747	GSE2109	GSM117635	Cancer	NA	NA	NA	NA	NA
748	GSE2109	GSM117642	Cancer	NA	NA	NA	NA	NA
749	GSE2109	GSM117656	Cancer	NA	NA	NA	NA	NA
750	GSE2109	GSM117662	Cancer	NA	NA	NA	NA	NA
751	GSE2109	GSM117664	Cancer	NA	NA	NA	NA	NA
752	GSE2109	GSM117672	Cancer	NA	NA	NA	NA	NA
753	GSE2109	GSM117673	Cancer	NA	NA	NA	NA	NA
754	GSE2109	GSM117676	Cancer	NA	NA	NA	NA	NA
755	GSE2109	GSM117678	Cancer	NA	NA	NA	NA	NA
756	GSE2109	GSM117683	Cancer	NA	NA	NA	NA	NA
757	GSE2109	GSM117707	Cancer	NA	NA	NA	NA	NA
758	GSE2109	GSM117709	Cancer	NA	NA	NA	NA	NA
759	GSE2109	GSM117720	Cancer	NA	NA	NA	NA	NA
760	GSE2109	GSM117728	Cancer	NA	NA	NA	NA	NA
761	GSE2109	GSM117738	Cancer	NA	NA	NA	NA	NA
762	GSE2109	GSM117742	Cancer	NA	NA	NA	NA	NA

763	GSE2109	GSM117746	Cancer	NA	NA	NA	NA	NA
764	GSE2109	GSM117747	Cancer	NA	NA	NA	NA	NA
765	GSE2109	GSM117752	Cancer	NA	NA	NA	NA	NA
766	GSE2109	GSM117760	Cancer	NA	NA	NA	NA	NA
767	GSE2109	GSM117775	Cancer	NA	NA	NA	NA	NA
768	GSE2109	GSM137922	Cancer	NA	NA	NA	NA	NA
769	GSE2109	GSM137926	Cancer	NA	NA	NA	NA	NA
770	GSE2109	GSM137929	Cancer	NA	NA	NA	NA	NA
771	GSE2109	GSM137942	Cancer	NA	NA	NA	NA	NA
772	GSE2109	GSM137947	Cancer	NA	NA	NA	NA	NA
773	GSE2109	GSM137949	Cancer	NA	NA	NA	NA	NA
774	GSE2109	GSM137964	Cancer	NA	NA	NA	NA	NA
775	GSE2109	GSM137967	Cancer	NA	NA	NA	NA	NA
776	GSE2109	GSM137985	Cancer	NA	NA	NA	NA	NA
777	GSE2109	GSM137993	Cancer	NA	NA	NA	NA	NA
778	GSE2109	GSM137998	Cancer	NA	NA	NA	NA	NA
779	GSE2109	GSM137999	Cancer	NA	NA	NA	NA	NA
780	GSE2109	GSM138018	Cancer	NA	NA	NA	NA	NA
781	GSE2109	GSM138022	Cancer	NA	NA	NA	NA	NA
782	GSE2109	GSM138032	Cancer	NA	NA	NA	NA	NA
783	GSE2109	GSM138036	Cancer	NA	NA	NA	NA	NA
784	GSE2109	GSM138037	Cancer	NA	NA	NA	NA	NA
785	GSE2109	GSM138042	Cancer	NA	NA	NA	NA	NA
786	GSE2109	GSM138044	Cancer	NA	NA	NA	NA	NA
787	GSE2109	GSM138048	Cancer	NA	NA	NA	NA	NA
788	GSE2109	GSM138050	Cancer	NA	NA	NA	NA	NA
789	GSE2109	GSM138052	Cancer	NA	NA	NA	NA	NA
790	GSE2109	GSM152573	Cancer	NA	NA	NA	NA	NA
791	GSE2109	GSM152579	Cancer	NA	NA	NA	NA	NA
792	GSE2109	GSM152582	Cancer	NA	NA	NA	NA	NA
793	GSE2109	GSM152591	Cancer	NA	NA	NA	NA	NA
794	GSE2109	GSM152602	Cancer	NA	NA	NA	NA	NA
795	GSE2109	GSM152610	Cancer	NA	NA	NA	NA	NA
796	GSE2109	GSM152613	Cancer	NA	NA	NA	NA	NA
797	GSE2109	GSM152614	Cancer	NA	NA	NA	NA	NA
798	GSE2109	GSM152630	Cancer	NA	NA	NA	NA	NA
799	GSE2109	GSM152632	Cancer	NA	NA	NA	NA	NA
800	GSE2109	GSM152638	Cancer	NA	NA	NA	NA	NA
801	GSE2109	GSM152658	Cancer	NA	NA	NA	NA	NA
802	GSE2109	GSM152664	Cancer	NA	NA	NA	NA	NA
803	GSE2109	GSM152666	Cancer	NA	NA	NA	NA	NA
804	GSE2109	GSM152675	Cancer	NA	NA	NA	NA	NA
805	GSE2109	GSM152684	Cancer	NA	NA	NA	NA	NA
806	GSE2109	GSM152692	Cancer	NA	NA	NA	NA	NA
807	GSE2109	GSM152695	Cancer	NA	NA	NA	NA	NA

808	GSE2109	GSM152712	Cancer	NA	NA	NA	NA	NA
809	GSE2109	GSM152714	Cancer	NA	NA	NA	NA	NA
810	GSE2109	GSM152720	Cancer	NA	NA	NA	NA	NA
811	GSE2109	GSM152725	Cancer	NA	NA	NA	NA	NA
812	GSE2109	GSM152730	Cancer	NA	NA	NA	NA	NA
813	GSE2109	GSM152762	Cancer	NA	NA	NA	NA	NA
814	GSE2109	GSM152780	Cancer	NA	NA	NA	NA	NA
815	GSE2109	GSM152790	Cancer	NA	NA	NA	NA	NA
816	GSE2109	GSM152799	Cancer	NA	NA	NA	NA	NA
817	GSE2109	GSM179778	Cancer	NA	NA	NA	NA	NA
818	GSE2109	GSM179782	Cancer	NA	NA	NA	NA	NA
819	GSE2109	GSM179793	Cancer	NA	NA	NA	NA	NA
820	GSE2109	GSM179795	Cancer	NA	NA	NA	NA	NA
821	GSE2109	GSM179803	Cancer	NA	NA	NA	NA	NA
822	GSE2109	GSM179804	Cancer	NA	NA	NA	NA	NA
823	GSE2109	GSM179805	Cancer	NA	NA	NA	NA	NA
824	GSE2109	GSM179820	Cancer	NA	NA	NA	NA	NA
825	GSE2109	GSM179831	Cancer	NA	NA	NA	NA	NA
826	GSE2109	GSM179833	Cancer	NA	NA	NA	NA	NA
827	GSE2109	GSM179838	Cancer	NA	NA	NA	NA	NA
828	GSE2109	GSM179839	Cancer	NA	NA	NA	NA	NA
829	GSE2109	GSM179844	Cancer	NA	NA	NA	NA	NA
830	GSE2109	GSM179845	Cancer	NA	NA	NA	NA	NA
831	GSE2109	GSM179858	Cancer	NA	NA	NA	NA	NA
832	GSE2109	GSM179859	Cancer	NA	NA	NA	NA	NA
833	GSE2109	GSM179860	Cancer	NA	NA	NA	NA	NA
834	GSE2109	GSM179867	Cancer	NA	NA	NA	NA	NA
835	GSE2109	GSM179868	Cancer	NA	NA	NA	NA	NA
836	GSE2109	GSM179880	Cancer	NA	NA	NA	NA	NA
837	GSE2109	GSM179882	Cancer	NA	NA	NA	NA	NA
838	GSE2109	GSM179887	Cancer	NA	NA	NA	NA	NA
839	GSE2109	GSM179888	Cancer	NA	NA	NA	NA	NA
840	GSE2109	GSM179889	Cancer	NA	NA	NA	NA	NA
841	GSE2109	GSM179897	Cancer	NA	NA	NA	NA	NA
842	GSE2109	GSM179899	Cancer	NA	NA	NA	NA	NA
843	GSE2109	GSM179908	Cancer	NA	NA	NA	NA	NA
844	GSE2109	GSM179920	Cancer	NA	NA	NA	NA	NA
845	GSE2109	GSM179922	Cancer	NA	NA	NA	NA	NA
846	GSE2109	GSM179924	Cancer	NA	NA	NA	NA	NA
847	GSE2109	GSM179925	Cancer	NA	NA	NA	NA	NA
848	GSE2109	GSM179928	Cancer	NA	NA	NA	NA	NA
849	GSE2109	GSM179930	Cancer	NA	NA	NA	NA	NA
850	GSE2109	GSM179937	Cancer	NA	NA	NA	NA	NA
851	GSE2109	GSM179948	Cancer	NA	NA	NA	NA	NA
852	GSE2109	GSM203627	Cancer	NA	NA	NA	NA	NA

853	GSE2109	GSM203640	Cancer	NA	NA	NA	NA	NA
854	GSE2109	GSM203642	Cancer	NA	NA	NA	NA	NA
855	GSE2109	GSM203645	Cancer	NA	NA	NA	NA	NA
856	GSE2109	GSM203647	Cancer	NA	NA	NA	NA	NA
857	GSE2109	GSM203653	Cancer	NA	NA	NA	NA	NA
858	GSE2109	GSM203657	Cancer	NA	NA	NA	NA	NA
859	GSE2109	GSM203667	Cancer	NA	NA	NA	NA	NA
860	GSE2109	GSM203670	Cancer	NA	NA	NA	NA	NA
861	GSE2109	GSM203674	Cancer	NA	NA	NA	NA	NA
862	GSE2109	GSM203684	Cancer	NA	NA	NA	NA	NA
863	GSE2109	GSM203687	Cancer	NA	NA	NA	NA	NA
864	GSE2109	GSM203688	Cancer	NA	NA	NA	NA	NA
865	GSE2109	GSM203691	Cancer	NA	NA	NA	NA	NA
866	GSE2109	GSM203700	Cancer	NA	NA	NA	NA	NA
867	GSE2109	GSM203702	Cancer	NA	NA	NA	NA	NA
868	GSE2109	GSM203723	Cancer	NA	NA	NA	NA	NA
869	GSE2109	GSM203728	Cancer	NA	NA	NA	NA	NA
870	GSE2109	GSM203731	Cancer	NA	NA	NA	NA	NA
871	GSE2109	GSM203733	Cancer	NA	NA	NA	NA	NA
872	GSE2109	GSM203743	Cancer	NA	NA	NA	NA	NA
873	GSE2109	GSM203755	Cancer	NA	NA	NA	NA	NA
874	GSE2109	GSM203763	Cancer	NA	NA	NA	NA	NA
875	GSE2109	GSM203782	Cancer	NA	NA	NA	NA	NA
876	GSE2109	GSM203788	Cancer	NA	NA	NA	NA	NA
877	GSE2109	GSM203800	Cancer	NA	NA	NA	NA	NA
878	GSE2109	GSM231868	Cancer	NA	NA	NA	NA	NA
879	GSE2109	GSM231875	Cancer	NA	NA	NA	NA	NA
880	GSE2109	GSM231879	Cancer	NA	NA	NA	NA	NA
881	GSE2109	GSM231904	Cancer	NA	NA	NA	NA	NA
882	GSE2109	GSM231905	Cancer	NA	NA	NA	NA	NA
883	GSE2109	GSM231915	Cancer	NA	NA	NA	NA	NA
884	GSE2109	GSM231928	Cancer	NA	NA	NA	NA	NA
885	GSE2109	GSM231934	Cancer	NA	NA	NA	NA	NA
886	GSE2109	GSM231939	Cancer	NA	NA	NA	NA	NA
887	GSE2109	GSM231953	Cancer	NA	NA	NA	NA	NA
888	GSE2109	GSM231956	Cancer	NA	NA	NA	NA	NA
889	GSE2109	GSM231958	Cancer	NA	NA	NA	NA	NA
890	GSE2109	GSM231959	Cancer	NA	NA	NA	NA	NA
891	GSE2109	GSM231961	Cancer	NA	NA	NA	NA	NA
892	GSE2109	GSM231964	Cancer	NA	NA	NA	NA	NA
893	GSE2109	GSM231987	Cancer	NA	NA	NA	NA	NA
894	GSE2109	GSM231988	Cancer	NA	NA	NA	NA	NA
895	GSE2109	GSM277680	Cancer	NA	NA	NA	NA	NA
896	GSE2109	GSM277686	Cancer	NA	NA	NA	NA	NA
897	GSE2109	GSM277689	Cancer	NA	NA	NA	NA	NA

898	GSE2109	GSM277690	Cancer	NA	NA	NA	NA	NA
899	GSE2109	GSM277728	Cancer	NA	NA	NA	NA	NA
900	GSE2109	GSM277731	Cancer	NA	NA	NA	NA	NA
901	GSE2109	GSM301656	Cancer	NA	NA	NA	NA	NA
902	GSE2109	GSM301706	Cancer	NA	NA	NA	NA	NA
903	GSE2109	GSM325789	Cancer	NA	NA	NA	NA	NA
904	GSE2109	GSM325805	Cancer	NA	NA	NA	NA	NA
905	GSE2109	GSM325806	Cancer	NA	NA	NA	NA	NA
906	GSE2109	GSM325816	Cancer	NA	NA	NA	NA	NA
907	GSE2109	GSM325818	Cancer	NA	NA	NA	NA	NA
908	GSE2109	GSM325825	Cancer	NA	NA	NA	NA	NA
909	GSE2109	GSM325829	Cancer	NA	NA	NA	NA	NA
910	GSE2109	GSM325840	Cancer	NA	NA	NA	NA	NA
911	GSE2109	GSM325841	Cancer	NA	NA	NA	NA	NA
912	GSE2109	GSM325844	Cancer	NA	NA	NA	NA	NA
913	GSE2109	GSM353890	Cancer	NA	NA	NA	NA	NA
914	GSE2109	GSM353901	Cancer	NA	NA	NA	NA	NA
915	GSE2109	GSM353905	Cancer	NA	NA	NA	NA	NA
916	GSE2109	GSM353906	Cancer	NA	NA	NA	NA	NA
917	GSE2109	GSM353915	Cancer	NA	NA	NA	NA	NA
918	GSE2109	GSM353922	Cancer	NA	NA	NA	NA	NA
919	GSE2109	GSM353934	Cancer	NA	NA	NA	NA	NA
920	GSE2109	GSM353936	Cancer	NA	NA	NA	NA	NA
921	GSE2109	GSM353939	Cancer	NA	NA	NA	NA	NA
922	GSE2109	GSM38055	Cancer	NA	NA	NA	NA	NA
923	GSE2109	GSM38074	Cancer	NA	NA	NA	NA	NA
924	GSE2109	GSM38075	Cancer	NA	NA	NA	NA	NA
925	GSE2109	GSM38077	Cancer	NA	NA	NA	NA	NA
926	GSE2109	GSM38089	Cancer	NA	NA	NA	NA	NA
927	GSE2109	GSM38098	Cancer	NA	NA	NA	NA	NA
928	GSE2109	GSM38105	Cancer	NA	NA	NA	NA	NA
929	GSE2109	GSM38107	Cancer	NA	NA	NA	NA	NA
930	GSE2109	GSM46819	Cancer	NA	NA	NA	NA	NA
931	GSE2109	GSM46832	Cancer	NA	NA	NA	NA	NA
932	GSE2109	GSM46841	Cancer	NA	NA	NA	NA	NA
933	GSE2109	GSM46845	Cancer	NA	NA	NA	NA	NA
934	GSE2109	GSM46857	Cancer	NA	NA	NA	NA	NA
935	GSE2109	GSM46861	Cancer	NA	NA	NA	NA	NA
936	GSE2109	GSM46864	Cancer	NA	NA	NA	NA	NA
937	GSE2109	GSM46865	Cancer	NA	NA	NA	NA	NA
938	GSE2109	GSM46877	Cancer	NA	NA	NA	NA	NA
939	GSE2109	GSM46878	Cancer	NA	NA	NA	NA	NA
940	GSE2109	GSM46879	Cancer	NA	NA	NA	NA	NA
941	GSE2109	GSM46887	Cancer	NA	NA	NA	NA	NA
942	GSE2109	GSM46895	Cancer	NA	NA	NA	NA	NA

943	GSE2109	GSM46899	Cancer	NA	NA	NA	NA	NA
944	GSE2109	GSM46901	Cancer	NA	NA	NA	NA	NA
945	GSE2109	GSM46915	Cancer	NA	NA	NA	NA	NA
946	GSE2109	GSM46921	Cancer	NA	NA	NA	NA	NA
947	GSE2109	GSM46924	Cancer	NA	NA	NA	NA	NA
948	GSE2109	GSM46930	Cancer	NA	NA	NA	NA	NA
949	GSE2109	GSM46931	Cancer	NA	NA	NA	NA	NA
950	GSE2109	GSM46940	Cancer	NA	NA	NA	NA	NA
951	GSE2109	GSM46956	Cancer	NA	NA	NA	NA	NA
952	GSE2109	GSM53045	Cancer	NA	NA	NA	NA	NA
953	GSE2109	GSM53047	Cancer	NA	NA	NA	NA	NA
954	GSE2109	GSM53051	Cancer	NA	NA	NA	NA	NA
955	GSE2109	GSM53055	Cancer	NA	NA	NA	NA	NA
956	GSE2109	GSM53070	Cancer	NA	NA	NA	NA	NA
957	GSE2109	GSM53073	Cancer	NA	NA	NA	NA	NA
958	GSE2109	GSM53083	Cancer	NA	NA	NA	NA	NA
959	GSE2109	GSM53106	Cancer	NA	NA	NA	NA	NA
960	GSE2109	GSM53111	Cancer	NA	NA	NA	NA	NA
961	GSE2109	GSM53113	Cancer	NA	NA	NA	NA	NA
962	GSE2109	GSM53126	Cancer	NA	NA	NA	NA	NA
963	GSE2109	GSM53135	Cancer	NA	NA	NA	NA	NA
964	GSE2109	GSM53142	Cancer	NA	NA	NA	NA	NA
965	GSE2109	GSM53148	Cancer	NA	NA	NA	NA	NA
966	GSE2109	GSM53153	Cancer	NA	NA	NA	NA	NA
967	GSE2109	GSM53156	Cancer	NA	NA	NA	NA	NA
968	GSE2109	GSM53168	Cancer	NA	NA	NA	NA	NA
969	GSE2109	GSM53169	Cancer	NA	NA	NA	NA	NA
970	GSE2109	GSM53178	Cancer	NA	NA	NA	NA	NA
971	GSE2109	GSM76490	Cancer	NA	NA	NA	NA	NA
972	GSE2109	GSM76501	Cancer	NA	NA	NA	NA	NA
973	GSE2109	GSM76512	Cancer	NA	NA	NA	NA	NA
974	GSE2109	GSM76519	Cancer	NA	NA	NA	NA	NA
975	GSE2109	GSM76520	Cancer	NA	NA	NA	NA	NA
976	GSE2109	GSM76522	Cancer	NA	NA	NA	NA	NA
977	GSE2109	GSM76524	Cancer	NA	NA	NA	NA	NA
978	GSE2109	GSM76526	Cancer	NA	NA	NA	NA	NA
979	GSE2109	GSM76529	Cancer	NA	NA	NA	NA	NA
980	GSE2109	GSM76531	Cancer	NA	NA	NA	NA	NA
981	GSE2109	GSM76548	Cancer	NA	NA	NA	NA	NA
982	GSE2109	GSM76555	Cancer	NA	NA	NA	NA	NA
983	GSE2109	GSM76571	Cancer	NA	NA	NA	NA	NA
984	GSE2109	GSM76573	Cancer	NA	NA	NA	NA	NA
985	GSE2109	GSM76575	Cancer	NA	NA	NA	NA	NA
986	GSE2109	GSM76576	Cancer	NA	NA	NA	NA	NA
987	GSE2109	GSM76583	Cancer	NA	NA	NA	NA	NA

988	GSE2109	GSM76598	Cancer	NA	NA	NA	NA	NA
989	GSE2109	GSM76605	Cancer	NA	NA	NA	NA	NA
990	GSE2109	GSM76607	Cancer	NA	NA	NA	NA	NA
991	GSE2109	GSM76608	Cancer	NA	NA	NA	NA	NA
992	GSE2109	GSM76611	Cancer	NA	NA	NA	NA	NA
993	GSE2109	GSM76617	Cancer	NA	NA	NA	NA	NA
994	GSE2109	GSM76618	Cancer	NA	NA	NA	NA	NA
995	GSE2109	GSM76629	Cancer	NA	NA	NA	NA	NA
996	GSE2109	GSM76639	Cancer	NA	NA	NA	NA	NA
997	GSE2109	GSM76645	Cancer	NA	NA	NA	NA	NA
998	GSE2109	GSM88963	Cancer	NA	NA	NA	NA	NA
999	GSE2109	GSM88968	Cancer	NA	NA	NA	NA	NA
1000	GSE2109	GSM88976	Cancer	NA	NA	NA	NA	NA
1001	GSE2109	GSM88982	Cancer	NA	NA	NA	NA	NA
1002	GSE2109	GSM88994	Cancer	NA	NA	NA	NA	NA
1003	GSE2109	GSM88999	Cancer	NA	NA	NA	NA	NA
1004	GSE2109	GSM89004	Cancer	NA	NA	NA	NA	NA
1005	GSE2109	GSM89007	Cancer	NA	NA	NA	NA	NA
1006	GSE2109	GSM89013	Cancer	NA	NA	NA	NA	NA
1007	GSE2109	GSM89026	Cancer	NA	NA	NA	NA	NA
1008	GSE2109	GSM89037	Cancer	NA	NA	NA	NA	NA
1009	GSE2109	GSM89040	Cancer	NA	NA	NA	NA	NA
1010	GSE2109	GSM89044	Cancer	NA	NA	NA	NA	NA
1011	GSE2109	GSM89047	Cancer	NA	NA	NA	NA	NA
1012	GSE2109	GSM89049	Cancer	NA	NA	NA	NA	NA
1013	GSE2109	GSM89052	Cancer	NA	NA	NA	NA	NA
1014	GSE2109	GSM89053	Cancer	NA	NA	NA	NA	NA
1015	GSE2109	GSM89061	Cancer	NA	NA	NA	NA	NA
1016	GSE2109	GSM89062	Cancer	NA	NA	NA	NA	NA
1017	GSE2109	GSM89069	Cancer	NA	NA	NA	NA	NA
1018	GSE2109	GSM89074	Cancer	NA	NA	NA	NA	NA
1019	GSE2109	GSM89075	Cancer	NA	NA	NA	NA	NA
1020	GSE2109	GSM89090	Cancer	NA	NA	NA	NA	NA
1021	GSE2109	GSM89095	Cancer	NA	NA	NA	NA	NA
1022	GSE2109	GSM89096	Cancer	NA	NA	NA	NA	NA
1023	GSE2109	GSM89098	Cancer	NA	NA	NA	NA	NA
1024	GSE2109	GSM89103	Cancer	NA	NA	NA	NA	NA
1025	GSE22242	GSM552521	Cancer	NA	NA	NA	NA	NA
1026	GSE22242	GSM552523	Cancer	NA	NA	NA	NA	NA
1027	GSE22242	GSM553691	Cancer	NA	NA	NA	NA	NA
1028	GSE23878	GSM588828	Cancer	NA	NA	NA	NA	NA
1029	GSE23878	GSM588829	Cancer	NA	NA	NA	NA	NA
1030	GSE23878	GSM588830	Cancer	NA	NA	NA	NA	NA
1031	GSE23878	GSM588831	Cancer	NA	NA	NA	NA	NA
1032	GSE23878	GSM588832	Cancer	NA	NA	NA	NA	NA

1033	GSE23878	GSM588833	Cancer	NA	NA	NA	NA	NA
1034	GSE23878	GSM588834	Cancer	NA	NA	NA	NA	NA
1035	GSE23878	GSM588835	Cancer	NA	NA	NA	NA	NA
1036	GSE23878	GSM588836	Cancer	NA	NA	NA	NA	NA
1037	GSE23878	GSM588837	Cancer	NA	NA	NA	NA	NA
1038	GSE23878	GSM588838	Cancer	NA	NA	NA	NA	NA
1039	GSE23878	GSM588839	Cancer	NA	NA	NA	NA	NA
1040	GSE23878	GSM588840	Cancer	NA	NA	NA	NA	NA
1041	GSE23878	GSM588841	Cancer	NA	NA	NA	NA	NA
1042	GSE23878	GSM588842	Cancer	NA	NA	NA	NA	NA
1043	GSE23878	GSM588843	Cancer	NA	NA	NA	NA	NA
1044	GSE23878	GSM588844	Cancer	NA	NA	NA	NA	NA
1045	GSE23878	GSM588845	Cancer	NA	NA	NA	NA	NA
1046	GSE23878	GSM588846	Cancer	NA	NA	NA	NA	NA
1047	GSE23878	GSM588847	Cancer	NA	NA	NA	NA	NA
1048	GSE23878	GSM588848	Cancer	NA	NA	NA	NA	NA
1049	GSE23878	GSM588849	Cancer	NA	NA	NA	NA	NA
1050	GSE23878	GSM588850	Cancer	NA	NA	NA	NA	NA
1051	GSE23878	GSM588851	Cancer	NA	NA	NA	NA	NA
1052	GSE23878	GSM588852	Cancer	NA	NA	NA	NA	NA
1053	GSE23878	GSM588853	Cancer	NA	NA	NA	NA	NA
1054	GSE23878	GSM588854	Cancer	NA	NA	NA	NA	NA
1055	GSE23878	GSM588855	Cancer	NA	NA	NA	NA	NA
1056	GSE23878	GSM588856	Cancer	NA	NA	NA	NA	NA
1057	GSE23878	GSM588857	Cancer	NA	NA	NA	NA	NA
1058	GSE23878	GSM588858	Cancer	NA	NA	NA	NA	NA
1059	GSE23878	GSM588859	Cancer	NA	NA	NA	NA	NA
1060	GSE23878	GSM588860	Cancer	NA	NA	NA	NA	NA
1061	GSE23878	GSM588861	Cancer	NA	NA	NA	NA	NA
1062	GSE23878	GSM588862	Cancer	NA	NA	NA	NA	NA
1063	GSE26027	GSM638942	Cancer	NA	NA	NA	NA	NA
1064	GSE26027	GSM638943	Cancer	NA	NA	NA	NA	NA
1065	GSE26027	GSM638944	Cancer	NA	NA	NA	NA	NA
1066	GSE26027	GSM638945	Cancer	NA	NA	NA	NA	NA
1067	GSE26027	GSM638946	Cancer	NA	NA	NA	NA	NA
1068	GSE26027	GSM638947	Cancer	NA	NA	NA	NA	NA
1069	GSE26027	GSM638948	Cancer	NA	NA	NA	NA	NA
1070	GSE26027	GSM638949	Cancer	NA	NA	NA	NA	NA
1071	GSE26027	GSM638950	Cancer	NA	NA	NA	NA	NA
1072	GSE26027	GSM638951	Cancer	NA	NA	NA	NA	NA
1073	GSE26027	GSM638952	Cancer	NA	NA	NA	NA	NA
1074	GSE26027	GSM638953	Cancer	NA	NA	NA	NA	NA
1075	GSE26682	GSM656542	Cancer	NA	NA	NA	NA	MSS
1076	GSE26682	GSM656543	Cancer	NA	NA	NA	NA	MSS
1077	GSE26682	GSM656545	Cancer	NA	NA	NA	NA	MSI

1078	GSE26682	GSM656546	Cancer	NA	NA	NA	NA	MSS
1079	GSE26682	GSM656548	Cancer	NA	NA	NA	NA	MSS
1080	GSE26682	GSM656549	Cancer	NA	NA	NA	NA	MSS
1081	GSE26682	GSM656550	Cancer	NA	NA	NA	NA	MSS
1082	GSE26682	GSM656551	Cancer	NA	NA	NA	NA	MSS
1083	GSE26682	GSM656552	Cancer	NA	NA	NA	NA	MSS
1084	GSE26682	GSM656555	Cancer	NA	NA	NA	NA	MSS
1085	GSE26682	GSM656556	Cancer	NA	NA	NA	NA	MSS
1086	GSE26682	GSM656557	Cancer	NA	NA	NA	NA	MSS
1087	GSE26682	GSM656558	Cancer	NA	NA	NA	NA	MSS
1088	GSE26682	GSM656559	Cancer	NA	NA	NA	NA	MSI
1089	GSE26682	GSM656560	Cancer	NA	NA	NA	NA	MSS
1090	GSE26682	GSM656561	Cancer	NA	NA	NA	NA	MSS
1091	GSE26682	GSM656564	Cancer	NA	NA	NA	NA	NA
1092	GSE26682	GSM656567	Cancer	NA	NA	NA	NA	MSS
1093	GSE26682	GSM656568	Cancer	NA	NA	NA	NA	MSS
1094	GSE26682	GSM656569	Cancer	NA	NA	NA	NA	MSS
1095	GSE26682	GSM656570	Cancer	NA	NA	NA	NA	MSS
1096	GSE26682	GSM656573	Cancer	NA	NA	NA	NA	NA
1097	GSE26682	GSM656574	Cancer	NA	NA	NA	NA	MSS
1098	GSE26682	GSM656575	Cancer	NA	NA	NA	NA	NA
1099	GSE26682	GSM656580	Cancer	NA	NA	NA	NA	MSS
1100	GSE26682	GSM656582	Cancer	NA	NA	NA	NA	MSS
1101	GSE26682	GSM656583	Cancer	NA	NA	NA	NA	MSI
1102	GSE26682	GSM656584	Cancer	NA	NA	NA	NA	MSS
1103	GSE26682	GSM656585	Cancer	NA	NA	NA	NA	MSS
1104	GSE26682	GSM656587	Cancer	NA	NA	NA	NA	MSS
1105	GSE26682	GSM656590	Cancer	NA	NA	NA	NA	MSI
1106	GSE26682	GSM656592	Cancer	NA	NA	NA	NA	MSS
1107	GSE26682	GSM656595	Cancer	NA	NA	NA	NA	MSS
1108	GSE26682	GSM656596	Cancer	NA	NA	NA	NA	MSS
1109	GSE26682	GSM656601	Cancer	NA	NA	NA	NA	MSS
1110	GSE26682	GSM656602	Cancer	NA	NA	NA	NA	MSS
1111	GSE26682	GSM656603	Cancer	NA	NA	NA	NA	MSI
1112	GSE26682	GSM656607	Cancer	NA	NA	NA	NA	MSS
1113	GSE26682	GSM656609	Cancer	NA	NA	NA	NA	NA
1114	GSE26682	GSM656610	Cancer	NA	NA	NA	NA	MSS
1115	GSE26682	GSM656612	Cancer	NA	NA	NA	NA	MSI
1116	GSE26682	GSM656613	Cancer	NA	NA	NA	NA	MSS
1117	GSE26682	GSM656617	Cancer	NA	NA	NA	NA	MSS
1118	GSE26682	GSM656618	Cancer	NA	NA	NA	NA	MSS
1119	GSE26682	GSM656619	Cancer	NA	NA	NA	NA	MSS
1120	GSE26682	GSM656625	Cancer	NA	NA	NA	NA	MSS
1121	GSE26682	GSM656627	Cancer	NA	NA	NA	NA	MSS
1122	GSE26682	GSM656631	Cancer	NA	NA	NA	NA	NA

1123	GSE26682	GSM656634	Cancer	NA	NA	NA	NA	MSS
1124	GSE26682	GSM656635	Cancer	NA	NA	NA	NA	MSS
1125	GSE26682	GSM656636	Cancer	NA	NA	NA	NA	MSS
1126	GSE26682	GSM656639	Cancer	NA	NA	NA	NA	MSS
1127	GSE26682	GSM656640	Cancer	NA	NA	NA	NA	MSS
1128	GSE26682	GSM656642	Cancer	NA	NA	NA	NA	MSS
1129	GSE26682	GSM656644	Cancer	NA	NA	NA	NA	MSS
1130	GSE26682	GSM656646	Cancer	NA	NA	NA	NA	MSS
1131	GSE26682	GSM656657	Cancer	NA	NA	NA	NA	MSS
1132	GSE26682	GSM656662	Cancer	NA	NA	NA	NA	MSS
1133	GSE26682	GSM656663	Cancer	NA	NA	NA	NA	MSS
1134	GSE26682	GSM656669	Cancer	NA	NA	NA	NA	MSI
1135	GSE26682	GSM656672	Cancer	NA	NA	NA	NA	MSS
1136	GSE26682	GSM656673	Cancer	NA	NA	NA	NA	NA
1137	GSE26682	GSM656675	Cancer	NA	NA	NA	NA	MSS
1138	GSE26682	GSM656676	Cancer	NA	NA	NA	NA	MSS
1139	GSE26682	GSM656678	Cancer	NA	NA	NA	NA	MSS
1140	GSE26682	GSM656679	Cancer	NA	NA	NA	NA	MSI
1141	GSE26682	GSM656687	Cancer	NA	NA	NA	NA	MSS
1142	GSE26682	GSM656688	Cancer	NA	NA	NA	NA	NA
1143	GSE26682	GSM656690	Cancer	NA	NA	NA	NA	MSS
1144	GSE26682	GSM656691	Cancer	NA	NA	NA	NA	MSS
1145	GSE26682	GSM656692	Cancer	NA	NA	NA	NA	MSS
1146	GSE26682	GSM656693	Cancer	NA	NA	NA	NA	MSS
1147	GSE26682	GSM656694	Cancer	NA	NA	NA	NA	MSS
1148	GSE26682	GSM656695	Cancer	NA	NA	NA	NA	MSS
1149	GSE26682	GSM656696	Cancer	NA	NA	NA	NA	MSS
1150	GSE26682	GSM656698	Cancer	NA	NA	NA	NA	MSS
1151	GSE26682	GSM656699	Cancer	NA	NA	NA	NA	MSS
1152	GSE26682	GSM656700	Cancer	NA	NA	NA	NA	MSS
1153	GSE26682	GSM656701	Cancer	NA	NA	NA	NA	MSS
1154	GSE26682	GSM656702	Cancer	NA	NA	NA	NA	MSS
1155	GSE26682	GSM656703	Cancer	NA	NA	NA	NA	MSS
1156	GSE26682	GSM656704	Cancer	NA	NA	NA	NA	MSS
1157	GSE26682	GSM656705	Cancer	NA	NA	NA	NA	MSS
1158	GSE26682	GSM656706	Cancer	NA	NA	NA	NA	MSS
1159	GSE26682	GSM656707	Cancer	NA	NA	NA	NA	MSS
1160	GSE26682	GSM656710	Cancer	NA	NA	NA	NA	MSS
1161	GSE26682	GSM656711	Cancer	NA	NA	NA	NA	MSS
1162	GSE26682	GSM656714	Cancer	NA	NA	NA	NA	MSS
1163	GSE26682	GSM656715	Cancer	NA	NA	NA	NA	MSS
1164	GSE26682	GSM656720	Cancer	NA	NA	NA	NA	MSS
1165	GSE26682	GSM656721	Cancer	NA	NA	NA	NA	MSS
1166	GSE26682	GSM656726	Cancer	NA	NA	NA	NA	MSS
1167	GSE26682	GSM656731	Cancer	NA	NA	NA	NA	MSS

1168	GSE26682	GSM656732	Cancer	NA	NA	NA	NA	MSS
1169	GSE26682	GSM656735	Cancer	NA	NA	NA	NA	MSS
1170	GSE26682	GSM656736	Cancer	NA	NA	NA	NA	MSI
1171	GSE26682	GSM656737	Cancer	NA	NA	NA	NA	MSS
1172	GSE26682	GSM656739	Cancer	NA	NA	NA	NA	MSS
1173	GSE26682	GSM656742	Cancer	NA	NA	NA	NA	MSS
1174	GSE26682	GSM656749	Cancer	NA	NA	NA	NA	MSS
1175	GSE26682	GSM656762	Cancer	NA	NA	NA	NA	NA
1176	GSE26682	GSM656768	Cancer	NA	NA	NA	NA	MSS
1177	GSE26682	GSM656769	Cancer	NA	NA	NA	NA	MSS
1178	GSE26682	GSM656770	Cancer	NA	NA	NA	NA	MSS
1179	GSE26682	GSM656772	Cancer	NA	NA	NA	NA	MSS
1180	GSE26682	GSM656775	Cancer	NA	NA	NA	NA	MSS
1181	GSE26682	GSM656776	Cancer	NA	NA	NA	NA	MSS
1182	GSE26682	GSM656777	Cancer	NA	NA	NA	NA	MSS
1183	GSE26682	GSM656782	Cancer	NA	NA	NA	NA	NA
1184	GSE26682	GSM656783	Cancer	NA	NA	NA	NA	MSS
1185	GSE26682	GSM656784	Cancer	NA	NA	NA	NA	NA
1186	GSE26682	GSM656785	Cancer	NA	NA	NA	NA	MSS
1187	GSE26682	GSM656787	Cancer	NA	NA	NA	NA	MSS
1188	GSE26682	GSM656794	Cancer	NA	NA	NA	NA	MSS
1189	GSE26682	GSM656795	Cancer	NA	NA	NA	NA	MSS
1190	GSE26682	GSM656798	Cancer	NA	NA	NA	NA	NA
1191	GSE26682	GSM656800	Cancer	NA	NA	NA	NA	MSS
1192	GSE26682	GSM656802	Cancer	NA	NA	NA	NA	MSS
1193	GSE26682	GSM656803	Cancer	NA	NA	NA	NA	MSS
1194	GSE26682	GSM656804	Cancer	NA	NA	NA	NA	MSS
1195	GSE26682	GSM656806	Cancer	NA	NA	NA	NA	MSS
1196	GSE26682	GSM656809	Cancer	NA	NA	NA	NA	MSS
1197	GSE26682	GSM656811	Cancer	NA	NA	NA	NA	MSS
1198	GSE26682	GSM656816	Cancer	NA	NA	NA	NA	MSS
1199	GSE26682	GSM656819	Cancer	NA	NA	NA	NA	MSS
1200	GSE26682	GSM656820	Cancer	NA	NA	NA	NA	MSI
1201	GSE26682	GSM656822	Cancer	NA	NA	NA	NA	MSS
1202	GSE26682	GSM656824	Cancer	NA	NA	NA	NA	MSS
1203	GSE26682	GSM656825	Cancer	NA	NA	NA	NA	MSS
1204	GSE26682	GSM656827	Cancer	NA	NA	NA	NA	MSS
1205	GSE26682	GSM656830	Cancer	NA	NA	NA	NA	MSS
1206	GSE26682	GSM656831	Cancer	NA	NA	NA	NA	MSS
1207	GSE26682	GSM656832	Cancer	NA	NA	NA	NA	MSS
1208	GSE26682	GSM656833	Cancer	NA	NA	NA	NA	MSS
1209	GSE26682	GSM656834	Cancer	NA	NA	NA	NA	MSS
1210	GSE26682	GSM656836	Cancer	NA	NA	NA	NA	MSS
1211	GSE26682	GSM656837	Cancer	NA	NA	NA	NA	MSI
1212	GSE26682	GSM656838	Cancer	NA	NA	NA	NA	MSS

1213	GSE26682	GSM656839	Cancer	NA	NA	NA	NA	MSS
1214	GSE26682	GSM656840	Cancer	NA	NA	NA	NA	MSS
1215	GSE26682	GSM656841	Cancer	NA	NA	NA	NA	MSS
1216	GSE26682	GSM656842	Cancer	NA	NA	NA	NA	MSI
1217	GSE26682	GSM656843	Cancer	NA	NA	NA	NA	MSS
1218	GSE26682	GSM656844	Cancer	NA	NA	NA	NA	NA
1219	GSE26682	GSM656845	Cancer	NA	NA	NA	NA	MSS
1220	GSE26682	GSM656846	Cancer	NA	NA	NA	NA	MSS
1221	GSE26682	GSM656847	Cancer	NA	NA	NA	NA	MSS
1222	GSE26682	GSM656848	Cancer	NA	NA	NA	NA	NA
1223	GSE26682	GSM656849	Cancer	NA	NA	NA	NA	MSI
1224	GSE26682	GSM656850	Cancer	NA	NA	NA	NA	MSI
1225	GSE26682	GSM656851	Cancer	NA	NA	NA	NA	MSI
1226	GSE26682	GSM656852	Cancer	NA	NA	NA	NA	MSS
1227	GSE26682	GSM656853	Cancer	NA	NA	NA	NA	MSS
1228	GSE26682	GSM656854	Cancer	NA	NA	NA	NA	MSI
1229	GSE26682	GSM656855	Cancer	NA	NA	NA	NA	NA
1230	GSE26682	GSM656856	Cancer	NA	NA	NA	NA	MSS
1231	GSE26682	GSM656857	Cancer	NA	NA	NA	NA	MSS
1232	GSE26682	GSM656858	Cancer	NA	NA	NA	NA	MSS
1233	GSE26682	GSM656859	Cancer	NA	NA	NA	NA	MSS
1234	GSE26682	GSM656861	Cancer	NA	NA	NA	NA	MSS
1235	GSE26682	GSM656862	Cancer	NA	NA	NA	NA	MSS
1236	GSE26682	GSM656863	Cancer	NA	NA	NA	NA	MSS
1237	GSE26682	GSM656864	Cancer	NA	NA	NA	NA	MSI
1238	GSE26682	GSM656865	Cancer	NA	NA	NA	NA	MSI
1239	GSE26682	GSM656866	Cancer	NA	NA	NA	NA	NA
1240	GSE26682	GSM656867	Cancer	NA	NA	NA	NA	MSS
1241	GSE26682	GSM656868	Cancer	NA	NA	NA	NA	MSS
1242	GSE26682	GSM656869	Cancer	NA	NA	NA	NA	MSS
1243	GSE26682	GSM656870	Cancer	NA	NA	NA	NA	MSS
1244	GSE26682	GSM656871	Cancer	NA	NA	NA	NA	NA
1245	GSE26682	GSM656872	Cancer	NA	NA	NA	NA	MSS
1246	GSE27157	GSM671377	Cancer	NA	NA	NA	NA	NA
1247	GSE27157	GSM671378	Cancer	NA	NA	NA	NA	NA
1248	GSE27157	GSM671379	Cancer	NA	NA	NA	NA	NA
1249	GSE27157	GSM671380	Cancer	NA	NA	NA	NA	NA
1250	GSE27157	GSM671381	Cancer	NA	NA	NA	NA	NA
1251	GSE27157	GSM671382	Cancer	NA	NA	NA	NA	NA
1252	GSE27157	GSM671383	Cancer	NA	NA	NA	NA	NA
1253	GSE27157	GSM671384	Cancer	NA	NA	NA	NA	NA
1254	GSE27157	GSM671385	Cancer	NA	NA	NA	NA	NA
1255	GSE27157	GSM671386	Cancer	NA	NA	NA	NA	NA
1256	GSE28702	GSM710802	Cancer	NA	NA	NA	NA	NA
1257	GSE28702	GSM710803	Cancer	NA	NA	NA	NA	NA

1258	GSE28702	GSM710805	Cancer	NA	NA	NA	NA	NA
1259	GSE28702	GSM710806	Cancer	NA	NA	NA	NA	NA
1260	GSE28702	GSM710807	Cancer	NA	NA	NA	NA	NA
1261	GSE28702	GSM710808	Cancer	NA	NA	NA	NA	NA
1262	GSE28702	GSM710809	Cancer	NA	NA	NA	NA	NA
1263	GSE28702	GSM710810	Cancer	NA	NA	NA	NA	NA
1264	GSE28702	GSM710811	Cancer	NA	NA	NA	NA	NA
1265	GSE28702	GSM710812	Cancer	NA	NA	NA	NA	NA
1266	GSE28702	GSM710813	Cancer	NA	NA	NA	NA	NA
1267	GSE28702	GSM710816	Cancer	NA	NA	NA	NA	NA
1268	GSE28702	GSM710817	Cancer	NA	NA	NA	NA	NA
1269	GSE28702	GSM710821	Cancer	NA	NA	NA	NA	NA
1270	GSE28702	GSM710822	Cancer	NA	NA	NA	NA	NA
1271	GSE28702	GSM710823	Cancer	NA	NA	NA	NA	NA
1272	GSE28702	GSM710824	Cancer	NA	NA	NA	NA	NA
1273	GSE28702	GSM710825	Cancer	NA	NA	NA	NA	NA
1274	GSE28702	GSM710826	Cancer	NA	NA	NA	NA	NA
1275	GSE28702	GSM710827	Cancer	NA	NA	NA	NA	NA
1276	GSE28702	GSM710830	Cancer	NA	NA	NA	NA	NA
1277	GSE28702	GSM710831	Cancer	NA	NA	NA	NA	NA
1278	GSE28702	GSM710832	Cancer	NA	NA	NA	NA	NA
1279	GSE28702	GSM710833	Cancer	NA	NA	NA	NA	NA
1280	GSE28702	GSM710834	Cancer	NA	NA	NA	NA	NA
1281	GSE28702	GSM710835	Cancer	NA	NA	NA	NA	NA
1282	GSE28702	GSM710836	Cancer	NA	NA	NA	NA	NA
1283	GSE28702	GSM710837	Cancer	NA	NA	NA	NA	NA
1284	GSE28702	GSM710845	Cancer	NA	NA	NA	NA	NA
1285	GSE28702	GSM710862	Cancer	NA	NA	NA	NA	NA
1286	GSE28702	GSM710863	Cancer	NA	NA	NA	NA	NA
1287	GSE28702	GSM710865	Cancer	NA	NA	NA	NA	NA
1288	GSE28702	GSM710867	Cancer	NA	NA	NA	NA	NA
1289	GSE28702	GSM710869	Cancer	NA	NA	NA	NA	NA
1290	GSE28702	GSM710871	Cancer	NA	NA	NA	NA	NA
1291	GSE28702	GSM710873	Cancer	NA	NA	NA	NA	NA
1292	GSE28702	GSM710875	Cancer	NA	NA	NA	NA	NA
1293	GSE28702	GSM710877	Cancer	NA	NA	NA	NA	NA
1294	GSE28702	GSM710879	Cancer	NA	NA	NA	NA	NA
1295	GSE28702	GSM710885	Cancer	NA	NA	NA	NA	NA
1296	GSE28702	GSM710886	Cancer	NA	NA	NA	NA	NA
1297	GSE28702	GSM710888	Cancer	NA	NA	NA	NA	NA
1298	GSE28702	GSM710890	Cancer	NA	NA	NA	NA	NA
1299	GSE28702	GSM710892	Cancer	NA	NA	NA	NA	NA
1300	GSE28702	GSM710894	Cancer	NA	NA	NA	NA	NA
1301	GSE28702	GSM710896	Cancer	NA	NA	NA	NA	NA
1302	GSE28702	GSM710900	Cancer	NA	NA	NA	NA	NA

1303	GSE28702	GSM710902	Cancer	NA	NA	NA	NA	NA
1304	GSE28702	GSM710905	Cancer	NA	NA	NA	NA	NA
1305	GSE28702	GSM710906	Cancer	NA	NA	NA	NA	NA
1306	GSE28702	GSM710908	Cancer	NA	NA	NA	NA	NA
1307	GSE28702	GSM710911	Cancer	NA	NA	NA	NA	NA
1308	GSE28702	GSM710920	Cancer	NA	NA	NA	NA	NA
1309	GSE28702	GSM710922	Cancer	NA	NA	NA	NA	NA
1310	GSE28702	GSM710924	Cancer	NA	NA	NA	NA	NA
1311	GSE28702	GSM710930	Cancer	NA	NA	NA	NA	NA
1312	GSE29621	GSM734148	Cancer	NA	NA	NA	NA	MSS
1313	GSE30540	GSM757715	Cancer	NA	NA	NA	NA	MSI
1314	GSE30540	GSM757716	Cancer	NA	NA	NA	NA	MSI
1315	GSE30540	GSM757719	Cancer	NA	NA	NA	NA	MSI
1316	GSE30540	GSM757721	Cancer	NA	NA	NA	NA	MSS
1317	GSE30540	GSM757722	Cancer	NA	NA	NA	NA	MSS
1318	GSE30540	GSM757723	Cancer	NA	NA	NA	NA	MSS
1319	GSE30540	GSM757724	Cancer	NA	NA	NA	NA	MSI
1320	GSE30540	GSM757730	Cancer	NA	NA	NA	NA	MSS
1321	GSE30540	GSM757731	Cancer	NA	NA	NA	NA	MSI
1322	GSE30540	GSM757732	Cancer	NA	NA	NA	NA	MSI
1323	GSE30540	GSM757735	Cancer	NA	NA	NA	NA	MSS
1324	GSE30540	GSM757736	Cancer	NA	NA	NA	NA	MSI
1325	GSE30540	GSM757738	Cancer	NA	NA	NA	NA	MSI
1326	GSE30540	GSM757741	Cancer	NA	NA	NA	NA	MSS
1327	GSE30540	GSM757743	Cancer	NA	NA	NA	NA	MSI
1328	GSE30540	GSM757744	Cancer	NA	NA	NA	NA	MSI
1329	GSE30540	GSM757745	Cancer	NA	NA	NA	NA	MSI
1330	GSE30540	GSM757746	Cancer	NA	NA	NA	NA	MSS
1331	GSE30540	GSM757747	Cancer	NA	NA	NA	NA	MSI
1332	GSE31595	GSM782671	Cancer	Avaliable	NA	NA	NA	NA
1333	GSE31595	GSM782674	Cancer	Avaliable	NA	NA	NA	NA
1334	GSE31595	GSM784850	Cancer	Avaliable	NA	NA	NA	NA
1335	GSE31595	GSM784851	Cancer	Avaliable	NA	NA	NA	NA
1336	GSE31595	GSM784852	Cancer	Avaliable	NA	NA	NA	NA
1337	GSE31595	GSM784853	Cancer	Avaliable	NA	NA	NA	NA
1338	GSE31595	GSM784854	Cancer	Avaliable	NA	NA	NA	NA
1339	GSE31595	GSM784855	Cancer	Avaliable	NA	NA	NA	NA
1340	GSE31595	GSM784856	Cancer	Avaliable	NA	NA	NA	NA
1341	GSE31595	GSM784857	Cancer	Avaliable	NA	NA	NA	NA
1342	GSE31595	GSM784858	Cancer	Avaliable	NA	NA	NA	NA
1343	GSE31595	GSM784860	Cancer	Avaliable	NA	NA	NA	NA
1344	GSE31595	GSM784862	Cancer	Avaliable	NA	NA	NA	NA
1345	GSE31595	GSM784865	Cancer	Avaliable	NA	NA	NA	NA
1346	GSE31595	GSM784867	Cancer	Avaliable	NA	NA	NA	NA
1347	GSE31595	GSM784871	Cancer	Avaliable	NA	NA	NA	NA

1348	GSE31595	GSM784873	Cancer	Avaliable	NA	NA	NA	NA
1349	GSE31595	GSM784875	Cancer	Avaliable	NA	NA	NA	NA
1350	GSE31595	GSM784878	Cancer	Avaliable	NA	NA	NA	NA
1351	GSE31595	GSM784881	Cancer	Avaliable	NA	NA	NA	NA
1352	GSE31595	GSM784884	Cancer	Avaliable	NA	NA	NA	NA
1353	GSE31595	GSM784885	Cancer	Avaliable	NA	NA	NA	NA
1354	GSE31595	GSM784886	Cancer	Avaliable	NA	NA	NA	NA
1355	GSE31595	GSM784887	Cancer	Avaliable	NA	NA	NA	NA
1356	GSE31595	GSM784888	Cancer	Avaliable	NA	NA	NA	NA
1357	GSE31595	GSM784889	Cancer	Avaliable	NA	NA	NA	NA
1358	GSE31595	GSM784890	Cancer	Avaliable	NA	NA	NA	NA
1359	GSE31595	GSM784891	Cancer	Avaliable	NA	NA	NA	NA
1360	GSE31595	GSM784892	Cancer	Avaliable	NA	NA	NA	NA
1361	GSE31595	GSM784893	Cancer	Avaliable	NA	NA	NA	NA
1362	GSE31595	GSM784894	Cancer	Avaliable	NA	NA	NA	NA
1363	GSE31595	GSM784901	Cancer	Avaliable	NA	NA	NA	NA
1364	GSE31595	GSM784902	Cancer	Avaliable	NA	NA	NA	NA
1365	GSE31595	GSM784903	Cancer	Avaliable	NA	NA	NA	NA
1366	GSE31595	GSM784904	Cancer	Avaliable	NA	NA	NA	NA
1367	GSE31595	GSM784905	Cancer	Avaliable	NA	NA	NA	NA
1368	GSE31595	GSM784906	Cancer	Avaliable	NA	NA	NA	NA
1369	GSE33113	GSM820048	Cancer	Avaliable	NA	NA	NA	NA
1370	GSE33113	GSM820049	Cancer	Avaliable	NA	NA	NA	NA
1371	GSE33113	GSM820050	Cancer	Avaliable	NA	NA	NA	NA
1372	GSE33113	GSM820051	Cancer	Avaliable	NA	NA	NA	NA
1373	GSE33113	GSM820052	Cancer	Avaliable	NA	NA	NA	NA
1374	GSE33113	GSM820053	Cancer	Avaliable	NA	NA	NA	NA
1375	GSE33113	GSM820054	Cancer	Avaliable	NA	NA	NA	NA
1376	GSE33113	GSM820055	Cancer	Avaliable	NA	NA	NA	NA
1377	GSE33113	GSM820056	Cancer	Avaliable	NA	NA	NA	NA
1378	GSE33113	GSM820057	Cancer	Avaliable	NA	NA	NA	NA
1379	GSE33113	GSM820058	Cancer	Avaliable	NA	NA	NA	NA
1380	GSE33113	GSM820059	Cancer	Avaliable	NA	NA	NA	NA
1381	GSE33113	GSM820060	Cancer	Avaliable	NA	NA	NA	NA
1382	GSE33113	GSM820061	Cancer	Avaliable	NA	NA	NA	NA
1383	GSE33113	GSM820062	Cancer	Avaliable	NA	NA	NA	NA
1384	GSE33113	GSM820063	Cancer	Avaliable	NA	NA	NA	NA
1385	GSE33113	GSM820064	Cancer	Avaliable	NA	NA	NA	NA
1386	GSE33113	GSM820065	Cancer	Avaliable	NA	NA	NA	NA
1387	GSE33113	GSM820066	Cancer	Avaliable	NA	NA	NA	NA
1388	GSE33113	GSM820067	Cancer	Avaliable	NA	NA	NA	NA
1389	GSE33113	GSM820068	Cancer	Avaliable	NA	NA	NA	NA
1390	GSE33113	GSM820069	Cancer	Avaliable	NA	NA	NA	NA
1391	GSE33113	GSM820070	Cancer	Avaliable	NA	NA	NA	NA
1392	GSE33113	GSM820071	Cancer	Avaliable	NA	NA	NA	NA

1393	GSE33113	GSM820073	Cancer	Avaliable	NA	NA	NA	NA
1394	GSE33113	GSM820074	Cancer	Avaliable	NA	NA	NA	NA
1395	GSE33113	GSM820075	Cancer	Avaliable	NA	NA	NA	NA
1396	GSE33113	GSM820076	Cancer	Avaliable	NA	NA	NA	NA
1397	GSE33113	GSM820077	Cancer	Avaliable	NA	NA	NA	NA
1398	GSE33113	GSM820078	Cancer	Avaliable	NA	NA	NA	NA
1399	GSE33113	GSM820079	Cancer	Avaliable	NA	NA	NA	NA
1400	GSE33113	GSM820080	Cancer	Avaliable	NA	NA	NA	NA
1401	GSE33113	GSM820081	Cancer	Avaliable	NA	NA	NA	NA
1402	GSE33113	GSM820082	Cancer	Avaliable	NA	NA	NA	NA
1403	GSE33113	GSM820083	Cancer	Avaliable	NA	NA	NA	NA
1404	GSE33113	GSM820084	Cancer	Avaliable	NA	NA	NA	NA
1405	GSE33113	GSM820085	Cancer	Avaliable	NA	NA	NA	NA
1406	GSE33113	GSM820086	Cancer	Avaliable	NA	NA	NA	NA
1407	GSE33113	GSM820087	Cancer	Avaliable	NA	NA	NA	NA
1408	GSE33113	GSM820088	Cancer	Avaliable	NA	NA	NA	NA
1409	GSE33113	GSM820089	Cancer	Avaliable	NA	NA	NA	NA
1410	GSE33113	GSM820090	Cancer	Avaliable	NA	NA	NA	NA
1411	GSE33113	GSM820091	Cancer	Avaliable	NA	NA	NA	NA
1412	GSE33113	GSM820092	Cancer	Avaliable	NA	NA	NA	NA
1413	GSE33113	GSM820093	Cancer	Avaliable	NA	NA	NA	NA
1414	GSE33113	GSM820094	Cancer	Avaliable	NA	NA	NA	NA
1415	GSE33113	GSM820095	Cancer	Avaliable	NA	NA	NA	NA
1416	GSE33113	GSM820096	Cancer	Avaliable	NA	NA	NA	NA
1417	GSE33113	GSM820097	Cancer	Avaliable	NA	NA	NA	NA
1418	GSE33113	GSM820098	Cancer	Avaliable	NA	NA	NA	NA
1419	GSE33113	GSM820099	Cancer	Avaliable	NA	NA	NA	NA
1420	GSE33113	GSM820100	Cancer	Avaliable	NA	NA	NA	NA
1421	GSE33113	GSM820101	Cancer	Avaliable	NA	NA	NA	NA
1422	GSE33113	GSM820102	Cancer	Avaliable	NA	NA	NA	NA
1423	GSE33113	GSM820103	Cancer	Avaliable	NA	NA	NA	NA
1424	GSE33113	GSM820104	Cancer	Avaliable	NA	NA	NA	NA
1425	GSE33113	GSM820105	Cancer	Avaliable	NA	NA	NA	NA
1426	GSE33113	GSM820106	Cancer	Avaliable	NA	NA	NA	NA
1427	GSE33113	GSM820107	Cancer	Avaliable	NA	NA	NA	NA
1428	GSE33113	GSM820108	Cancer	Avaliable	NA	NA	NA	NA
1429	GSE33113	GSM820109	Cancer	Avaliable	NA	NA	NA	NA
1430	GSE33113	GSM820110	Cancer	Avaliable	NA	NA	NA	NA
1431	GSE33113	GSM820111	Cancer	Avaliable	NA	NA	NA	NA
1432	GSE33113	GSM820112	Cancer	Avaliable	NA	NA	NA	NA
1433	GSE33113	GSM820113	Cancer	Avaliable	NA	NA	NA	NA
1434	GSE33113	GSM820114	Cancer	Avaliable	NA	NA	NA	NA
1435	GSE33113	GSM820115	Cancer	Avaliable	NA	NA	NA	NA
1436	GSE33113	GSM820116	Cancer	Avaliable	NA	NA	NA	NA
1437	GSE33113	GSM820117	Cancer	Avaliable	NA	NA	NA	NA

1438	GSE33113	GSM820118	Cancer	Avaliable	NA	NA	NA	NA
1439	GSE33113	GSM820119	Cancer	Avaliable	NA	NA	NA	NA
1440	GSE33113	GSM820120	Cancer	Avaliable	NA	NA	NA	NA
1441	GSE33113	GSM820121	Cancer	Avaliable	NA	NA	NA	NA
1442	GSE33113	GSM820122	Cancer	Avaliable	NA	NA	NA	NA
1443	GSE33113	GSM820123	Cancer	Avaliable	NA	NA	NA	NA
1444	GSE33113	GSM820124	Cancer	Avaliable	NA	NA	NA	NA
1445	GSE33113	GSM820125	Cancer	Avaliable	NA	NA	NA	NA
1446	GSE33113	GSM820126	Cancer	Avaliable	NA	NA	NA	NA
1447	GSE33113	GSM820127	Cancer	Avaliable	NA	NA	NA	NA
1448	GSE33113	GSM820128	Cancer	Avaliable	NA	NA	NA	NA
1449	GSE33113	GSM820129	Cancer	Avaliable	NA	NA	NA	NA
1450	GSE33113	GSM820130	Cancer	Avaliable	NA	NA	NA	NA
1451	GSE33113	GSM820131	Cancer	Avaliable	NA	NA	NA	NA
1452	GSE33113	GSM820132	Cancer	Avaliable	NA	NA	NA	NA
1453	GSE33113	GSM820133	Cancer	Avaliable	NA	NA	NA	NA
1454	GSE33113	GSM820134	Cancer	Avaliable	NA	NA	NA	NA
1455	GSE33113	GSM820135	Cancer	Avaliable	NA	NA	NA	NA
1456	GSE33113	GSM820136	Cancer	Avaliable	NA	NA	NA	NA
1457	GSE33113	GSM820137	Cancer	Avaliable	NA	NA	NA	NA
1458	GSE34489	GSM850023	Cancer	NA	NA	NA	NA	MSS
1459	GSE34489	GSM850024	Cancer	NA	NA	NA	NA	MSI
1460	GSE34489	GSM850025	Cancer	NA	NA	NA	NA	MSI
1461	GSE34489	GSM850026	Cancer	NA	NA	NA	NA	MSI
1462	GSE34489	GSM850027	Cancer	NA	NA	NA	NA	MSS
1463	GSE34489	GSM850028	Cancer	NA	NA	NA	NA	MSI
1464	GSE34489	GSM850029	Cancer	NA	NA	NA	NA	MSI
1465	GSE34489	GSM850030	Cancer	NA	NA	NA	NA	MSI
1466	GSE34489	GSM850031	Cancer	NA	NA	NA	NA	MSI
1467	GSE34489	GSM850032	Cancer	NA	NA	NA	NA	MSI
1468	GSE34489	GSM850033	Cancer	NA	NA	NA	NA	MSI
1469	GSE34489	GSM850035	Cancer	NA	NA	NA	NA	MSI
1470	GSE34489	GSM850036	Cancer	NA	NA	NA	NA	MSI
1471	GSE34489	GSM850037	Cancer	NA	NA	NA	NA	MSI
1472	GSE34489	GSM850038	Cancer	NA	NA	NA	NA	MSS
1473	GSE34489	GSM850039	Cancer	NA	NA	NA	NA	MSS
1474	GSE34489	GSM850040	Cancer	NA	NA	NA	NA	MSI
1475	GSE34489	GSM850041	Cancer	NA	NA	NA	NA	MSS
1476	GSE34489	GSM850042	Cancer	NA	NA	NA	NA	MSI
1477	GSE34489	GSM850043	Cancer	NA	NA	NA	NA	MSI
1478	GSE34489	GSM850044	Cancer	NA	NA	NA	NA	MSI
1479	GSE34489	GSM850045	Cancer	NA	NA	NA	NA	MSS
1480	GSE34489	GSM850046	Cancer	NA	NA	NA	NA	MSS
1481	GSE34489	GSM850048	Cancer	NA	NA	NA	NA	MSI
1482	GSE34489	GSM850049	Cancer	NA	NA	NA	NA	MSI

1483	GSE34489	GSM850051	Cancer	NA	NA	NA	NA	MSI
1484	GSE34489	GSM850052	Cancer	NA	NA	NA	NA	MSI
1485	GSE34489	GSM850053	Cancer	NA	NA	NA	NA	MSS
1486	GSE34489	GSM850054	Cancer	NA	NA	NA	NA	MSI
1487	GSE34489	GSM850055	Cancer	NA	NA	NA	NA	MSI
1488	GSE35896	GSM877126	Cancer	NA	MU	WT	WT	MSS
1489	GSE35896	GSM877127	Cancer	NA	WT	WT	WT	MSI
1490	GSE35896	GSM877128	Cancer	NA	WT	MU	WT	MSS
1491	GSE35896	GSM877129	Cancer	NA	WT	WT	MU	MSS
1492	GSE35896	GSM877130	Cancer	NA	WT	WT	MU	MSS
1493	GSE35896	GSM877131	Cancer	NA	WT	WT	WT	MSS
1494	GSE35896	GSM877132	Cancer	NA	MU	WT	WT	MSS
1495	GSE35896	GSM877133	Cancer	NA	WT	WT	WT	MSS
1496	GSE35896	GSM877134	Cancer	NA	MU	WT	MU	MSS
1497	GSE35896	GSM877135	Cancer	NA	WT	WT	MU	MSS
1498	GSE35896	GSM877136	Cancer	NA	MU	WT	WT	MSS
1499	GSE35896	GSM877137	Cancer	NA	WT	WT	MU	MSS
1500	GSE35896	GSM877138	Cancer	NA	WT	WT	WT	MSI
1501	GSE35896	GSM877139	Cancer	NA	MU	WT	MU	MSS
1502	GSE35896	GSM877140	Cancer	NA	MU	WT	MU	MSI
1503	GSE35896	GSM877141	Cancer	NA	MU	WT	MU	MSS
1504	GSE35896	GSM877143	Cancer	NA	MU	WT	MU	MSS
1505	GSE35896	GSM877144	Cancer	NA	WT	MU	WT	MSS
1506	GSE35896	GSM877145	Cancer	NA	MU	WT	MU	MSS
1507	GSE35896	GSM877146	Cancer	NA	WT	WT	WT	MSS
1508	GSE35896	GSM877148	Cancer	NA	WT	WT	MU	MSS
1509	GSE35896	GSM877149	Cancer	NA	WT	WT	MU	MSS
1510	GSE35896	GSM877150	Cancer	NA	MU	WT	WT	NA
1511	GSE35896	GSM877151	Cancer	NA	WT	WT	MU	MSS
1512	GSE35896	GSM877152	Cancer	NA	MU	WT	WT	MSS
1513	GSE35896	GSM877153	Cancer	NA	WT	MU	WT	MSS
1514	GSE35896	GSM877154	Cancer	NA	MU	WT	WT	MSS
1515	GSE35896	GSM877155	Cancer	NA	MU	WT	WT	MSS
1516	GSE35896	GSM877156	Cancer	NA	WT	WT	WT	MSI
1517	GSE35896	GSM877157	Cancer	NA	WT	WT	MU	MSS
1518	GSE35896	GSM877158	Cancer	NA	MU	WT	MU	MSS
1519	GSE35896	GSM877159	Cancer	NA	WT	WT	WT	MSS
1520	GSE35896	GSM877160	Cancer	NA	WT	WT	MU	MSS
1521	GSE35896	GSM877161	Cancer	NA	WT	WT	WT	MSS
1522	GSE35896	GSM877162	Cancer	NA	WT	MU	WT	MSI
1523	GSE35896	GSM877163	Cancer	NA	MU	WT	WT	MSS
1524	GSE35896	GSM877164	Cancer	NA	MU	WT	WT	MSS
1525	GSE35896	GSM877165	Cancer	NA	MU	WT	MU	MSS
1526	GSE35896	GSM877166	Cancer	NA	WT	MU	WT	MSS
1527	GSE35896	GSM877167	Cancer	NA	MU	WT	WT	MSS

1528	GSE35896	GSM877168	Cancer	NA	WT	WT	MU	MSS
1529	GSE35896	GSM877169	Cancer	NA	WT	WT	WT	MSS
1530	GSE35896	GSM877170	Cancer	NA	MU	WT	MU	MSS
1531	GSE35896	GSM877171	Cancer	NA	MU	WT	WT	MSS
1532	GSE35896	GSM877173	Cancer	NA	WT	WT	WT	MSS
1533	GSE35896	GSM877174	Cancer	NA	MU	WT	MU	MSS
1534	GSE35896	GSM877175	Cancer	NA	MU	WT	MU	MSS
1535	GSE35896	GSM877176	Cancer	NA	MU	WT	WT	MSS
1536	GSE35896	GSM877177	Cancer	NA	WT	WT	MU	MSS
1537	GSE35896	GSM877178	Cancer	NA	MU	WT	WT	MSS
1538	GSE35896	GSM877179	Cancer	NA	WT	WT	WT	MSS
1539	GSE35896	GSM877180	Cancer	NA	WT	WT	MU	MSS
1540	GSE35896	GSM877181	Cancer	NA	MU	WT	WT	MSS
1541	GSE35896	GSM877182	Cancer	NA	WT	MU	MU	MSS
1542	GSE35896	GSM877183	Cancer	NA	MU	WT	WT	MSS
1543	GSE35896	GSM877184	Cancer	NA	WT	WT	MU	MSS
1544	GSE35896	GSM877185	Cancer	NA	MU	WT	WT	MSS
1545	GSE35896	GSM877186	Cancer	NA	WT	WT	MU	MSS
1546	GSE35896	GSM877187	Cancer	NA	WT	WT	WT	MSS
1547	GSE35896	GSM877188	Cancer	NA	MU	WT	WT	MSS
1548	GSE37364	GSM916716	Cancer	NA	NA	NA	NA	NA
1549	GSE37364	GSM916717	Cancer	NA	NA	NA	NA	NA
1550	GSE37364	GSM916718	Cancer	NA	NA	NA	NA	NA
1551	GSE37364	GSM916719	Cancer	NA	NA	NA	NA	NA
1552	GSE37364	GSM916720	Cancer	NA	NA	NA	NA	NA
1553	GSE37364	GSM916721	Cancer	NA	NA	NA	NA	NA
1554	GSE37364	GSM916722	Cancer	NA	NA	NA	NA	NA
1555	GSE37364	GSM916723	Cancer	NA	NA	NA	NA	NA
1556	GSE37364	GSM916724	Cancer	NA	NA	NA	NA	NA
1557	GSE37364	GSM916725	Cancer	NA	NA	NA	NA	NA
1558	GSE37364	GSM916726	Cancer	NA	NA	NA	NA	NA
1559	GSE37364	GSM916727	Cancer	NA	NA	NA	NA	NA
1560	GSE37364	GSM916728	Cancer	NA	NA	NA	NA	NA
1561	GSE37364	GSM916729	Cancer	NA	NA	NA	NA	NA
1562	GSE37364	GSM916730	Cancer	NA	NA	NA	NA	NA
1563	GSE37364	GSM916731	Cancer	NA	NA	NA	NA	NA
1564	GSE37364	GSM916732	Cancer	NA	NA	NA	NA	NA
1565	GSE37364	GSM916733	Cancer	NA	NA	NA	NA	NA
1566	GSE37364	GSM916734	Cancer	NA	NA	NA	NA	NA
1567	GSE37364	GSM916735	Cancer	NA	NA	NA	NA	NA
1568	GSE37364	GSM916736	Cancer	NA	NA	NA	NA	NA
1569	GSE37364	GSM916737	Cancer	NA	NA	NA	NA	NA
1570	GSE37364	GSM916738	Cancer	NA	NA	NA	NA	NA
1571	GSE37364	GSM916739	Cancer	NA	NA	NA	NA	NA
1572	GSE37364	GSM916740	Cancer	NA	NA	NA	NA	NA

1573	GSE37364	GSM916741	Cancer	NA	NA	NA	NA	NA
1574	GSE37364	GSM916742	Cancer	NA	NA	NA	NA	NA
1575	GSE37892	GSM929496	Cancer	Avaliable	NA	NA	NA	NA
1576	GSE37892	GSM929497	Cancer	Avaliable	NA	NA	NA	NA
1577	GSE37892	GSM929498	Cancer	Avaliable	NA	NA	NA	NA
1578	GSE37892	GSM929499	Cancer	Avaliable	NA	NA	NA	NA
1579	GSE37892	GSM929500	Cancer	Avaliable	NA	NA	NA	NA
1580	GSE37892	GSM929502	Cancer	Avaliable	NA	NA	NA	NA
1581	GSE37892	GSM929503	Cancer	Avaliable	NA	NA	NA	NA
1582	GSE37892	GSM929504	Cancer	Avaliable	NA	NA	NA	NA
1583	GSE37892	GSM929505	Cancer	Avaliable	NA	NA	NA	NA
1584	GSE37892	GSM929529	Cancer	Avaliable	NA	NA	NA	NA
1585	GSE37892	GSM929579	Cancer	Avaliable	NA	NA	NA	NA
1586	GSE37892	GSM929616	Cancer	Avaliable	NA	NA	NA	NA
1587	GSE38832	GSM950423	Cancer	NA	NA	NA	NA	NA
1588	GSE38832	GSM950424	Cancer	NA	NA	NA	NA	NA
1589	GSE38832	GSM950425	Cancer	NA	NA	NA	NA	NA
1590	GSE38832	GSM950426	Cancer	NA	NA	NA	NA	NA
1591	GSE38832	GSM950427	Cancer	NA	NA	NA	NA	NA
1592	GSE38832	GSM950428	Cancer	NA	NA	NA	NA	NA
1593	GSE38832	GSM950445	Cancer	NA	NA	NA	NA	NA
1594	GSE38832	GSM950446	Cancer	NA	NA	NA	NA	NA
1595	GSE38832	GSM950447	Cancer	NA	NA	NA	NA	NA
1596	GSE38832	GSM950448	Cancer	NA	NA	NA	NA	NA
1597	GSE38832	GSM950449	Cancer	NA	NA	NA	NA	NA
1598	GSE38832	GSM950450	Cancer	NA	NA	NA	NA	NA
1599	GSE38832	GSM950451	Cancer	NA	NA	NA	NA	NA
1600	GSE38832	GSM950452	Cancer	NA	NA	NA	NA	NA
1601	GSE38832	GSM950453	Cancer	NA	NA	NA	NA	NA
1602	GSE38832	GSM950454	Cancer	NA	NA	NA	NA	NA
1603	GSE38832	GSM950455	Cancer	NA	NA	NA	NA	NA
1604	GSE38832	GSM950456	Cancer	NA	NA	NA	NA	NA
1605	GSE38832	GSM950457	Cancer	NA	NA	NA	NA	NA
1606	GSE38832	GSM950458	Cancer	NA	NA	NA	NA	NA
1607	GSE38832	GSM950459	Cancer	NA	NA	NA	NA	NA
1608	GSE38832	GSM950460	Cancer	NA	NA	NA	NA	NA
1609	GSE38832	GSM950461	Cancer	NA	NA	NA	NA	NA
1610	GSE38832	GSM950462	Cancer	NA	NA	NA	NA	NA
1611	GSE38832	GSM950463	Cancer	NA	NA	NA	NA	NA
1612	GSE38832	GSM950465	Cancer	NA	NA	NA	NA	NA
1613	GSE38832	GSM950466	Cancer	NA	NA	NA	NA	NA
1614	GSE38832	GSM950467	Cancer	NA	NA	NA	NA	NA
1615	GSE38832	GSM950470	Cancer	NA	NA	NA	NA	NA
1616	GSE38832	GSM950471	Cancer	NA	NA	NA	NA	NA
1617	GSE38832	GSM950472	Cancer	NA	NA	NA	NA	NA

1618	GSE38832	GSM950473	Cancer	NA	NA	NA	NA	NA
1619	GSE38832	GSM950474	Cancer	NA	NA	NA	NA	NA
1620	GSE38832	GSM950475	Cancer	NA	NA	NA	NA	NA
1621	GSE38832	GSM950476	Cancer	NA	NA	NA	NA	NA
1622	GSE38832	GSM950477	Cancer	NA	NA	NA	NA	NA
1623	GSE38832	GSM950478	Cancer	NA	NA	NA	NA	NA
1624	GSE38832	GSM950479	Cancer	NA	NA	NA	NA	NA
1625	GSE38832	GSM950480	Cancer	NA	NA	NA	NA	NA
1626	GSE38832	GSM950481	Cancer	NA	NA	NA	NA	NA
1627	GSE38832	GSM950482	Cancer	NA	NA	NA	NA	NA
1628	GSE38832	GSM950483	Cancer	NA	NA	NA	NA	NA
1629	GSE38832	GSM950484	Cancer	NA	NA	NA	NA	NA
1630	GSE38832	GSM950485	Cancer	NA	NA	NA	NA	NA
1631	GSE38832	GSM950486	Cancer	NA	NA	NA	NA	NA
1632	GSE38832	GSM950487	Cancer	NA	NA	NA	NA	NA
1633	GSE38832	GSM950488	Cancer	NA	NA	NA	NA	NA
1634	GSE38832	GSM950489	Cancer	NA	NA	NA	NA	NA
1635	GSE38832	GSM950490	Cancer	NA	NA	NA	NA	NA
1636	GSE38832	GSM950491	Cancer	NA	NA	NA	NA	NA
1637	GSE38832	GSM950492	Cancer	NA	NA	NA	NA	NA
1638	GSE38832	GSM950493	Cancer	NA	NA	NA	NA	NA
1639	GSE38832	GSM950494	Cancer	NA	NA	NA	NA	NA
1640	GSE38832	GSM950495	Cancer	NA	NA	NA	NA	NA
1641	GSE38832	GSM950496	Cancer	NA	NA	NA	NA	NA
1642	GSE38832	GSM950497	Cancer	NA	NA	NA	NA	NA
1643	GSE38832	GSM950498	Cancer	NA	NA	NA	NA	NA
1644	GSE38832	GSM950499	Cancer	NA	NA	NA	NA	NA
1645	GSE38832	GSM950500	Cancer	NA	NA	NA	NA	NA
1646	GSE38832	GSM950501	Cancer	NA	NA	NA	NA	NA
1647	GSE38832	GSM950502	Cancer	NA	NA	NA	NA	NA
1648	GSE38832	GSM950503	Cancer	NA	NA	NA	NA	NA
1649	GSE38832	GSM950504	Cancer	NA	NA	NA	NA	NA
1650	GSE38832	GSM950505	Cancer	NA	NA	NA	NA	NA
1651	GSE38832	GSM950506	Cancer	NA	NA	NA	NA	NA
1652	GSE38832	GSM950507	Cancer	NA	NA	NA	NA	NA
1653	GSE39084	GSM955444	Cancer	Avaliable	MU	WT	WT	NA
1654	GSE39084	GSM955445	Cancer	NA	WT	WT	WT	NA
1655	GSE39084	GSM955449	Cancer	NA	WT	WT	WT	NA
1656	GSE39084	GSM955450	Cancer	NA	MU	WT	MU	NA
1657	GSE39084	GSM955451	Cancer	Avaliable	WT	WT	WT	NA
1658	GSE39084	GSM955456	Cancer	Avaliable	WT	WT	MU	NA
1659	GSE39084	GSM955481	Cancer	Avaliable	MU	WT	MU	NA
1660	GSE39084	GSM955485	Cancer	Avaliable	MU	WT	WT	NA
1661	GSE39084	GSM955490	Cancer	Avaliable	WT	WT	MU	NA
1662	GSE39582	GSM971957	Cancer	NA	MU	WT	MU	NA

1663	GSE39582	GSM971958	Cancer	NA	WT	WT	WT	NA
1664	GSE39582	GSM971959	Cancer	Avaliable	WT	WT	MU	NA
1665	GSE39582	GSM971960	Cancer	Avaliable	WT	WT	MU	NA
1666	GSE39582	GSM971961	Cancer	NA	WT	WT	MU	NA
1667	GSE39582	GSM971962	Cancer	Avaliable	WT	WT	WT	NA
1668	GSE39582	GSM971963	Cancer	Avaliable	WT	WT	WT	NA
1669	GSE39582	GSM971964	Cancer	Avaliable	WT	WT	MU	NA
1670	GSE39582	GSM971965	Cancer	Avaliable	MU	WT	WT	NA
1671	GSE39582	GSM971966	Cancer	Avaliable	MU	WT	MU	NA
1672	GSE39582	GSM971967	Cancer	Avaliable	MU	WT	WT	NA
1673	GSE39582	GSM971968	Cancer	Avaliable	MU	WT	MU	NA
1674	GSE39582	GSM971969	Cancer	Avaliable	WT	WT	WT	NA
1675	GSE39582	GSM971970	Cancer	Avaliable	MU	WT	WT	NA
1676	GSE39582	GSM971971	Cancer	NA	MU	WT	WT	NA
1677	GSE39582	GSM971972	Cancer	Avaliable	WT	WT	WT	NA
1678	GSE39582	GSM971973	Cancer	Avaliable	WT	WT	WT	NA
1679	GSE39582	GSM971974	Cancer	NA	MU	WT	MU	NA
1680	GSE39582	GSM971975	Cancer	Avaliable	WT	WT	WT	NA
1681	GSE39582	GSM971976	Cancer	Avaliable	WT	WT	MU	NA
1682	GSE39582	GSM971977	Cancer	Avaliable	MU	WT	WT	NA
1683	GSE39582	GSM971978	Cancer	Avaliable	MU	WT	MU	NA
1684	GSE39582	GSM971979	Cancer	Avaliable	WT	WT	WT	NA
1685	GSE39582	GSM971980	Cancer	Avaliable	WT	MU	WT	NA
1686	GSE39582	GSM971981	Cancer	Avaliable	MU	WT	WT	NA
1687	GSE39582	GSM971982	Cancer	Avaliable	MU	WT	MU	NA
1688	GSE39582	GSM971983	Cancer	Avaliable	WT	MU	MU	NA
1689	GSE39582	GSM971984	Cancer	Avaliable	WT	MU	WT	NA
1690	GSE39582	GSM971985	Cancer	Avaliable	MU	WT	WT	NA
1691	GSE39582	GSM971986	Cancer	Avaliable	WT	WT	MU	NA
1692	GSE39582	GSM971987	Cancer	Avaliable	WT	WT	WT	NA
1693	GSE39582	GSM971988	Cancer	NA	WT	MU	MU	NA
1694	GSE39582	GSM971989	Cancer	Avaliable	MU	WT	WT	NA
1695	GSE39582	GSM971990	Cancer	Avaliable	MU	WT	WT	NA
1696	GSE39582	GSM971991	Cancer	Avaliable	WT	WT	MU	NA
1697	GSE39582	GSM971992	Cancer	Avaliable	WT	WT	WT	NA
1698	GSE39582	GSM971993	Cancer	Avaliable	MU	WT	WT	NA
1699	GSE39582	GSM971994	Cancer	Avaliable	WT	WT	MU	NA
1700	GSE39582	GSM971995	Cancer	Avaliable	WT	WT	WT	NA
1701	GSE39582	GSM971996	Cancer	Avaliable	WT	WT	WT	NA
1702	GSE39582	GSM971997	Cancer	Avaliable	WT	MU	MU	NA
1703	GSE39582	GSM971998	Cancer	Avaliable	MU	WT	WT	NA
1704	GSE39582	GSM971999	Cancer	Avaliable	WT	WT	MU	NA
1705	GSE39582	GSM972000	Cancer	Avaliable	WT	WT	WT	NA
1706	GSE39582	GSM972001	Cancer	Avaliable	WT	WT	MU	NA
1707	GSE39582	GSM972002	Cancer	Avaliable	MU	WT	MU	NA

1708	GSE39582	GSM972003	Cancer	NA	MU	WT	WT	NA
1709	GSE39582	GSM972004	Cancer	NA	MU	WT	MU	NA
1710	GSE39582	GSM972005	Cancer	NA	MU	WT	MU	NA
1711	GSE39582	GSM972006	Cancer	NA	WT	WT	WT	NA
1712	GSE39582	GSM972007	Cancer	NA	WT	MU	WT	NA
1713	GSE39582	GSM972008	Cancer	NA	WT	MU	MU	NA
1714	GSE39582	GSM972009	Cancer	NA	MU	WT	MU	NA
1715	GSE39582	GSM972010	Cancer	NA	MU	WT	WT	NA
1716	GSE39582	GSM972011	Cancer	NA	WT	MU	WT	NA
1717	GSE39582	GSM972012	Cancer	NA	MU	WT	WT	NA
1718	GSE39582	GSM972013	Cancer	NA	WT	WT	WT	NA
1719	GSE39582	GSM972014	Cancer	NA	WT	WT	MU	NA
1720	GSE39582	GSM972015	Cancer	Avaliable	MU	WT	WT	NA
1721	GSE39582	GSM972016	Cancer	Avaliable	WT	WT	MU	NA
1722	GSE39582	GSM972017	Cancer	Avaliable	MU	WT	NA	NA
1723	GSE39582	GSM972018	Cancer	Avaliable	WT	WT	NA	NA
1724	GSE39582	GSM972019	Cancer	Avaliable	WT	MU	NA	NA
1725	GSE39582	GSM972020	Cancer	Avaliable	WT	WT	NA	NA
1726	GSE39582	GSM972021	Cancer	Avaliable	WT	WT	NA	NA
1727	GSE39582	GSM972022	Cancer	Avaliable	MU	WT	NA	NA
1728	GSE39582	GSM972023	Cancer	Avaliable	MU	WT	NA	NA
1729	GSE39582	GSM972024	Cancer	Avaliable	WT	WT	NA	NA
1730	GSE39582	GSM972025	Cancer	Avaliable	WT	WT	NA	NA
1731	GSE39582	GSM972027	Cancer	Avaliable	WT	WT	NA	NA
1732	GSE39582	GSM972028	Cancer	Avaliable	MU	WT	NA	NA
1733	GSE39582	GSM972029	Cancer	Avaliable	WT	WT	NA	NA
1734	GSE39582	GSM972030	Cancer	Avaliable	WT	MU	NA	NA
1735	GSE39582	GSM972031	Cancer	Avaliable	NA	NA	NA	NA
1736	GSE39582	GSM972032	Cancer	Avaliable	WT	WT	NA	NA
1737	GSE39582	GSM972033	Cancer	Avaliable	NA	NA	NA	NA
1738	GSE39582	GSM972034	Cancer	Avaliable	MU	WT	NA	NA
1739	GSE39582	GSM972035	Cancer	Avaliable	WT	WT	NA	NA
1740	GSE39582	GSM972036	Cancer	Avaliable	NA	NA	NA	NA
1741	GSE39582	GSM972037	Cancer	Avaliable	MU	WT	NA	NA
1742	GSE39582	GSM972038	Cancer	Avaliable	WT	WT	NA	NA
1743	GSE39582	GSM972040	Cancer	Avaliable	WT	WT	NA	NA
1744	GSE39582	GSM972041	Cancer	Avaliable	MU	WT	NA	NA
1745	GSE39582	GSM972042	Cancer	Avaliable	MU	WT	NA	NA
1746	GSE39582	GSM972043	Cancer	NA	WT	WT	NA	NA
1747	GSE39582	GSM972044	Cancer	Avaliable	WT	WT	NA	NA
1748	GSE39582	GSM972045	Cancer	NA	MU	WT	NA	NA
1749	GSE39582	GSM972047	Cancer	Avaliable	MU	WT	NA	NA
1750	GSE39582	GSM972048	Cancer	NA	MU	WT	NA	NA
1751	GSE39582	GSM972049	Cancer	Avaliable	MU	WT	NA	NA
1752	GSE39582	GSM972050	Cancer	Avaliable	MU	WT	NA	NA

1753	GSE39582	GSM972051	Cancer	NA	MU	WT	NA	NA
1754	GSE39582	GSM972052	Cancer	Avaliable	MU	WT	NA	NA
1755	GSE39582	GSM972053	Cancer	Avaliable	WT	WT	NA	NA
1756	GSE39582	GSM972054	Cancer	Avaliable	MU	WT	NA	NA
1757	GSE39582	GSM972055	Cancer	Avaliable	WT	WT	NA	NA
1758	GSE39582	GSM972056	Cancer	Avaliable	WT	MU	NA	NA
1759	GSE39582	GSM972057	Cancer	Avaliable	WT	WT	NA	NA
1760	GSE39582	GSM972058	Cancer	Avaliable	WT	MU	NA	NA
1761	GSE39582	GSM972060	Cancer	Avaliable	WT	WT	NA	NA
1762	GSE39582	GSM972061	Cancer	Avaliable	WT	WT	NA	NA
1763	GSE39582	GSM972062	Cancer	Avaliable	MU	WT	NA	NA
1764	GSE39582	GSM972063	Cancer	Avaliable	MU	WT	NA	NA
1765	GSE39582	GSM972064	Cancer	NA	MU	WT	NA	NA
1766	GSE39582	GSM972065	Cancer	Avaliable	WT	WT	NA	NA
1767	GSE39582	GSM972066	Cancer	NA	WT	WT	NA	NA
1768	GSE39582	GSM972067	Cancer	Avaliable	WT	WT	NA	NA
1769	GSE39582	GSM972068	Cancer	Avaliable	MU	WT	NA	NA
1770	GSE39582	GSM972069	Cancer	Avaliable	MU	WT	NA	NA
1771	GSE39582	GSM972070	Cancer	Avaliable	MU	WT	NA	NA
1772	GSE39582	GSM972071	Cancer	Avaliable	WT	WT	NA	NA
1773	GSE39582	GSM972072	Cancer	Avaliable	WT	MU	NA	NA
1774	GSE39582	GSM972073	Cancer	Avaliable	MU	WT	NA	NA
1775	GSE39582	GSM972074	Cancer	Avaliable	WT	WT	NA	NA
1776	GSE39582	GSM972075	Cancer	Avaliable	WT	WT	NA	NA
1777	GSE39582	GSM972076	Cancer	Avaliable	WT	WT	NA	NA
1778	GSE39582	GSM972077	Cancer	Avaliable	MU	WT	NA	NA
1779	GSE39582	GSM972078	Cancer	Avaliable	WT	WT	NA	NA
1780	GSE39582	GSM972080	Cancer	Avaliable	MU	WT	NA	NA
1781	GSE39582	GSM972081	Cancer	Avaliable	MU	WT	NA	NA
1782	GSE39582	GSM972082	Cancer	Avaliable	MU	WT	NA	NA
1783	GSE39582	GSM972083	Cancer	Avaliable	WT	WT	NA	NA
1784	GSE39582	GSM972084	Cancer	Avaliable	WT	WT	NA	NA
1785	GSE39582	GSM972085	Cancer	Avaliable	WT	WT	NA	NA
1786	GSE39582	GSM972086	Cancer	Avaliable	WT	WT	NA	NA
1787	GSE39582	GSM972087	Cancer	Avaliable	WT	WT	NA	NA
1788	GSE39582	GSM972088	Cancer	Avaliable	WT	WT	NA	NA
1789	GSE39582	GSM972089	Cancer	Avaliable	WT	WT	NA	NA
1790	GSE39582	GSM972090	Cancer	Avaliable	WT	WT	NA	NA
1791	GSE39582	GSM972091	Cancer	Avaliable	MU	WT	NA	NA
1792	GSE39582	GSM972092	Cancer	Avaliable	MU	WT	NA	NA
1793	GSE39582	GSM972093	Cancer	Avaliable	WT	WT	NA	NA
1794	GSE39582	GSM972094	Cancer	Avaliable	MU	WT	NA	NA
1795	GSE39582	GSM972095	Cancer	Avaliable	MU	WT	NA	NA
1796	GSE39582	GSM972096	Cancer	Avaliable	WT	WT	NA	NA
1797	GSE39582	GSM972098	Cancer	Avaliable	MU	WT	NA	NA

1798	GSE39582	GSM972099	Cancer	Avaliable	WT	WT	NA	NA
1799	GSE39582	GSM972100	Cancer	Avaliable	MU	WT	NA	NA
1800	GSE39582	GSM972101	Cancer	Avaliable	NA	NA	NA	NA
1801	GSE39582	GSM972102	Cancer	Avaliable	NA	NA	NA	NA
1802	GSE39582	GSM972103	Cancer	Avaliable	MU	WT	NA	NA
1803	GSE39582	GSM972104	Cancer	Avaliable	MU	WT	NA	NA
1804	GSE39582	GSM972105	Cancer	Avaliable	WT	WT	NA	NA
1805	GSE39582	GSM972106	Cancer	Avaliable	NA	NA	NA	NA
1806	GSE39582	GSM972107	Cancer	Avaliable	WT	WT	NA	NA
1807	GSE39582	GSM972108	Cancer	Avaliable	NA	NA	NA	NA
1808	GSE39582	GSM972109	Cancer	Avaliable	WT	WT	NA	NA
1809	GSE39582	GSM972110	Cancer	Avaliable	NA	NA	NA	NA
1810	GSE39582	GSM972111	Cancer	Avaliable	NA	NA	NA	NA
1811	GSE39582	GSM972113	Cancer	Avaliable	NA	NA	NA	NA
1812	GSE39582	GSM972114	Cancer	Avaliable	WT	MU	NA	NA
1813	GSE39582	GSM972115	Cancer	Avaliable	WT	WT	NA	NA
1814	GSE39582	GSM972116	Cancer	Avaliable	WT	WT	NA	NA
1815	GSE39582	GSM972117	Cancer	Avaliable	WT	MU	NA	NA
1816	GSE39582	GSM972118	Cancer	Avaliable	MU	WT	NA	NA
1817	GSE39582	GSM972119	Cancer	Avaliable	NA	NA	NA	NA
1818	GSE39582	GSM972120	Cancer	Avaliable	NA	NA	NA	NA
1819	GSE39582	GSM972121	Cancer	Avaliable	MU	WT	NA	NA
1820	GSE39582	GSM972122	Cancer	Avaliable	NA	NA	NA	NA
1821	GSE39582	GSM972123	Cancer	Avaliable	NA	NA	NA	NA
1822	GSE39582	GSM972124	Cancer	Avaliable	WT	WT	NA	NA
1823	GSE39582	GSM972125	Cancer	Avaliable	WT	WT	NA	NA
1824	GSE39582	GSM972126	Cancer	Avaliable	NA	NA	NA	NA
1825	GSE39582	GSM972127	Cancer	Avaliable	MU	WT	NA	NA
1826	GSE39582	GSM972128	Cancer	Avaliable	WT	WT	NA	NA
1827	GSE39582	GSM972129	Cancer	Avaliable	MU	WT	NA	NA
1828	GSE39582	GSM972130	Cancer	Avaliable	WT	WT	NA	NA
1829	GSE39582	GSM972131	Cancer	Avaliable	MU	WT	NA	NA
1830	GSE39582	GSM972132	Cancer	Avaliable	MU	WT	NA	NA
1831	GSE39582	GSM972133	Cancer	Avaliable	WT	NA	MU	NA
1832	GSE39582	GSM972134	Cancer	Avaliable	WT	NA	MU	NA
1833	GSE39582	GSM972135	Cancer	Avaliable	MU	NA	MU	NA
1834	GSE39582	GSM972136	Cancer	Avaliable	WT	WT	WT	NA
1835	GSE39582	GSM972137	Cancer	Avaliable	WT	NA	MU	NA
1836	GSE39582	GSM972138	Cancer	Avaliable	WT	NA	WT	NA
1837	GSE39582	GSM972139	Cancer	Avaliable	WT	NA	MU	NA
1838	GSE39582	GSM972140	Cancer	Avaliable	MU	NA	WT	NA
1839	GSE39582	GSM972141	Cancer	Avaliable	WT	NA	MU	NA
1840	GSE39582	GSM972142	Cancer	Avaliable	MU	WT	MU	NA
1841	GSE39582	GSM972143	Cancer	Avaliable	WT	WT	WT	NA
1842	GSE39582	GSM972144	Cancer	Avaliable	WT	WT	MU	NA

1843	GSE39582	GSM972145	Cancer	Avaliable	WT	NA	MU	NA
1844	GSE39582	GSM972146	Cancer	Avaliable	MU	NA	MU	NA
1845	GSE39582	GSM972147	Cancer	Avaliable	WT	WT	MU	NA
1846	GSE39582	GSM972148	Cancer	Avaliable	MU	NA	MU	NA
1847	GSE39582	GSM972149	Cancer	Avaliable	WT	NA	WT	NA
1848	GSE39582	GSM972150	Cancer	Avaliable	WT	NA	MU	NA
1849	GSE39582	GSM972151	Cancer	Avaliable	MU	NA	WT	NA
1850	GSE39582	GSM972152	Cancer	Avaliable	MU	NA	WT	NA
1851	GSE39582	GSM972153	Cancer	Avaliable	WT	WT	MU	NA
1852	GSE39582	GSM972154	Cancer	Avaliable	MU	NA	MU	NA
1853	GSE39582	GSM972155	Cancer	Avaliable	MU	NA	MU	NA
1854	GSE39582	GSM972156	Cancer	Avaliable	WT	NA	WT	NA
1855	GSE39582	GSM972157	Cancer	Avaliable	WT	NA	WT	NA
1856	GSE39582	GSM972158	Cancer	Avaliable	MU	NA	MU	NA
1857	GSE39582	GSM972159	Cancer	Avaliable	MU	WT	WT	NA
1858	GSE39582	GSM972160	Cancer	Avaliable	WT	NA	MU	NA
1859	GSE39582	GSM972161	Cancer	Avaliable	MU	NA	WT	NA
1860	GSE39582	GSM972162	Cancer	Avaliable	WT	NA	MU	NA
1861	GSE39582	GSM972163	Cancer	Avaliable	WT	NA	MU	NA
1862	GSE39582	GSM972164	Cancer	Avaliable	WT	NA	WT	NA
1863	GSE39582	GSM972165	Cancer	Avaliable	WT	NA	MU	NA
1864	GSE39582	GSM972166	Cancer	Avaliable	MU	NA	WT	NA
1865	GSE39582	GSM972167	Cancer	Avaliable	WT	WT	WT	NA
1866	GSE39582	GSM972168	Cancer	Avaliable	WT	NA	WT	NA
1867	GSE39582	GSM972169	Cancer	Avaliable	WT	NA	WT	NA
1868	GSE39582	GSM972170	Cancer	Avaliable	WT	NA	WT	NA
1869	GSE39582	GSM972171	Cancer	Avaliable	WT	NA	WT	NA
1870	GSE39582	GSM972172	Cancer	Avaliable	MU	WT	MU	NA
1871	GSE39582	GSM972173	Cancer	Avaliable	MU	WT	WT	NA
1872	GSE39582	GSM972174	Cancer	Avaliable	WT	WT	MU	NA
1873	GSE39582	GSM972175	Cancer	Avaliable	MU	WT	WT	NA
1874	GSE39582	GSM972176	Cancer	Avaliable	WT	WT	MU	NA
1875	GSE39582	GSM972177	Cancer	Avaliable	MU	WT	MU	NA
1876	GSE39582	GSM972178	Cancer	Avaliable	MU	WT	WT	NA
1877	GSE39582	GSM972179	Cancer	Avaliable	WT	WT	MU	NA
1878	GSE39582	GSM972180	Cancer	Avaliable	WT	WT	MU	NA
1879	GSE39582	GSM972182	Cancer	Avaliable	WT	WT	WT	NA
1880	GSE39582	GSM972183	Cancer	Avaliable	MU	WT	MU	NA
1881	GSE39582	GSM972184	Cancer	Avaliable	WT	MU	WT	NA
1882	GSE39582	GSM972185	Cancer	Avaliable	WT	WT	MU	NA
1883	GSE39582	GSM972186	Cancer	Avaliable	MU	WT	WT	NA
1884	GSE39582	GSM972187	Cancer	Avaliable	WT	WT	MU	NA
1885	GSE39582	GSM972188	Cancer	Avaliable	MU	WT	WT	NA
1886	GSE39582	GSM972189	Cancer	Avaliable	WT	WT	MU	NA
1887	GSE39582	GSM972190	Cancer	Avaliable	WT	WT	MU	NA

1888	GSE39582	GSM972191	Cancer	Avaliable	MU	WT	WT	NA
1889	GSE39582	GSM972192	Cancer	Avaliable	MU	WT	MU	NA
1890	GSE39582	GSM972193	Cancer	Avaliable	MU	WT	MU	NA
1891	GSE39582	GSM972194	Cancer	Avaliable	WT	WT	MU	NA
1892	GSE39582	GSM972195	Cancer	Avaliable	MU	WT	WT	NA
1893	GSE39582	GSM972196	Cancer	Avaliable	MU	WT	WT	NA
1894	GSE39582	GSM972197	Cancer	Avaliable	WT	MU	WT	NA
1895	GSE39582	GSM972198	Cancer	Avaliable	MU	WT	MU	NA
1896	GSE39582	GSM972199	Cancer	Avaliable	WT	WT	MU	NA
1897	GSE39582	GSM972200	Cancer	Avaliable	WT	WT	WT	NA
1898	GSE39582	GSM972201	Cancer	Avaliable	WT	WT	WT	NA
1899	GSE39582	GSM972202	Cancer	Avaliable	WT	WT	MU	NA
1900	GSE39582	GSM972203	Cancer	Avaliable	WT	WT	MU	NA
1901	GSE39582	GSM972204	Cancer	Avaliable	WT	WT	MU	NA
1902	GSE39582	GSM972205	Cancer	Avaliable	WT	WT	WT	NA
1903	GSE39582	GSM972206	Cancer	NA	MU	WT	WT	NA
1904	GSE39582	GSM972207	Cancer	Avaliable	WT	WT	MU	NA
1905	GSE39582	GSM972208	Cancer	NA	WT	WT	MU	NA
1906	GSE39582	GSM972209	Cancer	NA	WT	MU	MU	NA
1907	GSE39582	GSM972210	Cancer	NA	WT	WT	MU	NA
1908	GSE39582	GSM972211	Cancer	Avaliable	WT	WT	MU	NA
1909	GSE39582	GSM972212	Cancer	NA	MU	WT	MU	NA
1910	GSE39582	GSM972213	Cancer	NA	MU	WT	MU	NA
1911	GSE39582	GSM972214	Cancer	NA	WT	MU	MU	NA
1912	GSE39582	GSM972215	Cancer	Avaliable	MU	WT	MU	NA
1913	GSE39582	GSM972216	Cancer	NA	WT	WT	MU	NA
1914	GSE39582	GSM972217	Cancer	NA	MU	WT	MU	NA
1915	GSE39582	GSM972218	Cancer	NA	MU	WT	WT	NA
1916	GSE39582	GSM972219	Cancer	NA	MU	WT	MU	NA
1917	GSE39582	GSM972220	Cancer	Avaliable	WT	MU	NA	NA
1918	GSE39582	GSM972221	Cancer	Avaliable	WT	MU	NA	NA
1919	GSE39582	GSM972222	Cancer	Avaliable	WT	WT	NA	NA
1920	GSE39582	GSM972223	Cancer	Avaliable	WT	MU	NA	NA
1921	GSE39582	GSM972224	Cancer	Avaliable	NA	NA	NA	NA
1922	GSE39582	GSM972225	Cancer	Avaliable	WT	WT	NA	NA
1923	GSE39582	GSM972226	Cancer	Avaliable	MU	WT	NA	NA
1924	GSE39582	GSM972227	Cancer	NA	MU	WT	NA	NA
1925	GSE39582	GSM972228	Cancer	Avaliable	WT	MU	NA	NA
1926	GSE39582	GSM972230	Cancer	Avaliable	WT	WT	NA	NA
1927	GSE39582	GSM972231	Cancer	NA	WT	WT	NA	NA
1928	GSE39582	GSM972232	Cancer	NA	MU	WT	NA	NA
1929	GSE39582	GSM972233	Cancer	Avaliable	WT	WT	NA	NA
1930	GSE39582	GSM972234	Cancer	NA	WT	WT	NA	NA
1931	GSE39582	GSM972235	Cancer	Avaliable	MU	WT	NA	NA
1932	GSE39582	GSM972236	Cancer	NA	WT	WT	NA	NA

1933	GSE39582	GSM972237	Cancer	Avaliable	WT	WT	NA	NA
1934	GSE39582	GSM972238	Cancer	Avaliable	WT	WT	NA	NA
1935	GSE39582	GSM972239	Cancer	Avaliable	WT	WT	NA	NA
1936	GSE39582	GSM972240	Cancer	NA	WT	WT	NA	NA
1937	GSE39582	GSM972241	Cancer	Avaliable	WT	WT	NA	NA
1938	GSE39582	GSM972242	Cancer	Avaliable	MU	WT	NA	NA
1939	GSE39582	GSM972243	Cancer	Avaliable	MU	WT	NA	NA
1940	GSE39582	GSM972244	Cancer	Avaliable	WT	WT	NA	NA
1941	GSE39582	GSM972245	Cancer	NA	MU	WT	NA	NA
1942	GSE39582	GSM972246	Cancer	NA	WT	WT	NA	NA
1943	GSE39582	GSM972247	Cancer	Avaliable	WT	WT	NA	NA
1944	GSE39582	GSM972248	Cancer	Avaliable	NA	NA	NA	NA
1945	GSE39582	GSM972249	Cancer	Avaliable	MU	WT	NA	NA
1946	GSE39582	GSM972250	Cancer	Avaliable	MU	WT	NA	NA
1947	GSE39582	GSM972251	Cancer	NA	MU	WT	NA	NA
1948	GSE39582	GSM972252	Cancer	NA	MU	WT	NA	NA
1949	GSE39582	GSM972253	Cancer	NA	WT	WT	NA	NA
1950	GSE39582	GSM972254	Cancer	NA	WT	WT	NA	NA
1951	GSE39582	GSM972255	Cancer	Avaliable	WT	WT	NA	NA
1952	GSE39582	GSM972256	Cancer	NA	WT	WT	NA	NA
1953	GSE39582	GSM972257	Cancer	Avaliable	MU	WT	NA	NA
1954	GSE39582	GSM972258	Cancer	NA	WT	WT	NA	NA
1955	GSE39582	GSM972259	Cancer	Avaliable	MU	WT	NA	NA
1956	GSE39582	GSM972260	Cancer	NA	MU	WT	NA	NA
1957	GSE39582	GSM972261	Cancer	Avaliable	WT	MU	NA	NA
1958	GSE39582	GSM972262	Cancer	Avaliable	WT	MU	NA	NA
1959	GSE39582	GSM972263	Cancer	Avaliable	WT	WT	NA	NA
1960	GSE39582	GSM972264	Cancer	Avaliable	WT	WT	NA	NA
1961	GSE39582	GSM972265	Cancer	Avaliable	WT	MU	NA	NA
1962	GSE39582	GSM972266	Cancer	NA	WT	WT	NA	NA
1963	GSE39582	GSM972267	Cancer	Avaliable	WT	WT	NA	NA
1964	GSE39582	GSM972268	Cancer	Avaliable	MU	WT	NA	NA
1965	GSE39582	GSM972269	Cancer	Avaliable	MU	WT	NA	NA
1966	GSE39582	GSM972270	Cancer	Avaliable	WT	MU	NA	NA
1967	GSE39582	GSM972271	Cancer	Avaliable	MU	WT	NA	NA
1968	GSE39582	GSM972272	Cancer	NA	MU	WT	NA	NA
1969	GSE39582	GSM972273	Cancer	Avaliable	MU	WT	NA	NA
1970	GSE39582	GSM972274	Cancer	Avaliable	WT	MU	NA	NA
1971	GSE39582	GSM972275	Cancer	Avaliable	WT	MU	NA	NA
1972	GSE39582	GSM972276	Cancer	Avaliable	MU	WT	NA	NA
1973	GSE39582	GSM972277	Cancer	Avaliable	WT	MU	NA	NA
1974	GSE39582	GSM972278	Cancer	Avaliable	WT	MU	NA	NA
1975	GSE39582	GSM972279	Cancer	Avaliable	WT	MU	NA	NA
1976	GSE39582	GSM972280	Cancer	Avaliable	MU	WT	NA	NA
1977	GSE39582	GSM972281	Cancer	Avaliable	WT	WT	NA	NA

1978	GSE39582	GSM972282	Cancer	Avaliable	MU	WT	NA	NA
1979	GSE39582	GSM972283	Cancer	Avaliable	WT	WT	NA	NA
1980	GSE39582	GSM972284	Cancer	NA	MU	WT	NA	NA
1981	GSE39582	GSM972285	Cancer	Avaliable	WT	WT	NA	NA
1982	GSE39582	GSM972286	Cancer	Avaliable	MU	WT	NA	NA
1983	GSE39582	GSM972287	Cancer	Avaliable	WT	WT	NA	NA
1984	GSE39582	GSM972288	Cancer	NA	WT	WT	NA	NA
1985	GSE39582	GSM972289	Cancer	NA	MU	WT	NA	NA
1986	GSE39582	GSM972290	Cancer	Avaliable	WT	WT	NA	NA
1987	GSE39582	GSM972291	Cancer	Avaliable	MU	WT	NA	NA
1988	GSE39582	GSM972292	Cancer	Avaliable	WT	MU	NA	NA
1989	GSE39582	GSM972293	Cancer	Avaliable	MU	WT	NA	NA
1990	GSE39582	GSM972294	Cancer	Avaliable	WT	MU	NA	NA
1991	GSE39582	GSM972295	Cancer	Avaliable	MU	WT	NA	NA
1992	GSE39582	GSM972296	Cancer	Avaliable	MU	WT	NA	NA
1993	GSE39582	GSM972297	Cancer	Avaliable	MU	WT	NA	NA
1994	GSE39582	GSM972298	Cancer	Avaliable	NA	NA	NA	NA
1995	GSE39582	GSM972299	Cancer	NA	WT	WT	NA	NA
1996	GSE39582	GSM972300	Cancer	Avaliable	MU	WT	NA	NA
1997	GSE39582	GSM972301	Cancer	Avaliable	WT	WT	MU	NA
1998	GSE39582	GSM972302	Cancer	Avaliable	MU	WT	WT	NA
1999	GSE39582	GSM972303	Cancer	Avaliable	WT	WT	MU	NA
2000	GSE39582	GSM972304	Cancer	Avaliable	MU	WT	WT	NA
2001	GSE39582	GSM972305	Cancer	Avaliable	WT	MU	WT	NA
2002	GSE39582	GSM972306	Cancer	Avaliable	WT	WT	MU	NA
2003	GSE39582	GSM972307	Cancer	Avaliable	WT	WT	WT	NA
2004	GSE39582	GSM972308	Cancer	Avaliable	WT	WT	WT	NA
2005	GSE39582	GSM972309	Cancer	Avaliable	WT	WT	WT	NA
2006	GSE39582	GSM972310	Cancer	Avaliable	MU	WT	MU	NA
2007	GSE39582	GSM972312	Cancer	Avaliable	WT	WT	MU	NA
2008	GSE39582	GSM972313	Cancer	Avaliable	WT	WT	MU	NA
2009	GSE39582	GSM972314	Cancer	Avaliable	WT	MU	WT	NA
2010	GSE39582	GSM972315	Cancer	Avaliable	MU	WT	WT	NA
2011	GSE39582	GSM972316	Cancer	Avaliable	WT	MU	WT	NA
2012	GSE39582	GSM972317	Cancer	Avaliable	WT	MU	WT	NA
2013	GSE39582	GSM972318	Cancer	Avaliable	MU	WT	WT	NA
2014	GSE39582	GSM972319	Cancer	Avaliable	MU	WT	MU	NA
2015	GSE39582	GSM972320	Cancer	Avaliable	MU	WT	MU	NA
2016	GSE39582	GSM972321	Cancer	Avaliable	WT	WT	MU	NA
2017	GSE39582	GSM972322	Cancer	Avaliable	MU	WT	WT	NA
2018	GSE39582	GSM972323	Cancer	Avaliable	WT	WT	MU	NA
2019	GSE39582	GSM972324	Cancer	Avaliable	MU	WT	WT	NA
2020	GSE39582	GSM972325	Cancer	Avaliable	MU	WT	WT	NA
2021	GSE39582	GSM972326	Cancer	Avaliable	MU	WT	MU	NA
2022	GSE39582	GSM972327	Cancer	Avaliable	WT	WT	WT	NA

2023	GSE39582	GSM972328	Cancer	Avaliable	WT	WT	WT	NA
2024	GSE39582	GSM972329	Cancer	Avaliable	WT	WT	WT	NA
2025	GSE39582	GSM972330	Cancer	Avaliable	MU	WT	WT	NA
2026	GSE39582	GSM972331	Cancer	Avaliable	WT	WT	WT	NA
2027	GSE39582	GSM972332	Cancer	Avaliable	WT	MU	MU	NA
2028	GSE39582	GSM972333	Cancer	Avaliable	WT	WT	MU	NA
2029	GSE39582	GSM972334	Cancer	Avaliable	WT	MU	WT	NA
2030	GSE39582	GSM972335	Cancer	Avaliable	WT	WT	MU	NA
2031	GSE39582	GSM972336	Cancer	Avaliable	WT	WT	MU	NA
2032	GSE39582	GSM972337	Cancer	Avaliable	WT	MU	WT	NA
2033	GSE39582	GSM972338	Cancer	Avaliable	WT	WT	MU	NA
2034	GSE39582	GSM972339	Cancer	Avaliable	MU	WT	MU	NA
2035	GSE39582	GSM972340	Cancer	Avaliable	WT	WT	WT	NA
2036	GSE39582	GSM972341	Cancer	Avaliable	WT	WT	MU	NA
2037	GSE39582	GSM972342	Cancer	Avaliable	WT	WT	WT	NA
2038	GSE39582	GSM972343	Cancer	Avaliable	MU	WT	MU	NA
2039	GSE39582	GSM972344	Cancer	Avaliable	WT	WT	MU	NA
2040	GSE39582	GSM972345	Cancer	Avaliable	MU	WT	WT	NA
2041	GSE39582	GSM972346	Cancer	Avaliable	WT	MU	MU	NA
2042	GSE39582	GSM972347	Cancer	Avaliable	WT	WT	MU	NA
2043	GSE39582	GSM972348	Cancer	Avaliable	MU	WT	WT	NA
2044	GSE39582	GSM972349	Cancer	Avaliable	MU	WT	WT	NA
2045	GSE39582	GSM972350	Cancer	NA	WT	WT	WT	NA
2046	GSE39582	GSM972351	Cancer	Avaliable	WT	WT	NA	NA
2047	GSE39582	GSM972352	Cancer	Avaliable	WT	WT	MU	NA
2048	GSE39582	GSM972353	Cancer	Avaliable	MU	WT	WT	NA
2049	GSE39582	GSM972354	Cancer	Avaliable	WT	WT	MU	NA
2050	GSE39582	GSM972355	Cancer	Avaliable	MU	WT	MU	NA
2051	GSE39582	GSM972356	Cancer	NA	WT	WT	MU	NA
2052	GSE39582	GSM972357	Cancer	Avaliable	WT	WT	WT	NA
2053	GSE39582	GSM972358	Cancer	Avaliable	WT	WT	MU	NA
2054	GSE39582	GSM972359	Cancer	Avaliable	WT	WT	WT	NA
2055	GSE39582	GSM972360	Cancer	Avaliable	WT	WT	MU	NA
2056	GSE39582	GSM972362	Cancer	Avaliable	MU	WT	MU	NA
2057	GSE39582	GSM972363	Cancer	Avaliable	MU	WT	MU	NA
2058	GSE39582	GSM972364	Cancer	Avaliable	MU	WT	MU	NA
2059	GSE39582	GSM972365	Cancer	Avaliable	MU	WT	MU	NA
2060	GSE39582	GSM972366	Cancer	Avaliable	MU	WT	WT	NA
2061	GSE39582	GSM972367	Cancer	Avaliable	MU	WT	MU	NA
2062	GSE39582	GSM972368	Cancer	Avaliable	WT	WT	MU	NA
2063	GSE39582	GSM972369	Cancer	Avaliable	WT	WT	MU	NA
2064	GSE39582	GSM972370	Cancer	Avaliable	WT	WT	MU	NA
2065	GSE39582	GSM972371	Cancer	Avaliable	WT	WT	MU	NA
2066	GSE39582	GSM972372	Cancer	Avaliable	MU	WT	MU	NA
2067	GSE39582	GSM972373	Cancer	Avaliable	WT	WT	MU	NA

2068	GSE39582	GSM972374	Cancer	Avaliable	WT	WT	WT	NA
2069	GSE39582	GSM972375	Cancer	Avaliable	MU	WT	WT	NA
2070	GSE39582	GSM972376	Cancer	Avaliable	WT	WT	MU	NA
2071	GSE39582	GSM972377	Cancer	Avaliable	WT	WT	WT	NA
2072	GSE39582	GSM972378	Cancer	Avaliable	WT	WT	WT	NA
2073	GSE39582	GSM972379	Cancer	Avaliable	MU	WT	MU	NA
2074	GSE39582	GSM972380	Cancer	Avaliable	MU	WT	MU	NA
2075	GSE39582	GSM972381	Cancer	Avaliable	WT	WT	MU	NA
2076	GSE39582	GSM972382	Cancer	Avaliable	MU	WT	MU	NA
2077	GSE39582	GSM972383	Cancer	Avaliable	WT	WT	MU	NA
2078	GSE39582	GSM972384	Cancer	Avaliable	WT	WT	MU	NA
2079	GSE39582	GSM972385	Cancer	Avaliable	WT	WT	MU	NA
2080	GSE39582	GSM972386	Cancer	Avaliable	MU	WT	MU	NA
2081	GSE39582	GSM972387	Cancer	Avaliable	WT	WT	MU	NA
2082	GSE39582	GSM972388	Cancer	Avaliable	WT	WT	MU	NA
2083	GSE39582	GSM972389	Cancer	Avaliable	MU	WT	WT	NA
2084	GSE39582	GSM972390	Cancer	Avaliable	MU	WT	MU	NA
2085	GSE39582	GSM972391	Cancer	Avaliable	WT	MU	WT	NA
2086	GSE39582	GSM972392	Cancer	Avaliable	MU	WT	WT	NA
2087	GSE39582	GSM972393	Cancer	Avaliable	MU	WT	WT	NA
2088	GSE39582	GSM972394	Cancer	Avaliable	MU	WT	MU	NA
2089	GSE39582	GSM972395	Cancer	Avaliable	WT	WT	MU	NA
2090	GSE39582	GSM972396	Cancer	Avaliable	WT	WT	WT	NA
2091	GSE39582	GSM972397	Cancer	Avaliable	MU	WT	MU	NA
2092	GSE39582	GSM972398	Cancer	Avaliable	WT	WT	MU	NA
2093	GSE39582	GSM972399	Cancer	Avaliable	WT	WT	MU	NA
2094	GSE39582	GSM972400	Cancer	NA	MU	WT	MU	NA
2095	GSE39582	GSM972402	Cancer	NA	MU	WT	NA	NA
2096	GSE39582	GSM972403	Cancer	Avaliable	MU	WT	NA	NA
2097	GSE39582	GSM972404	Cancer	Avaliable	WT	WT	NA	NA
2098	GSE39582	GSM972405	Cancer	Avaliable	WT	WT	NA	NA
2099	GSE39582	GSM972407	Cancer	Avaliable	WT	WT	NA	NA
2100	GSE39582	GSM972408	Cancer	Avaliable	WT	WT	NA	NA
2101	GSE39582	GSM972409	Cancer	Avaliable	WT	WT	NA	NA
2102	GSE39582	GSM972410	Cancer	Avaliable	WT	WT	NA	NA
2103	GSE39582	GSM972411	Cancer	Avaliable	MU	NA	MU	NA
2104	GSE39582	GSM972412	Cancer	Avaliable	WT	NA	MU	NA
2105	GSE39582	GSM972413	Cancer	Avaliable	WT	WT	MU	NA
2106	GSE39582	GSM972414	Cancer	Avaliable	WT	WT	WT	NA
2107	GSE39582	GSM972415	Cancer	Avaliable	MU	NA	MU	NA
2108	GSE39582	GSM972416	Cancer	Avaliable	MU	WT	MU	NA
2109	GSE39582	GSM972417	Cancer	Avaliable	WT	WT	WT	NA
2110	GSE39582	GSM972418	Cancer	Avaliable	WT	WT	MU	NA
2111	GSE39582	GSM972419	Cancer	Avaliable	WT	WT	MU	NA
2112	GSE39582	GSM972420	Cancer	Avaliable	WT	MU	WT	NA

2113	GSE39582	GSM972421	Cancer	Avaliable	WT	WT	MU	NA
2114	GSE39582	GSM972422	Cancer	Avaliable	MU	WT	WT	NA
2115	GSE39582	GSM972423	Cancer	Avaliable	WT	WT	MU	NA
2116	GSE39582	GSM972424	Cancer	Avaliable	WT	WT	MU	NA
2117	GSE39582	GSM972425	Cancer	Avaliable	MU	WT	WT	NA
2118	GSE39582	GSM972426	Cancer	Avaliable	WT	WT	MU	NA
2119	GSE39582	GSM972427	Cancer	Avaliable	WT	WT	MU	NA
2120	GSE39582	GSM972428	Cancer	Avaliable	WT	WT	MU	NA
2121	GSE39582	GSM972429	Cancer	Avaliable	MU	WT	MU	NA
2122	GSE39582	GSM972430	Cancer	Avaliable	WT	WT	WT	NA
2123	GSE39582	GSM972431	Cancer	Avaliable	MU	WT	WT	NA
2124	GSE39582	GSM972432	Cancer	Avaliable	MU	WT	MU	NA
2125	GSE39582	GSM972433	Cancer	Avaliable	WT	WT	WT	NA
2126	GSE39582	GSM972434	Cancer	Avaliable	WT	WT	MU	NA
2127	GSE39582	GSM972435	Cancer	Avaliable	WT	WT	MU	NA
2128	GSE39582	GSM972436	Cancer	Avaliable	MU	WT	MU	NA
2129	GSE39582	GSM972437	Cancer	NA	WT	WT	WT	NA
2130	GSE39582	GSM972439	Cancer	NA	WT	WT	MU	NA
2131	GSE39582	GSM972440	Cancer	Avaliable	MU	WT	MU	NA
2132	GSE39582	GSM972441	Cancer	NA	WT	WT	WT	NA
2133	GSE39582	GSM972442	Cancer	NA	MU	WT	WT	NA
2134	GSE39582	GSM972443	Cancer	Avaliable	WT	WT	NA	NA
2135	GSE39582	GSM972444	Cancer	Avaliable	MU	WT	NA	NA
2136	GSE39582	GSM972445	Cancer	Avaliable	WT	WT	NA	NA
2137	GSE39582	GSM972446	Cancer	NA	MU	WT	NA	NA
2138	GSE39582	GSM972447	Cancer	Avaliable	WT	MU	NA	NA
2139	GSE39582	GSM972448	Cancer	NA	WT	MU	WT	NA
2140	GSE39582	GSM972449	Cancer	Avaliable	MU	WT	MU	NA
2141	GSE39582	GSM972450	Cancer	Avaliable	WT	WT	MU	NA
2142	GSE39582	GSM972451	Cancer	Avaliable	WT	WT	WT	NA
2143	GSE39582	GSM972452	Cancer	Avaliable	WT	MU	WT	NA
2144	GSE39582	GSM972453	Cancer	Avaliable	MU	WT	WT	NA
2145	GSE39582	GSM972454	Cancer	Avaliable	MU	WT	MU	NA
2146	GSE39582	GSM972455	Cancer	Avaliable	WT	MU	WT	NA
2147	GSE39582	GSM972456	Cancer	NA	WT	WT	MU	NA
2148	GSE39582	GSM972457	Cancer	Avaliable	MU	WT	WT	NA
2149	GSE39582	GSM972458	Cancer	Avaliable	MU	WT	WT	NA
2150	GSE39582	GSM972459	Cancer	Avaliable	WT	WT	WT	NA
2151	GSE39582	GSM972460	Cancer	Avaliable	WT	WT	WT	NA
2152	GSE39582	GSM972461	Cancer	Avaliable	WT	WT	MU	NA
2153	GSE39582	GSM972462	Cancer	Avaliable	MU	WT	MU	NA
2154	GSE39582	GSM972463	Cancer	NA	MU	WT	WT	NA
2155	GSE39582	GSM972464	Cancer	Avaliable	WT	WT	MU	NA
2156	GSE39582	GSM972465	Cancer	Avaliable	WT	WT	NA	NA
2157	GSE39582	GSM972466	Cancer	Avaliable	MU	WT	MU	NA

2158	GSE39582	GSM972467	Cancer	Avaliable	MU	WT	MU	NA
2159	GSE39582	GSM972468	Cancer	Avaliable	WT	WT	MU	NA
2160	GSE39582	GSM972469	Cancer	Avaliable	MU	WT	WT	NA
2161	GSE39582	GSM972470	Cancer	Avaliable	WT	WT	MU	NA
2162	GSE39582	GSM972471	Cancer	NA	WT	MU	WT	NA
2163	GSE39582	GSM972472	Cancer	Avaliable	WT	WT	WT	NA
2164	GSE39582	GSM972473	Cancer	Avaliable	MU	WT	WT	NA
2165	GSE39582	GSM972474	Cancer	Avaliable	WT	WT	MU	NA
2166	GSE39582	GSM972475	Cancer	Avaliable	MU	WT	WT	NA
2167	GSE39582	GSM972476	Cancer	Avaliable	WT	WT	WT	NA
2168	GSE39582	GSM972477	Cancer	Avaliable	MU	WT	MU	NA
2169	GSE39582	GSM972478	Cancer	Avaliable	WT	WT	MU	NA
2170	GSE39582	GSM972480	Cancer	Avaliable	MU	WT	WT	NA
2171	GSE39582	GSM972481	Cancer	Avaliable	WT	WT	MU	NA
2172	GSE39582	GSM972482	Cancer	Avaliable	MU	WT	WT	NA
2173	GSE39582	GSM972483	Cancer	Avaliable	WT	WT	MU	NA
2174	GSE39582	GSM972484	Cancer	Avaliable	WT	WT	WT	NA
2175	GSE39582	GSM972485	Cancer	Avaliable	MU	WT	WT	NA
2176	GSE39582	GSM972486	Cancer	Avaliable	NA	WT	WT	NA
2177	GSE39582	GSM972487	Cancer	Avaliable	MU	WT	WT	NA
2178	GSE39582	GSM972488	Cancer	Avaliable	MU	WT	WT	NA
2179	GSE39582	GSM972489	Cancer	Avaliable	MU	WT	WT	NA
2180	GSE39582	GSM972490	Cancer	Avaliable	WT	WT	MU	NA
2181	GSE39582	GSM972491	Cancer	Avaliable	WT	WT	MU	NA
2182	GSE39582	GSM972492	Cancer	Avaliable	WT	WT	MU	NA
2183	GSE39582	GSM972493	Cancer	Avaliable	MU	WT	WT	NA
2184	GSE39582	GSM972494	Cancer	Avaliable	WT	WT	WT	NA
2185	GSE39582	GSM972495	Cancer	Avaliable	WT	WT	WT	NA
2186	GSE39582	GSM972496	Cancer	Avaliable	WT	WT	WT	NA
2187	GSE39582	GSM972497	Cancer	Avaliable	NA	WT	MU	NA
2188	GSE39582	GSM972498	Cancer	Avaliable	WT	WT	MU	NA
2189	GSE39582	GSM972499	Cancer	Avaliable	WT	WT	WT	NA
2190	GSE39582	GSM972500	Cancer	NA	WT	WT	NA	NA
2191	GSE39582	GSM972501	Cancer	Avaliable	WT	WT	MU	NA
2192	GSE39582	GSM972502	Cancer	Avaliable	WT	WT	WT	NA
2193	GSE39582	GSM972503	Cancer	Avaliable	WT	WT	WT	NA
2194	GSE39582	GSM972504	Cancer	Avaliable	MU	WT	WT	NA
2195	GSE39582	GSM972505	Cancer	Avaliable	WT	WT	WT	NA
2196	GSE39582	GSM972506	Cancer	Avaliable	WT	WT	MU	NA
2197	GSE39582	GSM972507	Cancer	Avaliable	WT	MU	WT	NA
2198	GSE39582	GSM972508	Cancer	Avaliable	MU	WT	MU	NA
2199	GSE39582	GSM972509	Cancer	Avaliable	WT	WT	WT	NA
2200	GSE39582	GSM972510	Cancer	Avaliable	WT	WT	WT	NA
2201	GSE39582	GSM972511	Cancer	Avaliable	WT	WT	MU	NA
2202	GSE39582	GSM972512	Cancer	Avaliable	MU	WT	WT	NA

2203	GSE39582	GSM972513	Cancer	Avaliable	MU	WT	MU	NA
2204	GSE39582	GSM972514	Cancer	NA	WT	WT	MU	NA
2205	GSE39582	GSM972515	Cancer	Avaliable	WT	WT	MU	NA
2206	GSE39582	GSM972516	Cancer	Avaliable	MU	WT	WT	NA
2207	GSE39582	GSM972517	Cancer	Avaliable	WT	WT	MU	NA
2208	GSE39582	GSM972518	Cancer	Avaliable	MU	WT	MU	NA
2209	GSE39582	GSM972519	Cancer	Avaliable	WT	WT	MU	NA
2210	GSE39582	GSM972520	Cancer	Avaliable	WT	WT	MU	NA
2211	GSE39582	GSM972521	Cancer	Avaliable	WT	WT	MU	NA
2212	GSE39582	GSM972522	Cancer	Avaliable	MU	WT	WT	NA
2213	GSE4107	GSM93920	Cancer	NA	NA	NA	NA	NA
2214	GSE4107	GSM93921	Cancer	NA	NA	NA	NA	NA
2215	GSE4107	GSM93922	Cancer	NA	NA	NA	NA	NA
2216	GSE4107	GSM93923	Cancer	NA	NA	NA	NA	NA
2217	GSE4107	GSM93924	Cancer	NA	NA	NA	NA	NA
2218	GSE4107	GSM93927	Cancer	NA	NA	NA	NA	NA
2219	GSE4107	GSM93928	Cancer	NA	NA	NA	NA	NA
2220	GSE4107	GSM93929	Cancer	NA	NA	NA	NA	NA
2221	GSE4107	GSM93932	Cancer	NA	NA	NA	NA	NA
2222	GSE41328	GSM1014803	Cancer	NA	NA	NA	NA	NA
2223	GSE41328	GSM1014804	Cancer	NA	NA	NA	NA	NA
2224	GSE41328	GSM1014805	Cancer	NA	NA	NA	NA	NA
2225	GSE41328	GSM1014806	Cancer	NA	NA	NA	NA	NA
2226	GSE41328	GSM1014807	Cancer	NA	NA	NA	NA	NA
2227	GSE41328	GSM1014813	Cancer	NA	NA	NA	NA	NA
2228	GSE41328	GSM1014814	Cancer	NA	NA	NA	NA	NA
2229	GSE41328	GSM1014815	Cancer	NA	NA	NA	NA	NA
2230	GSE41328	GSM1014816	Cancer	NA	NA	NA	NA	NA
2231	GSE41328	GSM1014817	Cancer	NA	NA	NA	NA	NA
2232	GSE4183	GSM95496	Cancer	NA	NA	NA	NA	NA
2233	GSE4183	GSM95497	Cancer	NA	NA	NA	NA	NA
2234	GSE4183	GSM95498	Cancer	NA	NA	NA	NA	NA
2235	GSE4183	GSM95499	Cancer	NA	NA	NA	NA	NA
2236	GSE4183	GSM95500	Cancer	NA	NA	NA	NA	NA
2237	GSE4183	GSM95501	Cancer	NA	NA	NA	NA	NA
2238	GSE4183	GSM95502	Cancer	NA	NA	NA	NA	NA
2239	GSE4183	GSM95503	Cancer	NA	NA	NA	NA	NA
2240	GSE4183	GSM95504	Cancer	NA	NA	NA	NA	NA
2241	GSE4183	GSM95505	Cancer	NA	NA	NA	NA	NA
2242	GSE4183	GSM95506	Cancer	NA	NA	NA	NA	NA
2243	GSE4183	GSM95507	Cancer	NA	NA	NA	NA	NA
2244	GSE4183	GSM95508	Cancer	NA	NA	NA	NA	NA
2245	GSE4183	GSM95509	Cancer	NA	NA	NA	NA	NA
2246	GSE4183	GSM95510	Cancer	NA	NA	NA	NA	NA
2247	GSE52735	GSM1275067	Cancer	NA	NA	NA	NA	NA

2248	GSE52735	GSM1275068	Cancer	NA	NA	NA	NA	NA
2249	GSE52735	GSM1275069	Cancer	NA	NA	NA	NA	NA
2250	GSE52735	GSM1275070	Cancer	NA	NA	NA	NA	NA
2251	GSE52735	GSM1275071	Cancer	NA	NA	NA	NA	NA
2252	GSE52735	GSM1275072	Cancer	NA	NA	NA	NA	NA
2253	GSE52735	GSM1275073	Cancer	NA	NA	NA	NA	NA
2254	GSE52735	GSM1275074	Cancer	NA	NA	NA	NA	NA
2255	GSE52735	GSM1275075	Cancer	NA	NA	NA	NA	NA
2256	GSE52735	GSM1275076	Cancer	NA	NA	NA	NA	NA
2257	GSE52735	GSM1275077	Cancer	NA	NA	NA	NA	NA
2258	GSE52735	GSM1275078	Cancer	NA	NA	NA	NA	NA
2259	GSE52735	GSM1275080	Cancer	NA	NA	NA	NA	NA
2260	GSE52735	GSM1275081	Cancer	NA	NA	NA	NA	NA
2261	GSE52735	GSM1275082	Cancer	NA	NA	NA	NA	NA
2262	GSE52735	GSM1275083	Cancer	NA	NA	NA	NA	NA
2263	GSE52735	GSM1275085	Cancer	NA	NA	NA	NA	NA
2264	GSE52735	GSM1275087	Cancer	NA	NA	NA	NA	NA
2265	GSE52735	GSM1275089	Cancer	NA	NA	NA	NA	NA
2266	GSE52735	GSM1275092	Cancer	NA	NA	NA	NA	NA
2267	GSE52735	GSM1275093	Cancer	NA	NA	NA	NA	NA
2268	GSE52735	GSM1275095	Cancer	NA	NA	NA	NA	NA
2269	GSE52735	GSM1275096	Cancer	NA	NA	NA	NA	NA
2270	GSE52735	GSM1275097	Cancer	NA	NA	NA	NA	NA
2271	GSE52735	GSM1275098	Cancer	NA	NA	NA	NA	NA
2272	GSE52735	GSM1275099	Cancer	NA	NA	NA	NA	NA
2273	GSE52735	GSM1275100	Cancer	NA	NA	NA	NA	NA
2274	GSE52735	GSM1275101	Cancer	NA	NA	NA	NA	NA
2275	GSE52735	GSM1275102	Cancer	NA	NA	NA	NA	NA
2276	GSE52735	GSM1275103	Cancer	NA	NA	NA	NA	NA
2277	GSE54483	GSM1316660	Cancer	NA	NA	NA	NA	NA
2278	GSE54483	GSM1316661	Cancer	NA	NA	NA	NA	NA
2279	GSE60697	GSM1486028	Cancer	NA	NA	NA	NA	NA
2280	GSE60697	GSM1486029	Cancer	NA	NA	NA	NA	NA
2281	GSE60697	GSM1486030	Cancer	NA	NA	NA	NA	NA
2282	GSE60697	GSM1486031	Cancer	NA	NA	NA	NA	NA
2283	GSE60697	GSM1486032	Cancer	NA	NA	NA	NA	NA
2284	GSE60697	GSM1486033	Cancer	NA	NA	NA	NA	NA
2285	GSE60697	GSM1486034	Cancer	NA	NA	NA	NA	NA
2286	GSE60697	GSM1486035	Cancer	NA	NA	NA	NA	NA
2287	GSE60697	GSM1486036	Cancer	NA	NA	NA	NA	NA
2288	GSE60697	GSM1486037	Cancer	NA	NA	NA	NA	NA
2289	GSE60697	GSM1486038	Cancer	NA	NA	NA	NA	NA
2290	GSE60697	GSM1486039	Cancer	NA	NA	NA	NA	NA
2291	GSE60697	GSM1486040	Cancer	NA	NA	NA	NA	NA
2292	GSE60697	GSM1486041	Cancer	NA	NA	NA	NA	NA

2293	GSE60697	GSM1486042	Cancer	NA	NA	NA	NA	NA
2294	GSE60697	GSM1486043	Cancer	NA	NA	NA	NA	NA
2295	GSE60697	GSM1486044	Cancer	NA	NA	NA	NA	NA
2296	GSE60697	GSM1486045	Cancer	NA	NA	NA	NA	NA
2297	GSE60697	GSM1486046	Cancer	NA	NA	NA	NA	NA
2298	GSE60697	GSM1486047	Cancer	NA	NA	NA	NA	NA
2299	GSE62080	GSM1519418	Cancer	NA	NA	NA	NA	NA
2300	GSE62080	GSM1519419	Cancer	NA	NA	NA	NA	NA
2301	GSE62080	GSM1519420	Cancer	NA	NA	NA	NA	NA
2302	GSE62080	GSM1519421	Cancer	NA	NA	NA	NA	NA
2303	GSE62080	GSM1519423	Cancer	NA	NA	NA	NA	NA
2304	GSE62080	GSM1519424	Cancer	NA	NA	NA	NA	NA
2305	GSE62080	GSM1519425	Cancer	NA	NA	NA	NA	NA
2306	GSE62080	GSM1519426	Cancer	NA	NA	NA	NA	NA
2307	GSE62080	GSM1519427	Cancer	NA	NA	NA	NA	NA
2308	GSE62080	GSM1519428	Cancer	NA	NA	NA	NA	NA
2309	GSE62080	GSM1519429	Cancer	NA	NA	NA	NA	NA
2310	GSE62080	GSM1519430	Cancer	NA	NA	NA	NA	NA
2311	GSE62080	GSM1519431	Cancer	NA	NA	NA	NA	NA
2312	GSE62080	GSM1519432	Cancer	NA	NA	NA	NA	NA
2313	GSE62080	GSM1519433	Cancer	NA	NA	NA	NA	NA
2314	GSE62080	GSM1519434	Cancer	NA	NA	NA	NA	NA
2315	GSE62080	GSM1519435	Cancer	NA	NA	NA	NA	NA
2316	GSE62080	GSM1519436	Cancer	NA	NA	NA	NA	NA
2317	GSE62080	GSM1519437	Cancer	NA	NA	NA	NA	NA
2318	GSE62080	GSM1519438	Cancer	NA	NA	NA	NA	NA
2319	GSE64857	GSM1581684	Cancer	NA	NA	NA	NA	NA
2320	GSE64857	GSM1581685	Cancer	NA	NA	NA	NA	NA
2321	GSE64857	GSM1581686	Cancer	NA	NA	NA	NA	NA
2322	GSE64857	GSM1581687	Cancer	NA	NA	NA	NA	NA
2323	GSE64857	GSM1581688	Cancer	NA	NA	NA	NA	NA
2324	GSE64857	GSM1581689	Cancer	NA	NA	NA	NA	NA
2325	GSE64857	GSM1581690	Cancer	NA	NA	NA	NA	NA
2326	GSE64857	GSM1581691	Cancer	NA	NA	NA	NA	NA
2327	GSE64857	GSM1581692	Cancer	NA	NA	NA	NA	NA
2328	GSE64857	GSM1581693	Cancer	NA	NA	NA	NA	NA
2329	GSE64857	GSM1581695	Cancer	NA	NA	NA	NA	NA
2330	GSE64857	GSM1581696	Cancer	NA	NA	NA	NA	NA
2331	GSE64857	GSM1581697	Cancer	NA	NA	NA	NA	NA
2332	GSE64857	GSM1581698	Cancer	NA	NA	NA	NA	NA
2333	GSE64857	GSM1581699	Cancer	NA	NA	NA	NA	NA
2334	GSE64857	GSM1581700	Cancer	NA	NA	NA	NA	NA
2335	GSE64857	GSM1581701	Cancer	NA	NA	NA	NA	NA
2336	GSE64857	GSM1581702	Cancer	NA	NA	NA	NA	NA
2337	GSE64857	GSM1581703	Cancer	NA	NA	NA	NA	NA

2338	GSE64857	GSM1581704	Cancer	NA	NA	NA	NA	NA
2339	GSE64857	GSM1581705	Cancer	NA	NA	NA	NA	NA
2340	GSE64857	GSM1581706	Cancer	NA	NA	NA	NA	NA
2341	GSE64857	GSM1581707	Cancer	NA	NA	NA	NA	NA
2342	GSE64857	GSM1581708	Cancer	NA	NA	NA	NA	NA
2343	GSE64857	GSM1581710	Cancer	NA	NA	NA	NA	NA
2344	GSE64857	GSM1581712	Cancer	NA	NA	NA	NA	NA
2345	GSE64857	GSM1581713	Cancer	NA	NA	NA	NA	NA
2346	GSE64857	GSM1581714	Cancer	NA	NA	NA	NA	NA
2347	GSE64857	GSM1581715	Cancer	NA	NA	NA	NA	NA
2348	GSE64857	GSM1581716	Cancer	NA	NA	NA	NA	NA
2349	GSE64857	GSM1581717	Cancer	NA	NA	NA	NA	NA
2350	GSE64857	GSM1581718	Cancer	NA	NA	NA	NA	NA
2351	GSE64857	GSM1581719	Cancer	NA	NA	NA	NA	NA
2352	GSE64857	GSM1581720	Cancer	NA	NA	NA	NA	NA
2353	GSE64857	GSM1581721	Cancer	NA	NA	NA	NA	NA
2354	GSE64857	GSM1581722	Cancer	NA	NA	NA	NA	NA
2355	GSE64857	GSM1581723	Cancer	NA	NA	NA	NA	NA
2356	GSE64857	GSM1581724	Cancer	NA	NA	NA	NA	NA
2357	GSE64857	GSM1581725	Cancer	NA	NA	NA	NA	NA
2358	GSE64857	GSM1581726	Cancer	NA	NA	NA	NA	NA
2359	GSE64857	GSM1581727	Cancer	NA	NA	NA	NA	NA
2360	GSE64857	GSM1581728	Cancer	NA	NA	NA	NA	NA
2361	GSE64857	GSM1581729	Cancer	NA	NA	NA	NA	NA
2362	GSE64857	GSM1581730	Cancer	NA	NA	NA	NA	NA
2363	GSE64857	GSM1581731	Cancer	NA	NA	NA	NA	NA
2364	GSE64857	GSM1581732	Cancer	NA	NA	NA	NA	NA
2365	GSE64857	GSM1581733	Cancer	NA	NA	NA	NA	NA
2366	GSE64857	GSM1581734	Cancer	NA	NA	NA	NA	NA
2367	GSE64857	GSM1581735	Cancer	NA	NA	NA	NA	NA
2368	GSE64857	GSM1581736	Cancer	NA	NA	NA	NA	NA
2369	GSE64857	GSM1581737	Cancer	NA	NA	NA	NA	NA
2370	GSE64857	GSM1581738	Cancer	NA	NA	NA	NA	NA
2371	GSE64857	GSM1581739	Cancer	NA	NA	NA	NA	NA
2372	GSE64857	GSM1581740	Cancer	NA	NA	NA	NA	NA
2373	GSE64857	GSM1581741	Cancer	NA	NA	NA	NA	NA
2374	GSE64857	GSM1581742	Cancer	NA	NA	NA	NA	NA
2375	GSE64857	GSM1581743	Cancer	NA	NA	NA	NA	NA
2376	GSE64857	GSM1581744	Cancer	NA	NA	NA	NA	NA
2377	GSE64857	GSM1581745	Cancer	NA	NA	NA	NA	NA
2378	GSE64857	GSM1581746	Cancer	NA	NA	NA	NA	NA
2379	GSE64857	GSM1581747	Cancer	NA	NA	NA	NA	NA
2380	GSE64857	GSM1581748	Cancer	NA	NA	NA	NA	NA
2381	GSE64857	GSM1581749	Cancer	NA	NA	NA	NA	NA
2382	GSE64857	GSM1581750	Cancer	NA	NA	NA	NA	NA

2383	GSE64857	GSM1581751	Cancer	NA	NA	NA	NA	NA
2384	GSE64857	GSM1581752	Cancer	NA	NA	NA	NA	NA
2385	GSE64857	GSM1581753	Cancer	NA	NA	NA	NA	NA
2386	GSE64857	GSM1581754	Cancer	NA	NA	NA	NA	NA
2387	GSE64857	GSM1581755	Cancer	NA	NA	NA	NA	NA
2388	GSE64857	GSM1581756	Cancer	NA	NA	NA	NA	NA
2389	GSE64857	GSM1581757	Cancer	NA	NA	NA	NA	NA
2390	GSE64857	GSM1581758	Cancer	NA	NA	NA	NA	NA
2391	GSE64857	GSM1581759	Cancer	NA	NA	NA	NA	NA
2392	GSE64857	GSM1581760	Cancer	NA	NA	NA	NA	NA
2393	GSE64857	GSM1581761	Cancer	NA	NA	NA	NA	NA
2394	GSE64857	GSM1581762	Cancer	NA	NA	NA	NA	NA
2395	GSE64857	GSM1581763	Cancer	NA	NA	NA	NA	NA
2396	GSE64857	GSM1581764	Cancer	NA	NA	NA	NA	NA
2397	GSE65222	GSM1590287	Cancer	NA	NA	NA	NA	NA
2398	GSE65222	GSM1590288	Cancer	NA	NA	NA	NA	NA
2399	GSE65222	GSM1590289	Cancer	NA	NA	NA	NA	NA
2400	GSE65222	GSM1590290	Cancer	NA	NA	NA	NA	NA
2401	GSE65222	GSM1590291	Cancer	NA	NA	NA	NA	NA
2402	GSE65222	GSM1590292	Cancer	NA	NA	NA	NA	NA
2403	GSE69657	GSM1705389	Cancer	NA	NA	NA	NA	NA
2404	GSE69657	GSM1705390	Cancer	NA	NA	NA	NA	NA
2405	GSE69657	GSM1705391	Cancer	NA	NA	NA	NA	NA
2406	GSE69657	GSM1705392	Cancer	NA	NA	NA	NA	NA
2407	GSE69657	GSM1705393	Cancer	NA	NA	NA	NA	NA
2408	GSE69657	GSM1705397	Cancer	NA	NA	NA	NA	NA
2409	GSE69657	GSM1705400	Cancer	NA	NA	NA	NA	NA
2410	GSE69657	GSM1705401	Cancer	NA	NA	NA	NA	NA
2411	GSE69657	GSM1705403	Cancer	NA	NA	NA	NA	NA
2412	GSE69657	GSM1705404	Cancer	NA	NA	NA	NA	NA
2413	GSE9348	GSM237914	Cancer	NA	NA	NA	NA	NA
2414	GSE9348	GSM237915	Cancer	NA	NA	NA	NA	NA
2415	GSE9348	GSM237916	Cancer	NA	NA	NA	NA	NA
2416	GSE9348	GSM237917	Cancer	NA	NA	NA	NA	NA
2417	GSE9348	GSM237918	Cancer	NA	NA	NA	NA	NA
2418	GSE9348	GSM237919	Cancer	NA	NA	NA	NA	NA
2419	GSE9348	GSM237920	Cancer	NA	NA	NA	NA	NA
2420	GSE9348	GSM237921	Cancer	NA	NA	NA	NA	NA
2421	GSE9348	GSM237922	Cancer	NA	NA	NA	NA	NA
2422	GSE9348	GSM237923	Cancer	NA	NA	NA	NA	NA
2423	GSE9348	GSM237924	Cancer	NA	NA	NA	NA	NA
2424	GSE9348	GSM237925	Cancer	NA	NA	NA	NA	NA
2425	GSE9348	GSM237926	Cancer	NA	NA	NA	NA	NA
2426	GSE9348	GSM237927	Cancer	NA	NA	NA	NA	NA
2427	GSE9348	GSM237928	Cancer	NA	NA	NA	NA	NA

2428	GSE9348	GSM237929	Cancer	NA	NA	NA	NA	NA
2429	GSE9348	GSM237930	Cancer	NA	NA	NA	NA	NA
2430	GSE9348	GSM237931	Cancer	NA	NA	NA	NA	NA
2431	GSE9348	GSM237932	Cancer	NA	NA	NA	NA	NA
2432	GSE9348	GSM237933	Cancer	NA	NA	NA	NA	NA
2433	GSE9348	GSM237934	Cancer	NA	NA	NA	NA	NA
2434	GSE9348	GSM237935	Cancer	NA	NA	NA	NA	NA
2435	GSE9348	GSM237936	Cancer	NA	NA	NA	NA	NA
2436	GSE9348	GSM237937	Cancer	NA	NA	NA	NA	NA
2437	GSE9348	GSM237938	Cancer	NA	NA	NA	NA	NA
2438	GSE9348	GSM237939	Cancer	NA	NA	NA	NA	NA
2439	GSE9348	GSM237940	Cancer	NA	NA	NA	NA	NA
2440	GSE9348	GSM237941	Cancer	NA	NA	NA	NA	NA
2441	GSE9348	GSM237942	Cancer	NA	NA	NA	NA	NA
2442	GSE9348	GSM237943	Cancer	NA	NA	NA	NA	NA
2443	GSE9348	GSM237945	Cancer	NA	NA	NA	NA	NA
2444	GSE9348	GSM237946	Cancer	NA	NA	NA	NA	NA
2445	GSE9348	GSM237947	Cancer	NA	NA	NA	NA	NA
2446	GSE9348	GSM237949	Cancer	NA	NA	NA	NA	NA
2447	GSE9348	GSM237950	Cancer	NA	NA	NA	NA	NA
2448	GSE9348	GSM237951	Cancer	NA	NA	NA	NA	NA
2449	GSE9348	GSM237952	Cancer	NA	NA	NA	NA	NA
2450	GSE9348	GSM237953	Cancer	NA	NA	NA	NA	NA
2451	GSE9348	GSM237954	Cancer	NA	NA	NA	NA	NA
2452	GSE9348	GSM237955	Cancer	NA	NA	NA	NA	NA
2453	GSE9348	GSM237956	Cancer	NA	NA	NA	NA	NA
2454	GSE9348	GSM237957	Cancer	NA	NA	NA	NA	NA
2455	GSE9348	GSM237958	Cancer	NA	NA	NA	NA	NA
2456	GSE9348	GSM237959	Cancer	NA	NA	NA	NA	NA
2457	GSE9348	GSM237960	Cancer	NA	NA	NA	NA	NA
2458	GSE9348	GSM237961	Cancer	NA	NA	NA	NA	NA
2459	GSE9348	GSM237962	Cancer	NA	NA	NA	NA	NA
2460	GSE9348	GSM237963	Cancer	NA	NA	NA	NA	NA
2461	GSE9348	GSM237964	Cancer	NA	NA	NA	NA	NA
2462	GSE9348	GSM237965	Cancer	NA	NA	NA	NA	NA
2463	GSE9348	GSM237966	Cancer	NA	NA	NA	NA	NA
2464	GSE9348	GSM237967	Cancer	NA	NA	NA	NA	NA
2465	GSE9348	GSM237968	Cancer	NA	NA	NA	NA	NA
2466	GSE9348	GSM237969	Cancer	NA	NA	NA	NA	NA
2467	GSE9348	GSM237970	Cancer	NA	NA	NA	NA	NA
2468	GSE9348	GSM237971	Cancer	NA	NA	NA	NA	NA
2469	GSE9348	GSM237972	Cancer	NA	NA	NA	NA	NA
2470	GSE9348	GSM237973	Cancer	NA	NA	NA	NA	NA
2471	GSE9348	GSM237974	Cancer	NA	NA	NA	NA	NA
2472	GSE9348	GSM237975	Cancer	NA	NA	NA	NA	NA

2473	GSE9348	GSM237976	Cancer	NA	NA	NA	NA	NA
2474	GSE9348	GSM237977	Cancer	NA	NA	NA	NA	NA
2475	GSE9348	GSM237978	Cancer	NA	NA	NA	NA	NA
2476	GSE9348	GSM237979	Cancer	NA	NA	NA	NA	NA
2477	GSE9348	GSM237980	Cancer	NA	NA	NA	NA	NA
2478	GSE9348	GSM237981	Cancer	NA	NA	NA	NA	NA
2479	GSE9348	GSM237982	Cancer	NA	NA	NA	NA	NA
2480	GSE9348	GSM237983	Cancer	NA	NA	NA	NA	NA
2481	GSE10616	GSM267527	IBDs	NA	NA	NA	NA	NA
2482	GSE10616	GSM267528	IBDs	NA	NA	NA	NA	NA
2483	GSE10616	GSM267530	IBDs	NA	NA	NA	NA	NA
2484	GSE10616	GSM267531	IBDs	NA	NA	NA	NA	NA
2485	GSE10616	GSM267532	IBDs	NA	NA	NA	NA	NA
2486	GSE10616	GSM267533	IBDs	NA	NA	NA	NA	NA
2487	GSE10616	GSM267534	IBDs	NA	NA	NA	NA	NA
2488	GSE10616	GSM267535	IBDs	NA	NA	NA	NA	NA
2489	GSE10616	GSM267536	IBDs	NA	NA	NA	NA	NA
2490	GSE10616	GSM267537	IBDs	NA	NA	NA	NA	NA
2491	GSE10616	GSM267538	IBDs	NA	NA	NA	NA	NA
2492	GSE10616	GSM267539	IBDs	NA	NA	NA	NA	NA
2493	GSE10616	GSM267540	IBDs	NA	NA	NA	NA	NA
2494	GSE10616	GSM267541	IBDs	NA	NA	NA	NA	NA
2495	GSE10616	GSM267542	IBDs	NA	NA	NA	NA	NA
2496	GSE10616	GSM267543	IBDs	NA	NA	NA	NA	NA
2497	GSE10616	GSM267544	IBDs	NA	NA	NA	NA	NA
2498	GSE10616	GSM267545	IBDs	NA	NA	NA	NA	NA
2499	GSE10616	GSM267546	IBDs	NA	NA	NA	NA	NA
2500	GSE10616	GSM267547	IBDs	NA	NA	NA	NA	NA
2501	GSE10616	GSM267548	IBDs	NA	NA	NA	NA	NA
2502	GSE10616	GSM267549	IBDs	NA	NA	NA	NA	NA
2503	GSE10616	GSM267550	IBDs	NA	NA	NA	NA	NA
2504	GSE10616	GSM267551	IBDs	NA	NA	NA	NA	NA
2505	GSE10616	GSM267552	IBDs	NA	NA	NA	NA	NA
2506	GSE10616	GSM267553	IBDs	NA	NA	NA	NA	NA
2507	GSE10616	GSM267554	IBDs	NA	NA	NA	NA	NA
2508	GSE10616	GSM267555	IBDs	NA	NA	NA	NA	NA
2509	GSE10616	GSM267556	IBDs	NA	NA	NA	NA	NA
2510	GSE10616	GSM267557	IBDs	NA	NA	NA	NA	NA
2511	GSE10616	GSM267558	IBDs	NA	NA	NA	NA	NA
2512	GSE10616	GSM267575	IBDs	NA	NA	NA	NA	NA
2513	GSE10616	GSM267576	IBDs	NA	NA	NA	NA	NA
2514	GSE10616	GSM267577	IBDs	NA	NA	NA	NA	NA
2515	GSE10616	GSM267578	IBDs	NA	NA	NA	NA	NA
2516	GSE10616	GSM267579	IBDs	NA	NA	NA	NA	NA
2517	GSE10616	GSM267580	IBDs	NA	NA	NA	NA	NA

2518	GSE10616	GSM267581	IBDs	NA	NA	NA	NA	NA
2519	GSE10616	GSM267582	IBDs	NA	NA	NA	NA	NA
2520	GSE10616	GSM267583	IBDs	NA	NA	NA	NA	NA
2521	GSE10616	GSM267584	IBDs	NA	NA	NA	NA	NA
2522	GSE10714	GSM270786	IBDs	NA	NA	NA	NA	NA
2523	GSE10714	GSM270787	IBDs	NA	NA	NA	NA	NA
2524	GSE10714	GSM270788	IBDs	NA	NA	NA	NA	NA
2525	GSE10714	GSM270789	IBDs	NA	NA	NA	NA	NA
2526	GSE10714	GSM270811	IBDs	NA	NA	NA	NA	NA
2527	GSE10714	GSM270812	IBDs	NA	NA	NA	NA	NA
2528	GSE10714	GSM270813	IBDs	NA	NA	NA	NA	NA
2529	GSE10791	GSM272419	IBDs	NA	NA	NA	NA	NA
2530	GSE13367	GSM337490	IBDs	NA	NA	NA	NA	NA
2531	GSE13367	GSM337491	IBDs	NA	NA	NA	NA	NA
2532	GSE13367	GSM337494	IBDs	NA	NA	NA	NA	NA
2533	GSE13367	GSM337495	IBDs	NA	NA	NA	NA	NA
2534	GSE13367	GSM337496	IBDs	NA	NA	NA	NA	NA
2535	GSE13367	GSM337500	IBDs	NA	NA	NA	NA	NA
2536	GSE13367	GSM337503	IBDs	NA	NA	NA	NA	NA
2537	GSE13367	GSM337513	IBDs	NA	NA	NA	NA	NA
2538	GSE13367	GSM337515	IBDs	NA	NA	NA	NA	NA
2539	GSE16879	GSM364633	IBDs	NA	NA	NA	NA	NA
2540	GSE16879	GSM364634	IBDs	NA	NA	NA	NA	NA
2541	GSE16879	GSM364635	IBDs	NA	NA	NA	NA	NA
2542	GSE16879	GSM364636	IBDs	NA	NA	NA	NA	NA
2543	GSE16879	GSM364637	IBDs	NA	NA	NA	NA	NA
2544	GSE16879	GSM364638	IBDs	NA	NA	NA	NA	NA
2545	GSE16879	GSM364639	IBDs	NA	NA	NA	NA	NA
2546	GSE16879	GSM364640	IBDs	NA	NA	NA	NA	NA
2547	GSE16879	GSM364641	IBDs	NA	NA	NA	NA	NA
2548	GSE16879	GSM364642	IBDs	NA	NA	NA	NA	NA
2549	GSE16879	GSM364643	IBDs	NA	NA	NA	NA	NA
2550	GSE16879	GSM364644	IBDs	NA	NA	NA	NA	NA
2551	GSE16879	GSM364645	IBDs	NA	NA	NA	NA	NA
2552	GSE16879	GSM364646	IBDs	NA	NA	NA	NA	NA
2553	GSE16879	GSM364647	IBDs	NA	NA	NA	NA	NA
2554	GSE16879	GSM364648	IBDs	NA	NA	NA	NA	NA
2555	GSE16879	GSM364649	IBDs	NA	NA	NA	NA	NA
2556	GSE16879	GSM364650	IBDs	NA	NA	NA	NA	NA
2557	GSE16879	GSM364651	IBDs	NA	NA	NA	NA	NA
2558	GSE16879	GSM364652	IBDs	NA	NA	NA	NA	NA
2559	GSE16879	GSM364653	IBDs	NA	NA	NA	NA	NA
2560	GSE16879	GSM364654	IBDs	NA	NA	NA	NA	NA
2561	GSE16879	GSM364655	IBDs	NA	NA	NA	NA	NA
2562	GSE16879	GSM364656	IBDs	NA	NA	NA	NA	NA

2563	GSE16879	GSM422963	IBDs	NA	NA	NA	NA	NA
2564	GSE16879	GSM422965	IBDs	NA	NA	NA	NA	NA
2565	GSE16879	GSM422967	IBDs	NA	NA	NA	NA	NA
2566	GSE16879	GSM422969	IBDs	NA	NA	NA	NA	NA
2567	GSE16879	GSM422971	IBDs	NA	NA	NA	NA	NA
2568	GSE16879	GSM422973	IBDs	NA	NA	NA	NA	NA
2569	GSE16879	GSM422977	IBDs	NA	NA	NA	NA	NA
2570	GSE16879	GSM422979	IBDs	NA	NA	NA	NA	NA
2571	GSE16879	GSM422981	IBDs	NA	NA	NA	NA	NA
2572	GSE16879	GSM422983	IBDs	NA	NA	NA	NA	NA
2573	GSE16879	GSM422985	IBDs	NA	NA	NA	NA	NA
2574	GSE16879	GSM422987	IBDs	NA	NA	NA	NA	NA
2575	GSE16879	GSM422989	IBDs	NA	NA	NA	NA	NA
2576	GSE16879	GSM422991	IBDs	NA	NA	NA	NA	NA
2577	GSE16879	GSM422993	IBDs	NA	NA	NA	NA	NA
2578	GSE16879	GSM422995	IBDs	NA	NA	NA	NA	NA
2579	GSE16879	GSM422997	IBDs	NA	NA	NA	NA	NA
2580	GSE16879	GSM422999	IBDs	NA	NA	NA	NA	NA
2581	GSE16879	GSM423001	IBDs	NA	NA	NA	NA	NA
2582	GSE16879	GSM423003	IBDs	NA	NA	NA	NA	NA
2583	GSE16879	GSM423005	IBDs	NA	NA	NA	NA	NA
2584	GSE16879	GSM423007	IBDs	NA	NA	NA	NA	NA
2585	GSE16879	GSM423009	IBDs	NA	NA	NA	NA	NA
2586	GSE16879	GSM423010	IBDs	NA	NA	NA	NA	NA
2587	GSE16879	GSM423011	IBDs	NA	NA	NA	NA	NA
2588	GSE16879	GSM423012	IBDs	NA	NA	NA	NA	NA
2589	GSE16879	GSM423013	IBDs	NA	NA	NA	NA	NA
2590	GSE16879	GSM423014	IBDs	NA	NA	NA	NA	NA
2591	GSE16879	GSM423015	IBDs	NA	NA	NA	NA	NA
2592	GSE16879	GSM423016	IBDs	NA	NA	NA	NA	NA
2593	GSE16879	GSM423017	IBDs	NA	NA	NA	NA	NA
2594	GSE16879	GSM423018	IBDs	NA	NA	NA	NA	NA
2595	GSE16879	GSM423019	IBDs	NA	NA	NA	NA	NA
2596	GSE16879	GSM423020	IBDs	NA	NA	NA	NA	NA
2597	GSE16879	GSM423021	IBDs	NA	NA	NA	NA	NA
2598	GSE16879	GSM423022	IBDs	NA	NA	NA	NA	NA
2599	GSE16879	GSM423023	IBDs	NA	NA	NA	NA	NA
2600	GSE16879	GSM423024	IBDs	NA	NA	NA	NA	NA
2601	GSE16879	GSM423025	IBDs	NA	NA	NA	NA	NA
2602	GSE16879	GSM423026	IBDs	NA	NA	NA	NA	NA
2603	GSE16879	GSM423027	IBDs	NA	NA	NA	NA	NA
2604	GSE16879	GSM423028	IBDs	NA	NA	NA	NA	NA
2605	GSE16879	GSM423029	IBDs	NA	NA	NA	NA	NA
2606	GSE16879	GSM423030	IBDs	NA	NA	NA	NA	NA
2607	GSE16879	GSM423031	IBDs	NA	NA	NA	NA	NA

2608	GSE16879	GSM423032	IBDs	NA	NA	NA	NA	NA
2609	GSE16879	GSM423033	IBDs	NA	NA	NA	NA	NA
2610	GSE16879	GSM423034	IBDs	NA	NA	NA	NA	NA
2611	GSE16879	GSM423035	IBDs	NA	NA	NA	NA	NA
2612	GSE16879	GSM423036	IBDs	NA	NA	NA	NA	NA
2613	GSE16879	GSM423037	IBDs	NA	NA	NA	NA	NA
2614	GSE16879	GSM423038	IBDs	NA	NA	NA	NA	NA
2615	GSE16879	GSM423039	IBDs	NA	NA	NA	NA	NA
2616	GSE16879	GSM423040	IBDs	NA	NA	NA	NA	NA
2617	GSE16879	GSM423041	IBDs	NA	NA	NA	NA	NA
2618	GSE16879	GSM423042	IBDs	NA	NA	NA	NA	NA
2619	GSE16879	GSM423043	IBDs	NA	NA	NA	NA	NA
2620	GSE16879	GSM423044	IBDs	NA	NA	NA	NA	NA
2621	GSE16879	GSM423045	IBDs	NA	NA	NA	NA	NA
2622	GSE16879	GSM423046	IBDs	NA	NA	NA	NA	NA
2623	GSE16879	GSM423047	IBDs	NA	NA	NA	NA	NA
2624	GSE16879	GSM423048	IBDs	NA	NA	NA	NA	NA
2625	GSE16879	GSM423049	IBDs	NA	NA	NA	NA	NA
2626	GSE16879	GSM423050	IBDs	NA	NA	NA	NA	NA
2627	GSE16879	GSM423051	IBDs	NA	NA	NA	NA	NA
2628	GSE16879	GSM423052	IBDs	NA	NA	NA	NA	NA
2629	GSE16879	GSM423053	IBDs	NA	NA	NA	NA	NA
2630	GSE16879	GSM423054	IBDs	NA	NA	NA	NA	NA
2631	GSE16879	GSM423055	IBDs	NA	NA	NA	NA	NA
2632	GSE16879	GSM423056	IBDs	NA	NA	NA	NA	NA
2633	GSE16879	GSM423057	IBDs	NA	NA	NA	NA	NA
2634	GSE16879	GSM423058	IBDs	NA	NA	NA	NA	NA
2635	GSE16879	GSM423059	IBDs	NA	NA	NA	NA	NA
2636	GSE16879	GSM423060	IBDs	NA	NA	NA	NA	NA
2637	GSE16879	GSM423061	IBDs	NA	NA	NA	NA	NA
2638	GSE16879	GSM423062	IBDs	NA	NA	NA	NA	NA
2639	GSE16879	GSM423063	IBDs	NA	NA	NA	NA	NA
2640	GSE16879	GSM423064	IBDs	NA	NA	NA	NA	NA
2641	GSE16879	GSM423065	IBDs	NA	NA	NA	NA	NA
2642	GSE16879	GSM423066	IBDs	NA	NA	NA	NA	NA
2643	GSE16879	GSM423067	IBDs	NA	NA	NA	NA	NA
2644	GSE16879	GSM423068	IBDs	NA	NA	NA	NA	NA
2645	GSE16879	GSM423069	IBDs	NA	NA	NA	NA	NA
2646	GSE16879	GSM423070	IBDs	NA	NA	NA	NA	NA
2647	GSE16879	GSM423071	IBDs	NA	NA	NA	NA	NA
2648	GSE16879	GSM423072	IBDs	NA	NA	NA	NA	NA
2649	GSE16879	GSM423073	IBDs	NA	NA	NA	NA	NA
2650	GSE16879	GSM423074	IBDs	NA	NA	NA	NA	NA
2651	GSE16879	GSM423075	IBDs	NA	NA	NA	NA	NA
2652	GSE16879	GSM423076	IBDs	NA	NA	NA	NA	NA

2653	GSE16879	GSM423077	IBDs	NA	NA	NA	NA	NA
2654	GSE16879	GSM423078	IBDs	NA	NA	NA	NA	NA
2655	GSE16879	GSM423079	IBDs	NA	NA	NA	NA	NA
2656	GSE16879	GSM423080	IBDs	NA	NA	NA	NA	NA
2657	GSE16879	GSM423081	IBDs	NA	NA	NA	NA	NA
2658	GSE16879	GSM423082	IBDs	NA	NA	NA	NA	NA
2659	GSE16879	GSM423083	IBDs	NA	NA	NA	NA	NA
2660	GSE16879	GSM423084	IBDs	NA	NA	NA	NA	NA
2661	GSE16879	GSM423085	IBDs	NA	NA	NA	NA	NA
2662	GSE16879	GSM423086	IBDs	NA	NA	NA	NA	NA
2663	GSE16879	GSM423087	IBDs	NA	NA	NA	NA	NA
2664	GSE16879	GSM423088	IBDs	NA	NA	NA	NA	NA
2665	GSE22619	GSM1012178	IBDs	NA	NA	NA	NA	NA
2666	GSE22619	GSM1012180	IBDs	NA	NA	NA	NA	NA
2667	GSE22619	GSM560969	IBDs	NA	NA	NA	NA	NA
2668	GSE22619	GSM560970	IBDs	NA	NA	NA	NA	NA
2669	GSE22619	GSM560971	IBDs	NA	NA	NA	NA	NA
2670	GSE22619	GSM560972	IBDs	NA	NA	NA	NA	NA
2671	GSE22619	GSM560973	IBDs	NA	NA	NA	NA	NA
2672	GSE22619	GSM560974	IBDs	NA	NA	NA	NA	NA
2673	GSE22619	GSM560975	IBDs	NA	NA	NA	NA	NA
2674	GSE22619	GSM560976	IBDs	NA	NA	NA	NA	NA
2675	GSE23597	GSM578713	IBDs	NA	NA	NA	NA	NA
2676	GSE23597	GSM578714	IBDs	NA	NA	NA	NA	NA
2677	GSE23597	GSM578715	IBDs	NA	NA	NA	NA	NA
2678	GSE23597	GSM578716	IBDs	NA	NA	NA	NA	NA
2679	GSE23597	GSM578717	IBDs	NA	NA	NA	NA	NA
2680	GSE23597	GSM578718	IBDs	NA	NA	NA	NA	NA
2681	GSE23597	GSM578719	IBDs	NA	NA	NA	NA	NA
2682	GSE23597	GSM578720	IBDs	NA	NA	NA	NA	NA
2683	GSE23597	GSM578721	IBDs	NA	NA	NA	NA	NA
2684	GSE23597	GSM578722	IBDs	NA	NA	NA	NA	NA
2685	GSE23597	GSM578723	IBDs	NA	NA	NA	NA	NA
2686	GSE23597	GSM578724	IBDs	NA	NA	NA	NA	NA
2687	GSE23597	GSM578725	IBDs	NA	NA	NA	NA	NA
2688	GSE23597	GSM578726	IBDs	NA	NA	NA	NA	NA
2689	GSE23597	GSM578727	IBDs	NA	NA	NA	NA	NA
2690	GSE23597	GSM578728	IBDs	NA	NA	NA	NA	NA
2691	GSE23597	GSM578729	IBDs	NA	NA	NA	NA	NA
2692	GSE23597	GSM578730	IBDs	NA	NA	NA	NA	NA
2693	GSE23597	GSM578731	IBDs	NA	NA	NA	NA	NA
2694	GSE23597	GSM578732	IBDs	NA	NA	NA	NA	NA
2695	GSE23597	GSM578733	IBDs	NA	NA	NA	NA	NA
2696	GSE23597	GSM578734	IBDs	NA	NA	NA	NA	NA
2697	GSE23597	GSM578735	IBDs	NA	NA	NA	NA	NA

2698	GSE23597	GSM578736	IBDs	NA	NA	NA	NA	NA
2699	GSE23597	GSM578737	IBDs	NA	NA	NA	NA	NA
2700	GSE23597	GSM578738	IBDs	NA	NA	NA	NA	NA
2701	GSE23597	GSM578739	IBDs	NA	NA	NA	NA	NA
2702	GSE23597	GSM578740	IBDs	NA	NA	NA	NA	NA
2703	GSE23597	GSM578741	IBDs	NA	NA	NA	NA	NA
2704	GSE23597	GSM578742	IBDs	NA	NA	NA	NA	NA
2705	GSE23597	GSM578743	IBDs	NA	NA	NA	NA	NA
2706	GSE23597	GSM578744	IBDs	NA	NA	NA	NA	NA
2707	GSE23597	GSM578745	IBDs	NA	NA	NA	NA	NA
2708	GSE23597	GSM578746	IBDs	NA	NA	NA	NA	NA
2709	GSE23597	GSM578747	IBDs	NA	NA	NA	NA	NA
2710	GSE23597	GSM578748	IBDs	NA	NA	NA	NA	NA
2711	GSE23597	GSM578749	IBDs	NA	NA	NA	NA	NA
2712	GSE23597	GSM578750	IBDs	NA	NA	NA	NA	NA
2713	GSE23597	GSM578751	IBDs	NA	NA	NA	NA	NA
2714	GSE23597	GSM578752	IBDs	NA	NA	NA	NA	NA
2715	GSE23597	GSM578753	IBDs	NA	NA	NA	NA	NA
2716	GSE23597	GSM578754	IBDs	NA	NA	NA	NA	NA
2717	GSE23597	GSM578755	IBDs	NA	NA	NA	NA	NA
2718	GSE23597	GSM578756	IBDs	NA	NA	NA	NA	NA
2719	GSE23597	GSM578758	IBDs	NA	NA	NA	NA	NA
2720	GSE23597	GSM578759	IBDs	NA	NA	NA	NA	NA
2721	GSE23597	GSM578760	IBDs	NA	NA	NA	NA	NA
2722	GSE23597	GSM578761	IBDs	NA	NA	NA	NA	NA
2723	GSE23597	GSM578762	IBDs	NA	NA	NA	NA	NA
2724	GSE23597	GSM578763	IBDs	NA	NA	NA	NA	NA
2725	GSE23597	GSM578764	IBDs	NA	NA	NA	NA	NA
2726	GSE23597	GSM578765	IBDs	NA	NA	NA	NA	NA
2727	GSE23597	GSM578766	IBDs	NA	NA	NA	NA	NA
2728	GSE23597	GSM578767	IBDs	NA	NA	NA	NA	NA
2729	GSE23597	GSM578768	IBDs	NA	NA	NA	NA	NA
2730	GSE23597	GSM578769	IBDs	NA	NA	NA	NA	NA
2731	GSE23597	GSM578770	IBDs	NA	NA	NA	NA	NA
2732	GSE23597	GSM578771	IBDs	NA	NA	NA	NA	NA
2733	GSE23597	GSM578772	IBDs	NA	NA	NA	NA	NA
2734	GSE23597	GSM578773	IBDs	NA	NA	NA	NA	NA
2735	GSE23597	GSM578774	IBDs	NA	NA	NA	NA	NA
2736	GSE23597	GSM578775	IBDs	NA	NA	NA	NA	NA
2737	GSE23597	GSM578776	IBDs	NA	NA	NA	NA	NA
2738	GSE23597	GSM578777	IBDs	NA	NA	NA	NA	NA
2739	GSE23597	GSM578778	IBDs	NA	NA	NA	NA	NA
2740	GSE23597	GSM578779	IBDs	NA	NA	NA	NA	NA
2741	GSE23597	GSM578780	IBDs	NA	NA	NA	NA	NA
2742	GSE23597	GSM578781	IBDs	NA	NA	NA	NA	NA

2743	GSE23597	GSM578782	IBDs	NA	NA	NA	NA	NA
2744	GSE23597	GSM578783	IBDs	NA	NA	NA	NA	NA
2745	GSE23597	GSM578784	IBDs	NA	NA	NA	NA	NA
2746	GSE23597	GSM578785	IBDs	NA	NA	NA	NA	NA
2747	GSE23597	GSM578786	IBDs	NA	NA	NA	NA	NA
2748	GSE23597	GSM578787	IBDs	NA	NA	NA	NA	NA
2749	GSE23597	GSM578788	IBDs	NA	NA	NA	NA	NA
2750	GSE23597	GSM578789	IBDs	NA	NA	NA	NA	NA
2751	GSE23597	GSM578790	IBDs	NA	NA	NA	NA	NA
2752	GSE23597	GSM578791	IBDs	NA	NA	NA	NA	NA
2753	GSE23597	GSM578792	IBDs	NA	NA	NA	NA	NA
2754	GSE23597	GSM578793	IBDs	NA	NA	NA	NA	NA
2755	GSE23597	GSM578794	IBDs	NA	NA	NA	NA	NA
2756	GSE23597	GSM578795	IBDs	NA	NA	NA	NA	NA
2757	GSE23597	GSM578796	IBDs	NA	NA	NA	NA	NA
2758	GSE23597	GSM578797	IBDs	NA	NA	NA	NA	NA
2759	GSE23597	GSM578798	IBDs	NA	NA	NA	NA	NA
2760	GSE23597	GSM578799	IBDs	NA	NA	NA	NA	NA
2761	GSE23597	GSM578800	IBDs	NA	NA	NA	NA	NA
2762	GSE23597	GSM578801	IBDs	NA	NA	NA	NA	NA
2763	GSE23597	GSM578802	IBDs	NA	NA	NA	NA	NA
2764	GSE23597	GSM578803	IBDs	NA	NA	NA	NA	NA
2765	GSE23597	GSM578804	IBDs	NA	NA	NA	NA	NA
2766	GSE23597	GSM578805	IBDs	NA	NA	NA	NA	NA
2767	GSE23597	GSM578806	IBDs	NA	NA	NA	NA	NA
2768	GSE23597	GSM578807	IBDs	NA	NA	NA	NA	NA
2769	GSE23597	GSM578808	IBDs	NA	NA	NA	NA	NA
2770	GSE23597	GSM578809	IBDs	NA	NA	NA	NA	NA
2771	GSE23597	GSM578810	IBDs	NA	NA	NA	NA	NA
2772	GSE23597	GSM578811	IBDs	NA	NA	NA	NA	NA
2773	GSE23597	GSM578812	IBDs	NA	NA	NA	NA	NA
2774	GSE23597	GSM578813	IBDs	NA	NA	NA	NA	NA
2775	GSE23597	GSM578814	IBDs	NA	NA	NA	NA	NA
2776	GSE23597	GSM578815	IBDs	NA	NA	NA	NA	NA
2777	GSE23597	GSM578816	IBDs	NA	NA	NA	NA	NA
2778	GSE23597	GSM578817	IBDs	NA	NA	NA	NA	NA
2779	GSE23597	GSM578818	IBDs	NA	NA	NA	NA	NA
2780	GSE23597	GSM578819	IBDs	NA	NA	NA	NA	NA
2781	GSE23597	GSM578820	IBDs	NA	NA	NA	NA	NA
2782	GSE23597	GSM578821	IBDs	NA	NA	NA	NA	NA
2783	GSE23597	GSM578822	IBDs	NA	NA	NA	NA	NA
2784	GSE23597	GSM578823	IBDs	NA	NA	NA	NA	NA
2785	GSE23597	GSM578824	IBDs	NA	NA	NA	NA	NA
2786	GSE23597	GSM578825	IBDs	NA	NA	NA	NA	NA
2787	GSE36807	GSM901326	IBDs	NA	NA	NA	NA	NA

2788	GSE36807	GSM901327	IBDs	NA	NA	NA	NA	NA
2789	GSE36807	GSM901328	IBDs	NA	NA	NA	NA	NA
2790	GSE36807	GSM901329	IBDs	NA	NA	NA	NA	NA
2791	GSE36807	GSM901330	IBDs	NA	NA	NA	NA	NA
2792	GSE36807	GSM901331	IBDs	NA	NA	NA	NA	NA
2793	GSE36807	GSM901332	IBDs	NA	NA	NA	NA	NA
2794	GSE36807	GSM901333	IBDs	NA	NA	NA	NA	NA
2795	GSE36807	GSM901334	IBDs	NA	NA	NA	NA	NA
2796	GSE36807	GSM901335	IBDs	NA	NA	NA	NA	NA
2797	GSE36807	GSM901336	IBDs	NA	NA	NA	NA	NA
2798	GSE36807	GSM901337	IBDs	NA	NA	NA	NA	NA
2799	GSE36807	GSM901338	IBDs	NA	NA	NA	NA	NA
2800	GSE36807	GSM901340	IBDs	NA	NA	NA	NA	NA
2801	GSE36807	GSM901341	IBDs	NA	NA	NA	NA	NA
2802	GSE36807	GSM901342	IBDs	NA	NA	NA	NA	NA
2803	GSE36807	GSM901343	IBDs	NA	NA	NA	NA	NA
2804	GSE36807	GSM901344	IBDs	NA	NA	NA	NA	NA
2805	GSE36807	GSM901345	IBDs	NA	NA	NA	NA	NA
2806	GSE36807	GSM901346	IBDs	NA	NA	NA	NA	NA
2807	GSE36807	GSM901347	IBDs	NA	NA	NA	NA	NA
2808	GSE36807	GSM901348	IBDs	NA	NA	NA	NA	NA
2809	GSE36807	GSM901349	IBDs	NA	NA	NA	NA	NA
2810	GSE36807	GSM901350	IBDs	NA	NA	NA	NA	NA
2811	GSE36807	GSM901351	IBDs	NA	NA	NA	NA	NA
2812	GSE36807	GSM901352	IBDs	NA	NA	NA	NA	NA
2813	GSE36807	GSM901353	IBDs	NA	NA	NA	NA	NA
2814	GSE38713	GSM948563	IBDs	NA	NA	NA	NA	NA
2815	GSE38713	GSM948564	IBDs	NA	NA	NA	NA	NA
2816	GSE38713	GSM948565	IBDs	NA	NA	NA	NA	NA
2817	GSE38713	GSM948566	IBDs	NA	NA	NA	NA	NA
2818	GSE38713	GSM948567	IBDs	NA	NA	NA	NA	NA
2819	GSE38713	GSM948568	IBDs	NA	NA	NA	NA	NA
2820	GSE38713	GSM948569	IBDs	NA	NA	NA	NA	NA
2821	GSE38713	GSM948570	IBDs	NA	NA	NA	NA	NA
2822	GSE38713	GSM948571	IBDs	NA	NA	NA	NA	NA
2823	GSE38713	GSM948572	IBDs	NA	NA	NA	NA	NA
2824	GSE38713	GSM948573	IBDs	NA	NA	NA	NA	NA
2825	GSE38713	GSM948574	IBDs	NA	NA	NA	NA	NA
2826	GSE38713	GSM948575	IBDs	NA	NA	NA	NA	NA
2827	GSE38713	GSM948576	IBDs	NA	NA	NA	NA	NA
2828	GSE38713	GSM948577	IBDs	NA	NA	NA	NA	NA
2829	GSE38713	GSM948578	IBDs	NA	NA	NA	NA	NA
2830	GSE38713	GSM948579	IBDs	NA	NA	NA	NA	NA
2831	GSE38713	GSM948580	IBDs	NA	NA	NA	NA	NA
2832	GSE38713	GSM948581	IBDs	NA	NA	NA	NA	NA

2833	GSE38713	GSM948582	IBDs	NA	NA	NA	NA	NA
2834	GSE38713	GSM948583	IBDs	NA	NA	NA	NA	NA
2835	GSE38713	GSM948584	IBDs	NA	NA	NA	NA	NA
2836	GSE38713	GSM948585	IBDs	NA	NA	NA	NA	NA
2837	GSE38713	GSM948586	IBDs	NA	NA	NA	NA	NA
2838	GSE38713	GSM948587	IBDs	NA	NA	NA	NA	NA
2839	GSE38713	GSM948588	IBDs	NA	NA	NA	NA	NA
2840	GSE38713	GSM948589	IBDs	NA	NA	NA	NA	NA
2841	GSE38713	GSM948590	IBDs	NA	NA	NA	NA	NA
2842	GSE38713	GSM948591	IBDs	NA	NA	NA	NA	NA
2843	GSE38713	GSM948592	IBDs	NA	NA	NA	NA	NA
2844	GSE4183	GSM95511	IBDs	NA	NA	NA	NA	NA
2845	GSE4183	GSM95512	IBDs	NA	NA	NA	NA	NA
2846	GSE4183	GSM95514	IBDs	NA	NA	NA	NA	NA
2847	GSE4183	GSM95515	IBDs	NA	NA	NA	NA	NA
2848	GSE4183	GSM95516	IBDs	NA	NA	NA	NA	NA
2849	GSE4183	GSM95517	IBDs	NA	NA	NA	NA	NA
2850	GSE4183	GSM95518	IBDs	NA	NA	NA	NA	NA
2851	GSE4183	GSM95519	IBDs	NA	NA	NA	NA	NA
2852	GSE4183	GSM95520	IBDs	NA	NA	NA	NA	NA
2853	GSE4183	GSM95521	IBDs	NA	NA	NA	NA	NA
2854	GSE4183	GSM95522	IBDs	NA	NA	NA	NA	NA
2855	GSE4183	GSM95523	IBDs	NA	NA	NA	NA	NA
2856	GSE4183	GSM95524	IBDs	NA	NA	NA	NA	NA
2857	GSE4183	GSM95525	IBDs	NA	NA	NA	NA	NA
2858	GSE47908	GSM1162242	IBDs	NA	NA	NA	NA	NA
2859	GSE47908	GSM1162243	IBDs	NA	NA	NA	NA	NA
2860	GSE47908	GSM1162244	IBDs	NA	NA	NA	NA	NA
2861	GSE47908	GSM1162245	IBDs	NA	NA	NA	NA	NA
2862	GSE47908	GSM1162247	IBDs	NA	NA	NA	NA	NA
2863	GSE47908	GSM1162248	IBDs	NA	NA	NA	NA	NA
2864	GSE47908	GSM1162249	IBDs	NA	NA	NA	NA	NA
2865	GSE47908	GSM1162250	IBDs	NA	NA	NA	NA	NA
2866	GSE47908	GSM1162251	IBDs	NA	NA	NA	NA	NA
2867	GSE47908	GSM1162252	IBDs	NA	NA	NA	NA	NA
2868	GSE47908	GSM1162253	IBDs	NA	NA	NA	NA	NA
2869	GSE47908	GSM1162254	IBDs	NA	NA	NA	NA	NA
2870	GSE47908	GSM1162255	IBDs	NA	NA	NA	NA	NA
2871	GSE47908	GSM1162256	IBDs	NA	NA	NA	NA	NA
2872	GSE47908	GSM1162257	IBDs	NA	NA	NA	NA	NA
2873	GSE47908	GSM1162258	IBDs	NA	NA	NA	NA	NA
2874	GSE47908	GSM1162259	IBDs	NA	NA	NA	NA	NA
2875	GSE47908	GSM1162260	IBDs	NA	NA	NA	NA	NA
2876	GSE47908	GSM1162261	IBDs	NA	NA	NA	NA	NA
2877	GSE47908	GSM1162262	IBDs	NA	NA	NA	NA	NA

2878	GSE47908	GSM1162263	IBDs	NA	NA	NA	NA	NA
2879	GSE47908	GSM1162264	IBDs	NA	NA	NA	NA	NA
2880	GSE47908	GSM1162265	IBDs	NA	NA	NA	NA	NA
2881	GSE47908	GSM1162266	IBDs	NA	NA	NA	NA	NA
2882	GSE47908	GSM1162267	IBDs	NA	NA	NA	NA	NA
2883	GSE47908	GSM1162268	IBDs	NA	NA	NA	NA	NA
2884	GSE47908	GSM1162269	IBDs	NA	NA	NA	NA	NA
2885	GSE47908	GSM1162270	IBDs	NA	NA	NA	NA	NA
2886	GSE47908	GSM1162271	IBDs	NA	NA	NA	NA	NA
2887	GSE47908	GSM1162272	IBDs	NA	NA	NA	NA	NA
2888	GSE47908	GSM1162273	IBDs	NA	NA	NA	NA	NA
2889	GSE47908	GSM1162274	IBDs	NA	NA	NA	NA	NA
2890	GSE47908	GSM1162275	IBDs	NA	NA	NA	NA	NA
2891	GSE47908	GSM1162276	IBDs	NA	NA	NA	NA	NA
2892	GSE47908	GSM1162277	IBDs	NA	NA	NA	NA	NA
2893	GSE47908	GSM1162278	IBDs	NA	NA	NA	NA	NA
2894	GSE47908	GSM1162279	IBDs	NA	NA	NA	NA	NA
2895	GSE47908	GSM1162280	IBDs	NA	NA	NA	NA	NA
2896	GSE47908	GSM1162281	IBDs	NA	NA	NA	NA	NA
2897	GSE47908	GSM1162282	IBDs	NA	NA	NA	NA	NA
2898	GSE47908	GSM1162283	IBDs	NA	NA	NA	NA	NA
2899	GSE47908	GSM1162284	IBDs	NA	NA	NA	NA	NA
2900	GSE47908	GSM1162285	IBDs	NA	NA	NA	NA	NA
2901	GSE47908	GSM1162286	IBDs	NA	NA	NA	NA	NA
2902	GSE52746	GSM1275185	IBDs	NA	NA	NA	NA	NA
2903	GSE52746	GSM1275186	IBDs	NA	NA	NA	NA	NA
2904	GSE52746	GSM1275187	IBDs	NA	NA	NA	NA	NA
2905	GSE52746	GSM1275188	IBDs	NA	NA	NA	NA	NA
2906	GSE52746	GSM1275189	IBDs	NA	NA	NA	NA	NA
2907	GSE52746	GSM1275190	IBDs	NA	NA	NA	NA	NA
2908	GSE52746	GSM1275191	IBDs	NA	NA	NA	NA	NA
2909	GSE52746	GSM1275192	IBDs	NA	NA	NA	NA	NA
2910	GSE52746	GSM1275193	IBDs	NA	NA	NA	NA	NA
2911	GSE52746	GSM1275194	IBDs	NA	NA	NA	NA	NA
2912	GSE52746	GSM1275195	IBDs	NA	NA	NA	NA	NA
2913	GSE52746	GSM1275196	IBDs	NA	NA	NA	NA	NA
2914	GSE52746	GSM1275197	IBDs	NA	NA	NA	NA	NA
2915	GSE52746	GSM1275198	IBDs	NA	NA	NA	NA	NA
2916	GSE52746	GSM1275199	IBDs	NA	NA	NA	NA	NA
2917	GSE52746	GSM1275200	IBDs	NA	NA	NA	NA	NA
2918	GSE52746	GSM1275201	IBDs	NA	NA	NA	NA	NA
2919	GSE52746	GSM1275202	IBDs	NA	NA	NA	NA	NA
2920	GSE52746	GSM1275203	IBDs	NA	NA	NA	NA	NA
2921	GSE52746	GSM1275204	IBDs	NA	NA	NA	NA	NA
2922	GSE52746	GSM1275205	IBDs	NA	NA	NA	NA	NA

2923	GSE52746	GSM1275206	IBDs	NA	NA	NA	NA	NA
2924	GSE9452	GSM239617	IBDs	NA	NA	NA	NA	NA
2925	GSE9452	GSM239618	IBDs	NA	NA	NA	NA	NA
2926	GSE9452	GSM239714	IBDs	NA	NA	NA	NA	NA
2927	GSE9452	GSM239716	IBDs	NA	NA	NA	NA	NA
2928	GSE9452	GSM239717	IBDs	NA	NA	NA	NA	NA
2929	GSE9452	GSM239718	IBDs	NA	NA	NA	NA	NA
2930	GSE9452	GSM239719	IBDs	NA	NA	NA	NA	NA
2931	GSE9452	GSM239720	IBDs	NA	NA	NA	NA	NA
2932	GSE10616	GSM267559	Normal	NA	NA	NA	NA	NA
2933	GSE10616	GSM267560	Normal	NA	NA	NA	NA	NA
2934	GSE10616	GSM267561	Normal	NA	NA	NA	NA	NA
2935	GSE10616	GSM267562	Normal	NA	NA	NA	NA	NA
2936	GSE10616	GSM267563	Normal	NA	NA	NA	NA	NA
2937	GSE10616	GSM267564	Normal	NA	NA	NA	NA	NA
2938	GSE10616	GSM267565	Normal	NA	NA	NA	NA	NA
2939	GSE10616	GSM267566	Normal	NA	NA	NA	NA	NA
2940	GSE10616	GSM267567	Normal	NA	NA	NA	NA	NA
2941	GSE10616	GSM267568	Normal	NA	NA	NA	NA	NA
2942	GSE10616	GSM267569	Normal	NA	NA	NA	NA	NA
2943	GSE10616	GSM267570	Normal	NA	NA	NA	NA	NA
2944	GSE10616	GSM267571	Normal	NA	NA	NA	NA	NA
2945	GSE10616	GSM267572	Normal	NA	NA	NA	NA	NA
2946	GSE10616	GSM267573	Normal	NA	NA	NA	NA	NA
2947	GSE10616	GSM267574	Normal	NA	NA	NA	NA	NA
2948	GSE10714	GSM270808	Normal	NA	NA	NA	NA	NA
2949	GSE10714	GSM270809	Normal	NA	NA	NA	NA	NA
2950	GSE10714	GSM270810	Normal	NA	NA	NA	NA	NA
2951	GSE10791	GSM272418	Normal	NA	NA	NA	NA	NA
2952	GSE11831	GSM298776	Normal	NA	NA	NA	NA	NA
2953	GSE11831	GSM298778	Normal	NA	NA	NA	NA	NA
2954	GSE11831	GSM298782	Normal	NA	NA	NA	NA	NA
2955	GSE11831	GSM298789	Normal	NA	NA	NA	NA	NA
2956	GSE11831	GSM298798	Normal	NA	NA	NA	NA	NA
2957	GSE11831	GSM298947	Normal	NA	NA	NA	NA	NA
2958	GSE11831	GSM298948	Normal	NA	NA	NA	NA	NA
2959	GSE11831	GSM298950	Normal	NA	NA	NA	NA	NA
2960	GSE11831	GSM298951	Normal	NA	NA	NA	NA	NA
2961	GSE11831	GSM298952	Normal	NA	NA	NA	NA	NA
2962	GSE11831	GSM298953	Normal	NA	NA	NA	NA	NA
2963	GSE11831	GSM298956	Normal	NA	NA	NA	NA	NA
2964	GSE11831	GSM298958	Normal	NA	NA	NA	NA	NA
2965	GSE11831	GSM298961	Normal	NA	NA	NA	NA	NA
2966	GSE11831	GSM298962	Normal	NA	NA	NA	NA	NA
2967	GSE11831	GSM298964	Normal	NA	NA	NA	NA	NA

2968	GSE11831	GSM298967	Normal	NA	NA	NA	NA	NA
2969	GSE13367	GSM337492	Normal	NA	NA	NA	NA	NA
2970	GSE13367	GSM337497	Normal	NA	NA	NA	NA	NA
2971	GSE13367	GSM337498	Normal	NA	NA	NA	NA	NA
2972	GSE13367	GSM337499	Normal	NA	NA	NA	NA	NA
2973	GSE13367	GSM337501	Normal	NA	NA	NA	NA	NA
2974	GSE13367	GSM337502	Normal	NA	NA	NA	NA	NA
2975	GSE13367	GSM337504	Normal	NA	NA	NA	NA	NA
2976	GSE13367	GSM337505	Normal	NA	NA	NA	NA	NA
2977	GSE13367	GSM337506	Normal	NA	NA	NA	NA	NA
2978	GSE13367	GSM337507	Normal	NA	NA	NA	NA	NA
2979	GSE13367	GSM337508	Normal	NA	NA	NA	NA	NA
2980	GSE13367	GSM337509	Normal	NA	NA	NA	NA	NA
2981	GSE13367	GSM337510	Normal	NA	NA	NA	NA	NA
2982	GSE13367	GSM337511	Normal	NA	NA	NA	NA	NA
2983	GSE13367	GSM337512	Normal	NA	NA	NA	NA	NA
2984	GSE13367	GSM337514	Normal	NA	NA	NA	NA	NA
2985	GSE13367	GSM337516	Normal	NA	NA	NA	NA	NA
2986	GSE13367	GSM337517	Normal	NA	NA	NA	NA	NA
2987	GSE13471	GSM339563	Normal	NA	NA	NA	NA	NA
2988	GSE13471	GSM339564	Normal	NA	NA	NA	NA	NA
2989	GSE13471	GSM339568	Normal	NA	NA	NA	NA	NA
2990	GSE13471	GSM339569	Normal	NA	NA	NA	NA	NA
2991	GSE14526	GSM363586	Normal	NA	NA	NA	NA	NA
2992	GSE14526	GSM363587	Normal	NA	NA	NA	NA	NA
2993	GSE16879	GSM364627	Normal	NA	NA	NA	NA	NA
2994	GSE16879	GSM364628	Normal	NA	NA	NA	NA	NA
2995	GSE16879	GSM364629	Normal	NA	NA	NA	NA	NA
2996	GSE16879	GSM364630	Normal	NA	NA	NA	NA	NA
2997	GSE16879	GSM364631	Normal	NA	NA	NA	NA	NA
2998	GSE16879	GSM364632	Normal	NA	NA	NA	NA	NA
2999	GSE18105	GSM452629	Normal	NA	NA	NA	NA	NA
3000	GSE18105	GSM452630	Normal	NA	NA	NA	NA	NA
3001	GSE18105	GSM452631	Normal	NA	NA	NA	NA	NA
3002	GSE18105	GSM452632	Normal	NA	NA	NA	NA	NA
3003	GSE18105	GSM452633	Normal	NA	NA	NA	NA	NA
3004	GSE18105	GSM452634	Normal	NA	NA	NA	NA	NA
3005	GSE18105	GSM452635	Normal	NA	NA	NA	NA	NA
3006	GSE18105	GSM452636	Normal	NA	NA	NA	NA	NA
3007	GSE18105	GSM452637	Normal	NA	NA	NA	NA	NA
3008	GSE18105	GSM452638	Normal	NA	NA	NA	NA	NA
3009	GSE18105	GSM452640	Normal	NA	NA	NA	NA	NA
3010	GSE18105	GSM452641	Normal	NA	NA	NA	NA	NA
3011	GSE18105	GSM452642	Normal	NA	NA	NA	NA	NA
3012	GSE18105	GSM452643	Normal	NA	NA	NA	NA	NA

3013	GSE18105	GSM452644	Normal	NA	NA	NA	NA	NA
3014	GSE18105	GSM452645	Normal	NA	NA	NA	NA	NA
3015	GSE18462	GSM459858	Normal	NA	NA	NA	NA	NA
3016	GSE18462	GSM459862	Normal	NA	NA	NA	NA	NA
3017	GSE19963	GSM498707	Normal	NA	NA	NA	NA	NA
3018	GSE19963	GSM498708	Normal	NA	NA	NA	NA	NA
3019	GSE19963	GSM498709	Normal	NA	NA	NA	NA	NA
3020	GSE19963	GSM498710	Normal	NA	NA	NA	NA	NA
3021	GSE20916	GSM523282	Normal	NA	NA	NA	NA	NA
3022	GSE20916	GSM523286	Normal	NA	NA	NA	NA	NA
3023	GSE20916	GSM523289	Normal	NA	NA	NA	NA	NA
3024	GSE20916	GSM523290	Normal	NA	NA	NA	NA	NA
3025	GSE20916	GSM523291	Normal	NA	NA	NA	NA	NA
3026	GSE20916	GSM523297	Normal	NA	NA	NA	NA	NA
3027	GSE20916	GSM523299	Normal	NA	NA	NA	NA	NA
3028	GSE20916	GSM523304	Normal	NA	NA	NA	NA	NA
3029	GSE20916	GSM523310	Normal	NA	NA	NA	NA	NA
3030	GSE20916	GSM523311	Normal	NA	NA	NA	NA	NA
3031	GSE20916	GSM523314	Normal	NA	NA	NA	NA	NA
3032	GSE20916	GSM523324	Normal	NA	NA	NA	NA	NA
3033	GSE20916	GSM523330	Normal	NA	NA	NA	NA	NA
3034	GSE20916	GSM523338	Normal	NA	NA	NA	NA	NA
3035	GSE20916	GSM523343	Normal	NA	NA	NA	NA	NA
3036	GSE20916	GSM523349	Normal	NA	NA	NA	NA	NA
3037	GSE20916	GSM523358	Normal	NA	NA	NA	NA	NA
3038	GSE20916	GSM523360	Normal	NA	NA	NA	NA	NA
3039	GSE20916	GSM523364	Normal	NA	NA	NA	NA	NA
3040	GSE20916	GSM523365	Normal	NA	NA	NA	NA	NA
3041	GSE20916	GSM523369	Normal	NA	NA	NA	NA	NA
3042	GSE20916	GSM523373	Normal	NA	NA	NA	NA	NA
3043	GSE20916	GSM523377	Normal	NA	NA	NA	NA	NA
3044	GSE20916	GSM523382	Normal	NA	NA	NA	NA	NA
3045	GSE22242	GSM552508	Normal	NA	NA	NA	NA	NA
3046	GSE22619	GSM1012177	Normal	NA	NA	NA	NA	NA
3047	GSE22619	GSM1012179	Normal	NA	NA	NA	NA	NA
3048	GSE22619	GSM560961	Normal	NA	NA	NA	NA	NA
3049	GSE22619	GSM560962	Normal	NA	NA	NA	NA	NA
3050	GSE22619	GSM560963	Normal	NA	NA	NA	NA	NA
3051	GSE22619	GSM560964	Normal	NA	NA	NA	NA	NA
3052	GSE22619	GSM560965	Normal	NA	NA	NA	NA	NA
3053	GSE22619	GSM560966	Normal	NA	NA	NA	NA	NA
3054	GSE22619	GSM560967	Normal	NA	NA	NA	NA	NA
3055	GSE22619	GSM560968	Normal	NA	NA	NA	NA	NA
3056	GSE23194	GSM570810	Normal	NA	NA	NA	NA	NA
3057	GSE23194	GSM570811	Normal	NA	NA	NA	NA	NA

3058	GSE23194	GSM570812	Normal	NA	NA	NA	NA	NA
3059	GSE23194	GSM570813	Normal	NA	NA	NA	NA	NA
3060	GSE23194	GSM570814	Normal	NA	NA	NA	NA	NA
3061	GSE23194	GSM570815	Normal	NA	NA	NA	NA	NA
3062	GSE23194	GSM570816	Normal	NA	NA	NA	NA	NA
3063	GSE23194	GSM570817	Normal	NA	NA	NA	NA	NA
3064	GSE23194	GSM570818	Normal	NA	NA	NA	NA	NA
3065	GSE23194	GSM570819	Normal	NA	NA	NA	NA	NA
3066	GSE23194	GSM570820	Normal	NA	NA	NA	NA	NA
3067	GSE23194	GSM570821	Normal	NA	NA	NA	NA	NA
3068	GSE23194	GSM570822	Normal	NA	NA	NA	NA	NA
3069	GSE23194	GSM570823	Normal	NA	NA	NA	NA	NA
3070	GSE23194	GSM570824	Normal	NA	NA	NA	NA	NA
3071	GSE23194	GSM570825	Normal	NA	NA	NA	NA	NA
3072	GSE23194	GSM570826	Normal	NA	NA	NA	NA	NA
3073	GSE23878	GSM588863	Normal	NA	NA	NA	NA	NA
3074	GSE23878	GSM588864	Normal	NA	NA	NA	NA	NA
3075	GSE23878	GSM588865	Normal	NA	NA	NA	NA	NA
3076	GSE23878	GSM588866	Normal	NA	NA	NA	NA	NA
3077	GSE23878	GSM588867	Normal	NA	NA	NA	NA	NA
3078	GSE23878	GSM588868	Normal	NA	NA	NA	NA	NA
3079	GSE23878	GSM588869	Normal	NA	NA	NA	NA	NA
3080	GSE23878	GSM588870	Normal	NA	NA	NA	NA	NA
3081	GSE23878	GSM588871	Normal	NA	NA	NA	NA	NA
3082	GSE23878	GSM588873	Normal	NA	NA	NA	NA	NA
3083	GSE23878	GSM588874	Normal	NA	NA	NA	NA	NA
3084	GSE23878	GSM588875	Normal	NA	NA	NA	NA	NA
3085	GSE23878	GSM588876	Normal	NA	NA	NA	NA	NA
3086	GSE23878	GSM588877	Normal	NA	NA	NA	NA	NA
3087	GSE23878	GSM588878	Normal	NA	NA	NA	NA	NA
3088	GSE23878	GSM588879	Normal	NA	NA	NA	NA	NA
3089	GSE23878	GSM588880	Normal	NA	NA	NA	NA	NA
3090	GSE23878	GSM588881	Normal	NA	NA	NA	NA	NA
3091	GSE23878	GSM588882	Normal	NA	NA	NA	NA	NA
3092	GSE23878	GSM588883	Normal	NA	NA	NA	NA	NA
3093	GSE23878	GSM588884	Normal	NA	NA	NA	NA	NA
3094	GSE23878	GSM588885	Normal	NA	NA	NA	NA	NA
3095	GSE23878	GSM588886	Normal	NA	NA	NA	NA	NA
3096	GSE31255	GSM774695	Normal	NA	NA	NA	NA	NA
3097	GSE31255	GSM774696	Normal	NA	NA	NA	NA	NA
3098	GSE31255	GSM774701	Normal	NA	NA	NA	NA	NA
3099	GSE31255	GSM774703	Normal	NA	NA	NA	NA	NA
3100	GSE31255	GSM774705	Normal	NA	NA	NA	NA	NA
3101	GSE33113	GSM1100477	Normal	NA	NA	NA	NA	NA
3102	GSE33113	GSM1100478	Normal	NA	NA	NA	NA	NA

3103	GSE33113	GSM1100479	Normal	NA	NA	NA	NA	NA
3104	GSE33113	GSM1100480	Normal	NA	NA	NA	NA	NA
3105	GSE33113	GSM1100481	Normal	NA	NA	NA	NA	NA
3106	GSE33113	GSM1100482	Normal	NA	NA	NA	NA	NA
3107	GSE33842	GSM837752	Normal	NA	NA	NA	NA	NA
3108	GSE36807	GSM901319	Normal	NA	NA	NA	NA	NA
3109	GSE36807	GSM901320	Normal	NA	NA	NA	NA	NA
3110	GSE36807	GSM901321	Normal	NA	NA	NA	NA	NA
3111	GSE36807	GSM901322	Normal	NA	NA	NA	NA	NA
3112	GSE36807	GSM901323	Normal	NA	NA	NA	NA	NA
3113	GSE36807	GSM901324	Normal	NA	NA	NA	NA	NA
3114	GSE36807	GSM901325	Normal	NA	NA	NA	NA	NA
3115	GSE37267	GSM915207	Normal	NA	NA	NA	NA	NA
3116	GSE37267	GSM915208	Normal	NA	NA	NA	NA	NA
3117	GSE37267	GSM915209	Normal	NA	NA	NA	NA	NA
3118	GSE37267	GSM915210	Normal	NA	NA	NA	NA	NA
3119	GSE37267	GSM915211	Normal	NA	NA	NA	NA	NA
3120	GSE37267	GSM915212	Normal	NA	NA	NA	NA	NA
3121	GSE37364	GSM916743	Normal	NA	NA	NA	NA	NA
3122	GSE37364	GSM916744	Normal	NA	NA	NA	NA	NA
3123	GSE37364	GSM916745	Normal	NA	NA	NA	NA	NA
3124	GSE37364	GSM916746	Normal	NA	NA	NA	NA	NA
3125	GSE37364	GSM916747	Normal	NA	NA	NA	NA	NA
3126	GSE37364	GSM916748	Normal	NA	NA	NA	NA	NA
3127	GSE37364	GSM916749	Normal	NA	NA	NA	NA	NA
3128	GSE37364	GSM916750	Normal	NA	NA	NA	NA	NA
3129	GSE37364	GSM916751	Normal	NA	NA	NA	NA	NA
3130	GSE37364	GSM916752	Normal	NA	NA	NA	NA	NA
3131	GSE37364	GSM916753	Normal	NA	NA	NA	NA	NA
3132	GSE37364	GSM916754	Normal	NA	NA	NA	NA	NA
3133	GSE37364	GSM916755	Normal	NA	NA	NA	NA	NA
3134	GSE37364	GSM916756	Normal	NA	NA	NA	NA	NA
3135	GSE37364	GSM916757	Normal	NA	NA	NA	NA	NA
3136	GSE37364	GSM916758	Normal	NA	NA	NA	NA	NA
3137	GSE37364	GSM916759	Normal	NA	NA	NA	NA	NA
3138	GSE37364	GSM916760	Normal	NA	NA	NA	NA	NA
3139	GSE37364	GSM916761	Normal	NA	NA	NA	NA	NA
3140	GSE37364	GSM916762	Normal	NA	NA	NA	NA	NA
3141	GSE37364	GSM916763	Normal	NA	NA	NA	NA	NA
3142	GSE37364	GSM916764	Normal	NA	NA	NA	NA	NA
3143	GSE37364	GSM916765	Normal	NA	NA	NA	NA	NA
3144	GSE37364	GSM916766	Normal	NA	NA	NA	NA	NA
3145	GSE37364	GSM916767	Normal	NA	NA	NA	NA	NA
3146	GSE37364	GSM916768	Normal	NA	NA	NA	NA	NA
3147	GSE37364	GSM916769	Normal	NA	NA	NA	NA	NA

3148	GSE37364	GSM916770	Normal	NA	NA	NA	NA	NA
3149	GSE37364	GSM916771	Normal	NA	NA	NA	NA	NA
3150	GSE37364	GSM916772	Normal	NA	NA	NA	NA	NA
3151	GSE37364	GSM916773	Normal	NA	NA	NA	NA	NA
3152	GSE37364	GSM916774	Normal	NA	NA	NA	NA	NA
3153	GSE37364	GSM916775	Normal	NA	NA	NA	NA	NA
3154	GSE37364	GSM916776	Normal	NA	NA	NA	NA	NA
3155	GSE37364	GSM916777	Normal	NA	NA	NA	NA	NA
3156	GSE37364	GSM916778	Normal	NA	NA	NA	NA	NA
3157	GSE37364	GSM916779	Normal	NA	NA	NA	NA	NA
3158	GSE37364	GSM916780	Normal	NA	NA	NA	NA	NA
3159	GSE38713	GSM948550	Normal	NA	NA	NA	NA	NA
3160	GSE38713	GSM948551	Normal	NA	NA	NA	NA	NA
3161	GSE38713	GSM948552	Normal	NA	NA	NA	NA	NA
3162	GSE38713	GSM948553	Normal	NA	NA	NA	NA	NA
3163	GSE38713	GSM948554	Normal	NA	NA	NA	NA	NA
3164	GSE38713	GSM948555	Normal	NA	NA	NA	NA	NA
3165	GSE38713	GSM948556	Normal	NA	NA	NA	NA	NA
3166	GSE38713	GSM948557	Normal	NA	NA	NA	NA	NA
3167	GSE38713	GSM948558	Normal	NA	NA	NA	NA	NA
3168	GSE38713	GSM948559	Normal	NA	NA	NA	NA	NA
3169	GSE38713	GSM948560	Normal	NA	NA	NA	NA	NA
3170	GSE38713	GSM948561	Normal	NA	NA	NA	NA	NA
3171	GSE38713	GSM948562	Normal	NA	NA	NA	NA	NA
3172	GSE39582	GSM1681353	Normal	NA	NA	NA	NA	NA
3173	GSE39582	GSM1681354	Normal	NA	NA	NA	NA	NA
3174	GSE39582	GSM1681355	Normal	NA	NA	NA	NA	NA
3175	GSE39582	GSM1681356	Normal	NA	NA	NA	NA	NA
3176	GSE39582	GSM1681357	Normal	NA	NA	NA	NA	NA
3177	GSE39582	GSM1681358	Normal	NA	NA	NA	NA	NA
3178	GSE39582	GSM1681359	Normal	NA	NA	NA	NA	NA
3179	GSE39582	GSM1681360	Normal	NA	NA	NA	NA	NA
3180	GSE39582	GSM1681361	Normal	NA	NA	NA	NA	NA
3181	GSE39582	GSM1681362	Normal	NA	NA	NA	NA	NA
3182	GSE39582	GSM1681363	Normal	NA	NA	NA	NA	NA
3183	GSE39582	GSM1681364	Normal	NA	NA	NA	NA	NA
3184	GSE39582	GSM1681365	Normal	NA	NA	NA	NA	NA
3185	GSE39582	GSM1681366	Normal	NA	NA	NA	NA	NA
3186	GSE39582	GSM1681367	Normal	NA	NA	NA	NA	NA
3187	GSE39582	GSM1681368	Normal	NA	NA	NA	NA	NA
3188	GSE39582	GSM1681369	Normal	NA	NA	NA	NA	NA
3189	GSE39582	GSM1681370	Normal	NA	NA	NA	NA	NA
3190	GSE39582	GSM1681371	Normal	NA	NA	NA	NA	NA
3191	GSE40220	GSM988527	Normal	NA	NA	NA	NA	NA
3192	GSE40220	GSM988531	Normal	NA	NA	NA	NA	NA

3193	GSE40220	GSM988535	Normal	NA	NA	NA	NA	NA
3194	GSE4107	GSM93938	Normal	NA	NA	NA	NA	NA
3195	GSE4107	GSM93939	Normal	NA	NA	NA	NA	NA
3196	GSE4107	GSM93941	Normal	NA	NA	NA	NA	NA
3197	GSE4107	GSM93943	Normal	NA	NA	NA	NA	NA
3198	GSE4107	GSM93944	Normal	NA	NA	NA	NA	NA
3199	GSE4107	GSM93946	Normal	NA	NA	NA	NA	NA
3200	GSE4107	GSM93948	Normal	NA	NA	NA	NA	NA
3201	GSE4107	GSM93950	Normal	NA	NA	NA	NA	NA
3202	GSE4107	GSM93952	Normal	NA	NA	NA	NA	NA
3203	GSE4107	GSM93954	Normal	NA	NA	NA	NA	NA
3204	GSE41328	GSM1014798	Normal	NA	NA	NA	NA	NA
3205	GSE41328	GSM1014799	Normal	NA	NA	NA	NA	NA
3206	GSE41328	GSM1014800	Normal	NA	NA	NA	NA	NA
3207	GSE41328	GSM1014801	Normal	NA	NA	NA	NA	NA
3208	GSE41328	GSM1014802	Normal	NA	NA	NA	NA	NA
3209	GSE41328	GSM1014808	Normal	NA	NA	NA	NA	NA
3210	GSE41328	GSM1014809	Normal	NA	NA	NA	NA	NA
3211	GSE41328	GSM1014810	Normal	NA	NA	NA	NA	NA
3212	GSE41328	GSM1014811	Normal	NA	NA	NA	NA	NA
3213	GSE41328	GSM1014812	Normal	NA	NA	NA	NA	NA
3214	GSE4183	GSM95473	Normal	NA	NA	NA	NA	NA
3215	GSE4183	GSM95474	Normal	NA	NA	NA	NA	NA
3216	GSE4183	GSM95475	Normal	NA	NA	NA	NA	NA
3217	GSE4183	GSM95476	Normal	NA	NA	NA	NA	NA
3218	GSE4183	GSM95477	Normal	NA	NA	NA	NA	NA
3219	GSE4183	GSM95478	Normal	NA	NA	NA	NA	NA
3220	GSE4183	GSM95479	Normal	NA	NA	NA	NA	NA
3221	GSE4183	GSM95480	Normal	NA	NA	NA	NA	NA
3222	GSE47908	GSM1162227	Normal	NA	NA	NA	NA	NA
3223	GSE47908	GSM1162228	Normal	NA	NA	NA	NA	NA
3224	GSE47908	GSM1162229	Normal	NA	NA	NA	NA	NA
3225	GSE47908	GSM1162230	Normal	NA	NA	NA	NA	NA
3226	GSE47908	GSM1162231	Normal	NA	NA	NA	NA	NA
3227	GSE47908	GSM1162232	Normal	NA	NA	NA	NA	NA
3228	GSE47908	GSM1162233	Normal	NA	NA	NA	NA	NA
3229	GSE47908	GSM1162234	Normal	NA	NA	NA	NA	NA
3230	GSE47908	GSM1162235	Normal	NA	NA	NA	NA	NA
3231	GSE47908	GSM1162236	Normal	NA	NA	NA	NA	NA
3232	GSE47908	GSM1162237	Normal	NA	NA	NA	NA	NA
3233	GSE47908	GSM1162238	Normal	NA	NA	NA	NA	NA
3234	GSE47908	GSM1162239	Normal	NA	NA	NA	NA	NA
3235	GSE47908	GSM1162240	Normal	NA	NA	NA	NA	NA
3236	GSE47908	GSM1162241	Normal	NA	NA	NA	NA	NA
3237	GSE52746	GSM1275207	Normal	NA	NA	NA	NA	NA

3238	GSE52746	GSM1275208	Normal	NA	NA	NA	NA	NA
3239	GSE52746	GSM1275209	Normal	NA	NA	NA	NA	NA
3240	GSE52746	GSM1275210	Normal	NA	NA	NA	NA	NA
3241	GSE52746	GSM1275211	Normal	NA	NA	NA	NA	NA
3242	GSE52746	GSM1275212	Normal	NA	NA	NA	NA	NA
3243	GSE52746	GSM1275213	Normal	NA	NA	NA	NA	NA
3244	GSE52746	GSM1275214	Normal	NA	NA	NA	NA	NA
3245	GSE52746	GSM1275215	Normal	NA	NA	NA	NA	NA
3246	GSE52746	GSM1275216	Normal	NA	NA	NA	NA	NA
3247	GSE52746	GSM1275217	Normal	NA	NA	NA	NA	NA
3248	GSE52746	GSM1275218	Normal	NA	NA	NA	NA	NA
3249	GSE52746	GSM1275219	Normal	NA	NA	NA	NA	NA
3250	GSE52746	GSM1275220	Normal	NA	NA	NA	NA	NA
3251	GSE52746	GSM1275221	Normal	NA	NA	NA	NA	NA
3252	GSE52746	GSM1275222	Normal	NA	NA	NA	NA	NA
3253	GSE52746	GSM1275223	Normal	NA	NA	NA	NA	NA
3254	GSE8671	GSM215051	Normal	NA	NA	NA	NA	NA
3255	GSE8671	GSM215052	Normal	NA	NA	NA	NA	NA
3256	GSE8671	GSM215053	Normal	NA	NA	NA	NA	NA
3257	GSE8671	GSM215054	Normal	NA	NA	NA	NA	NA
3258	GSE8671	GSM215055	Normal	NA	NA	NA	NA	NA
3259	GSE8671	GSM215056	Normal	NA	NA	NA	NA	NA
3260	GSE8671	GSM215057	Normal	NA	NA	NA	NA	NA
3261	GSE8671	GSM215058	Normal	NA	NA	NA	NA	NA
3262	GSE8671	GSM215059	Normal	NA	NA	NA	NA	NA
3263	GSE8671	GSM215060	Normal	NA	NA	NA	NA	NA
3264	GSE8671	GSM215061	Normal	NA	NA	NA	NA	NA
3265	GSE8671	GSM215062	Normal	NA	NA	NA	NA	NA
3266	GSE8671	GSM215063	Normal	NA	NA	NA	NA	NA
3267	GSE8671	GSM215064	Normal	NA	NA	NA	NA	NA
3268	GSE8671	GSM215065	Normal	NA	NA	NA	NA	NA
3269	GSE8671	GSM215066	Normal	NA	NA	NA	NA	NA
3270	GSE8671	GSM215067	Normal	NA	NA	NA	NA	NA
3271	GSE8671	GSM215068	Normal	NA	NA	NA	NA	NA
3272	GSE8671	GSM215069	Normal	NA	NA	NA	NA	NA
3273	GSE8671	GSM215070	Normal	NA	NA	NA	NA	NA
3274	GSE8671	GSM215071	Normal	NA	NA	NA	NA	NA
3275	GSE8671	GSM215072	Normal	NA	NA	NA	NA	NA
3276	GSE8671	GSM215073	Normal	NA	NA	NA	NA	NA
3277	GSE8671	GSM215074	Normal	NA	NA	NA	NA	NA
3278	GSE8671	GSM215075	Normal	NA	NA	NA	NA	NA
3279	GSE8671	GSM215076	Normal	NA	NA	NA	NA	NA
3280	GSE8671	GSM215077	Normal	NA	NA	NA	NA	NA
3281	GSE8671	GSM215078	Normal	NA	NA	NA	NA	NA
3282	GSE8671	GSM215079	Normal	NA	NA	NA	NA	NA

3283	GSE8671	GSM215080	Normal	NA	NA	NA	NA	NA
3284	GSE8671	GSM215081	Normal	NA	NA	NA	NA	NA
3285	GSE8671	GSM215082	Normal	NA	NA	NA	NA	NA
3286	GSE9254	GSM234909	Normal	NA	NA	NA	NA	NA
3287	GSE9254	GSM234913	Normal	NA	NA	NA	NA	NA
3288	GSE9254	GSM234914	Normal	NA	NA	NA	NA	NA
3289	GSE9254	GSM234915	Normal	NA	NA	NA	NA	NA
3290	GSE9254	GSM234916	Normal	NA	NA	NA	NA	NA
3291	GSE9254	GSM234917	Normal	NA	NA	NA	NA	NA
3292	GSE9254	GSM234920	Normal	NA	NA	NA	NA	NA
3293	GSE9254	GSM234921	Normal	NA	NA	NA	NA	NA
3294	GSE9254	GSM234922	Normal	NA	NA	NA	NA	NA
3295	GSE9254	GSM234925	Normal	NA	NA	NA	NA	NA
3296	GSE9254	GSM234926	Normal	NA	NA	NA	NA	NA
3297	GSE9254	GSM234927	Normal	NA	NA	NA	NA	NA
3298	GSE9348	GSM237984	Normal	NA	NA	NA	NA	NA
3299	GSE9348	GSM237985	Normal	NA	NA	NA	NA	NA
3300	GSE9348	GSM237986	Normal	NA	NA	NA	NA	NA
3301	GSE9348	GSM237987	Normal	NA	NA	NA	NA	NA
3302	GSE9348	GSM237988	Normal	NA	NA	NA	NA	NA
3303	GSE9348	GSM237989	Normal	NA	NA	NA	NA	NA
3304	GSE9348	GSM237990	Normal	NA	NA	NA	NA	NA
3305	GSE9348	GSM237991	Normal	NA	NA	NA	NA	NA
3306	GSE9348	GSM237992	Normal	NA	NA	NA	NA	NA
3307	GSE9348	GSM237993	Normal	NA	NA	NA	NA	NA
3308	GSE9348	GSM237994	Normal	NA	NA	NA	NA	NA
3309	GSE9348	GSM237995	Normal	NA	NA	NA	NA	NA
3310	GSE9452	GSM239723	Normal	NA	NA	NA	NA	NA
3311	GSE9452	GSM239725	Normal	NA	NA	NA	NA	NA
3312	GSE9452	GSM239726	Normal	NA	NA	NA	NA	NA
3313	GSE9452	GSM239727	Normal	NA	NA	NA	NA	NA
3314	GSE9452	GSM239729	Normal	NA	NA	NA	NA	NA
3315	GSE9452	GSM239730	Normal	NA	NA	NA	NA	NA
3316	GSE9452	GSM239731	Normal	NA	NA	NA	NA	NA
3317	GSE9452	GSM239732	Normal	NA	NA	NA	NA	NA
3318	GSE9452	GSM240022	Normal	NA	NA	NA	NA	NA
3319	GSE9452	GSM240023	Normal	NA	NA	NA	NA	NA
3320	GSE9452	GSM240024	Normal	NA	NA	NA	NA	NA
3321	GSE9452	GSM240025	Normal	NA	NA	NA	NA	NA
3322	GSE9452	GSM240026	Normal	NA	NA	NA	NA	NA
3323	GSE9452	GSM240027	Normal	NA	NA	NA	NA	NA
3324	GSE9452	GSM240028	Normal	NA	NA	NA	NA	NA
3325	GSE9452	GSM240029	Normal	NA	NA	NA	NA	NA
3326	GSE9452	GSM240030	Normal	NA	NA	NA	NA	NA
3327	GSE9452	GSM240031	Normal	NA	NA	NA	NA	NA

3328	GSE10714	GSM270781	Polyps	NA	NA	NA	NA	NA
3329	GSE10714	GSM270782	Polyps	NA	NA	NA	NA	NA
3330	GSE10714	GSM270783	Polyps	NA	NA	NA	NA	NA
3331	GSE10714	GSM270784	Polyps	NA	NA	NA	NA	NA
3332	GSE10714	GSM270785	Polyps	NA	NA	NA	NA	NA
3333	GSE10714	GSM270797	Polyps	NA	NA	NA	NA	NA
3334	GSE10714	GSM270798	Polyps	NA	NA	NA	NA	NA
3335	GSE10714	GSM270799	Polyps	NA	NA	NA	NA	NA
3336	GSE10714	GSM270800	Polyps	NA	NA	NA	NA	NA
3337	GSE10714	GSM270801	Polyps	NA	NA	NA	NA	NA
3338	GSE10714	GSM270802	Polyps	NA	NA	NA	NA	NA
3339	GSE10714	GSM270803	Polyps	NA	NA	NA	NA	NA
3340	GSE10714	GSM270804	Polyps	NA	NA	NA	NA	NA
3341	GSE10714	GSM270805	Polyps	NA	NA	NA	NA	NA
3342	GSE10714	GSM270806	Polyps	NA	NA	NA	NA	NA
3343	GSE10714	GSM270807	Polyps	NA	NA	NA	NA	NA
3344	GSE19963	GSM498702	Polyps	NA	NA	NA	NA	NA
3345	GSE19963	GSM498703	Polyps	NA	NA	NA	NA	NA
3346	GSE19963	GSM498704	Polyps	NA	NA	NA	NA	NA
3347	GSE19963	GSM498705	Polyps	NA	NA	NA	NA	NA
3348	GSE19963	GSM498706	Polyps	NA	NA	NA	NA	NA
3349	GSE22242	GSM552509	Polyps	NA	NA	NA	NA	NA
3350	GSE37364	GSM916687	Polyps	NA	NA	NA	NA	NA
3351	GSE37364	GSM916688	Polyps	NA	NA	NA	NA	NA
3352	GSE37364	GSM916689	Polyps	NA	NA	NA	NA	NA
3353	GSE37364	GSM916690	Polyps	NA	NA	NA	NA	NA
3354	GSE37364	GSM916691	Polyps	NA	NA	NA	NA	NA
3355	GSE37364	GSM916692	Polyps	NA	NA	NA	NA	NA
3356	GSE37364	GSM916693	Polyps	NA	NA	NA	NA	NA
3357	GSE37364	GSM916694	Polyps	NA	NA	NA	NA	NA
3358	GSE37364	GSM916695	Polyps	NA	NA	NA	NA	NA
3359	GSE37364	GSM916696	Polyps	NA	NA	NA	NA	NA
3360	GSE37364	GSM916697	Polyps	NA	NA	NA	NA	NA
3361	GSE37364	GSM916698	Polyps	NA	NA	NA	NA	NA
3362	GSE37364	GSM916699	Polyps	NA	NA	NA	NA	NA
3363	GSE37364	GSM916700	Polyps	NA	NA	NA	NA	NA
3364	GSE37364	GSM916701	Polyps	NA	NA	NA	NA	NA
3365	GSE37364	GSM916702	Polyps	NA	NA	NA	NA	NA
3366	GSE37364	GSM916703	Polyps	NA	NA	NA	NA	NA
3367	GSE37364	GSM916704	Polyps	NA	NA	NA	NA	NA
3368	GSE37364	GSM916705	Polyps	NA	NA	NA	NA	NA
3369	GSE37364	GSM916706	Polyps	NA	NA	NA	NA	NA
3370	GSE37364	GSM916707	Polyps	NA	NA	NA	NA	NA
3371	GSE37364	GSM916708	Polyps	NA	NA	NA	NA	NA
3372	GSE37364	GSM916709	Polyps	NA	NA	NA	NA	NA

3373	GSE37364	GSM916710	Polyps	NA	NA	NA	NA	NA
3374	GSE37364	GSM916711	Polyps	NA	NA	NA	NA	NA
3375	GSE37364	GSM916712	Polyps	NA	NA	NA	NA	NA
3376	GSE37364	GSM916713	Polyps	NA	NA	NA	NA	NA
3377	GSE37364	GSM916714	Polyps	NA	NA	NA	NA	NA
3378	GSE37364	GSM916715	Polyps	NA	NA	NA	NA	NA
3379	GSE4183	GSM95481	Polyps	NA	NA	NA	NA	NA
3380	GSE4183	GSM95482	Polyps	NA	NA	NA	NA	NA
3381	GSE4183	GSM95483	Polyps	NA	NA	NA	NA	NA
3382	GSE4183	GSM95484	Polyps	NA	NA	NA	NA	NA
3383	GSE4183	GSM95485	Polyps	NA	NA	NA	NA	NA
3384	GSE4183	GSM95486	Polyps	NA	NA	NA	NA	NA
3385	GSE4183	GSM95487	Polyps	NA	NA	NA	NA	NA
3386	GSE4183	GSM95488	Polyps	NA	NA	NA	NA	NA
3387	GSE4183	GSM95489	Polyps	NA	NA	NA	NA	NA
3388	GSE4183	GSM95490	Polyps	NA	NA	NA	NA	NA
3389	GSE4183	GSM95491	Polyps	NA	NA	NA	NA	NA
3390	GSE4183	GSM95492	Polyps	NA	NA	NA	NA	NA
3391	GSE4183	GSM95493	Polyps	NA	NA	NA	NA	NA
3392	GSE4183	GSM95494	Polyps	NA	NA	NA	NA	NA
3393	GSE4183	GSM95495	Polyps	NA	NA	NA	NA	NA
3394	GSE8671	GSM215083	Polyps	NA	NA	NA	NA	NA
3395	GSE8671	GSM215084	Polyps	NA	NA	NA	NA	NA
3396	GSE8671	GSM215085	Polyps	NA	NA	NA	NA	NA
3397	GSE8671	GSM215086	Polyps	NA	NA	NA	NA	NA
3398	GSE8671	GSM215087	Polyps	NA	NA	NA	NA	NA
3399	GSE8671	GSM215088	Polyps	NA	NA	NA	NA	NA
3400	GSE8671	GSM215089	Polyps	NA	NA	NA	NA	NA
3401	GSE8671	GSM215090	Polyps	NA	NA	NA	NA	NA
3402	GSE8671	GSM215091	Polyps	NA	NA	NA	NA	NA
3403	GSE8671	GSM215092	Polyps	NA	NA	NA	NA	NA
3404	GSE8671	GSM215093	Polyps	NA	NA	NA	NA	NA
3405	GSE8671	GSM215094	Polyps	NA	NA	NA	NA	NA
3406	GSE8671	GSM215095	Polyps	NA	NA	NA	NA	NA
3407	GSE8671	GSM215096	Polyps	NA	NA	NA	NA	NA
3408	GSE8671	GSM215097	Polyps	NA	NA	NA	NA	NA
3409	GSE8671	GSM215098	Polyps	NA	NA	NA	NA	NA
3410	GSE8671	GSM215099	Polyps	NA	NA	NA	NA	NA
3411	GSE8671	GSM215100	Polyps	NA	NA	NA	NA	NA
3412	GSE8671	GSM215101	Polyps	NA	NA	NA	NA	NA
3413	GSE8671	GSM215102	Polyps	NA	NA	NA	NA	NA
3414	GSE8671	GSM215103	Polyps	NA	NA	NA	NA	NA
3415	GSE8671	GSM215104	Polyps	NA	NA	NA	NA	NA
3416	GSE8671	GSM215105	Polyps	NA	NA	NA	NA	NA
3417	GSE8671	GSM215106	Polyps	NA	NA	NA	NA	NA

3418	GSE8671	GSM215107	Polyps	NA	NA	NA	NA	NA
3419	GSE8671	GSM215108	Polyps	NA	NA	NA	NA	NA
3420	GSE8671	GSM215109	Polyps	NA	NA	NA	NA	NA
3421	GSE8671	GSM215110	Polyps	NA	NA	NA	NA	NA
3422	GSE8671	GSM215111	Polyps	NA	NA	NA	NA	NA
3423	GSE8671	GSM215112	Polyps	NA	NA	NA	NA	NA
3424	GSE8671	GSM215113	Polyps	NA	NA	NA	NA	NA
3425	GSE8671	GSM215114	Polyps	NA	NA	NA	NA	NA

Supplementary Table 2. Identification of five gene candidates with Boolean logic searching

Probes	Gene Name	Sample number				FDR	Sample ratio
		$VIM^{low}/PPM1H^{low}$	$VIM^{low}/PPM1H^{high}$	$VIM^{high}/PPM1H^{low}$	$VIM^{high}/PPM1H^{high}$		
10023_s_at	PCGF1	0	224	36	1593	0.000	0.75
236471_at	NFE2L3	0	203	195	1195	0.000	0.64
218412_s_at	GTF2IRD1	0	176	87	1071	0.000	0.54
212686_at	PPM1H	1	135	495	559	0.002	0.48
224903_at	CIRH1A	0	156	86	813	0.000	0.43

Supplementary Table 3. Cox model analysis for PPM1H/VIM scoring system in NCBI-GEO data set

Characteristics	Univariate analysis		Multivariate analysis	
	HR (95% CI)	P-value	HR (95% CI)	P-value
PPM1H-VIM system	1.520 (1.177-1.963)	0.001	1.377 (1.060-1.789)	0.016
Tumor stage, per increase in stage	2.315 (1.819-2.954)	<0.001	2.226 (1.745-2.840)	<0.001
Age (<60 y *vs.*≥60 y)	0.797 (0.595-1.067)	0.127	0.867 (0.647-1.161)	0.338
Sex (male *vs.* female)	1.163 (0.887-1.525)	0.275	1.182 (0.901-1.550)	0.229

Supplementary Table 4. Sequences of siRNA duplexs and qPCR primers for this study.

	siRNA duplexes: 5'-3'	Concentration used
PPM1H-siRNA1	CCAGAGGUAAGAAUCUACGAUCUUU (sense)	20nM
	AAAGAUCGUAGAUUCUUACCUCUGG (antisense)	
PPM1H-siRNA2	CCUGCCACUAUUGGUCGCUGUUUGA (sense)	20nM
	UCAAACAGCGACCAAUAGUGGCAGG (antisense)	
PPM1H-siRNA3	GCGCUUGAAAGUGCAUUCAAGGAAA (sense)	20nM
	UUUCCUUGAAUGCACUUUCAAGCGC (antisense)	
Control-siRNA	UUCUCCGAACGUGUCACGUTT (sense)	20nM
	ACGUGACACGUUCGGAGAATT (antisense)	

	Primers: 5'-3'	PCR program
PPM1H	GCATCCCTGGAAAAGTCAAA (forward)	Denatured 5 min at 95°C, and followed by 40 cycles of 95°C for 10s, 60°C for 15s and 72°C for 25s
	GAGGCTATTCGAGCAAGCAG (reverse)	
VIM	CGAAACTTCTCAGCATCACG (forward)	
	GCAGAAAGGCACTTGAAAGC (reverse)	
CDH1	CAAAGCACCTGTGAGCTTGC (forward)	
	TGTAGCTCTCGGCGTCAAAG (reverse)	
CDH2	AGGCTTCTGGTGAAATCGCA (forward)	
	TGGAAAGCTTCTCACGGCAT (reverse)	
GAPDH	GGAGCGAGATCCCTCCAAAAT (forward)	
	GGCTGTTGTCATACTTCTCATGG (reverse)	

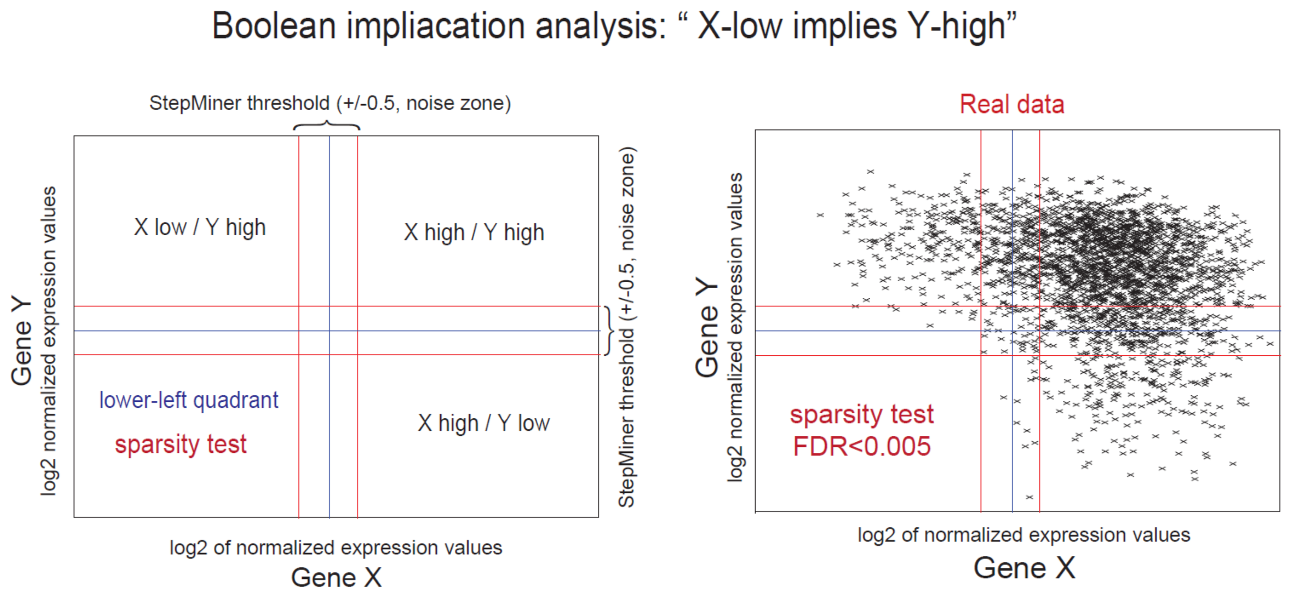

Supplementary Figure 1. **Mining pairs of genes whose expression is satisfied Boolean implications.** We searched the collected databases based on an implication of “X-low implies Y-high”. Thresholds of gene expression levels were identified using the the StepMiner algorithm. The Boolean implication will be considered between pairs of genes when the false-discovery rate (FDR) of a sparsity test in the lower left quadrant was < 0.005. The detailed method is provided in the supplementary Methods.

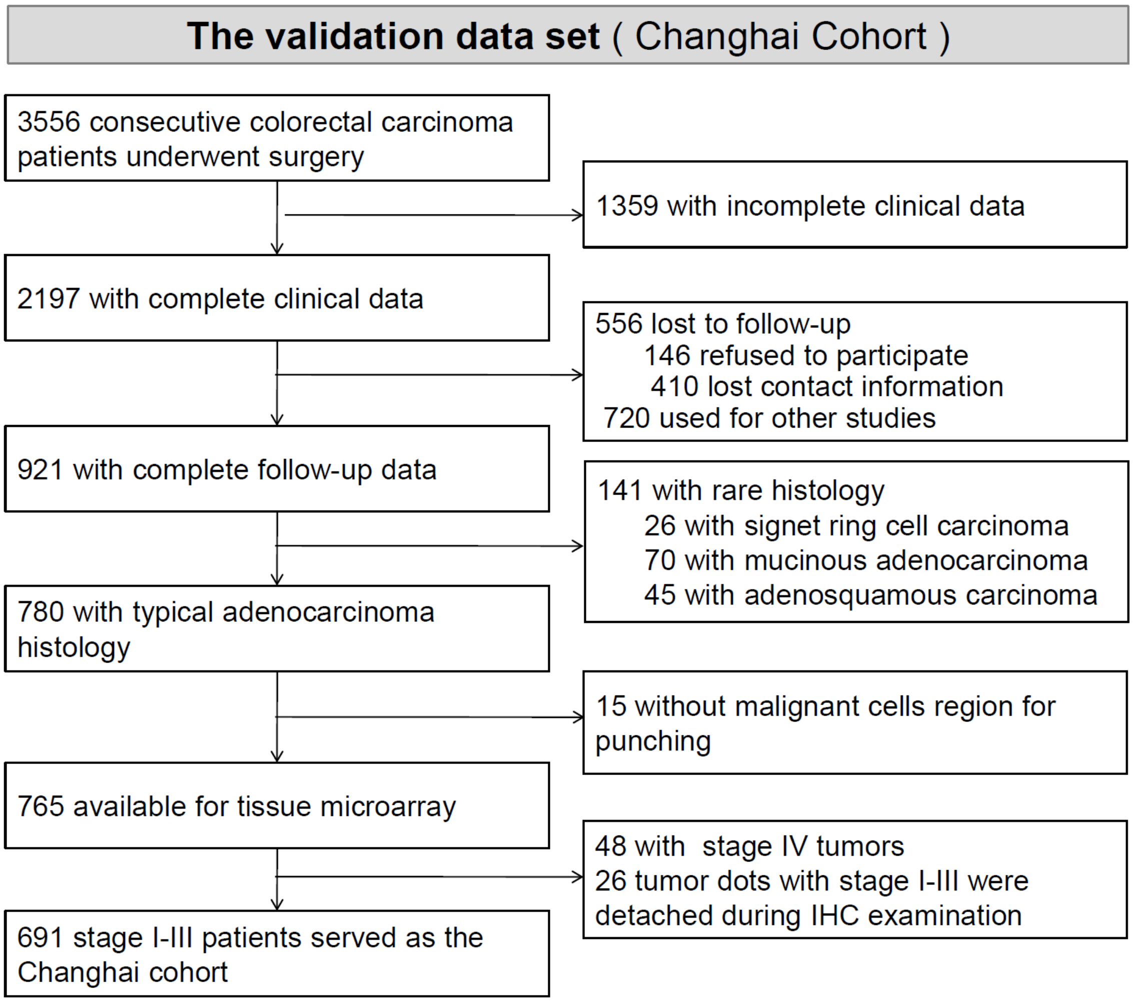

Supplementary Figure 2. **Flow diagram and inclusion criteria of patients in Changhai cohort.**

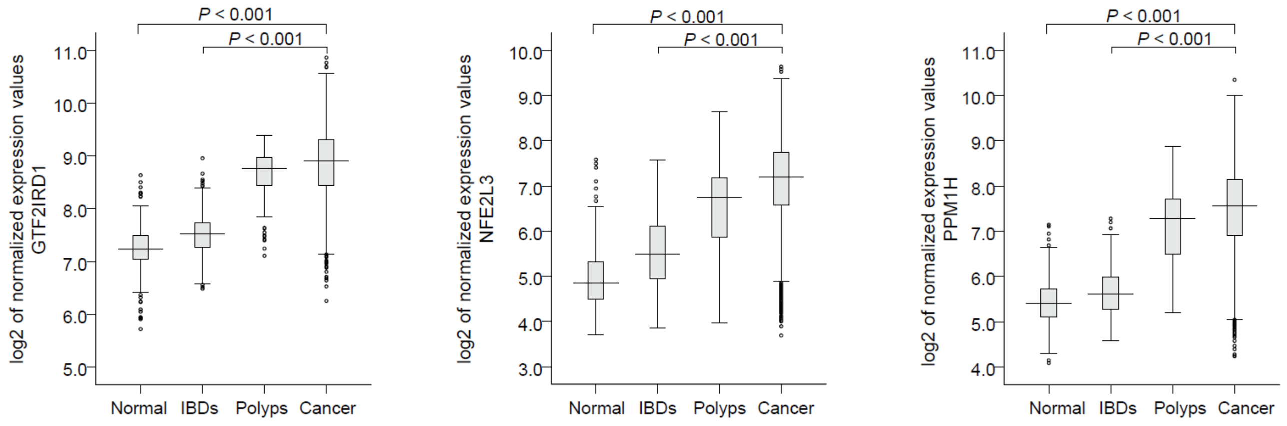

Supplementary Figure 3. **The mRNA expression levels of GTF2IRD1, NFE2L3, and PPM1H among different colorectal epithelial tissues across the collected microarray databases**.

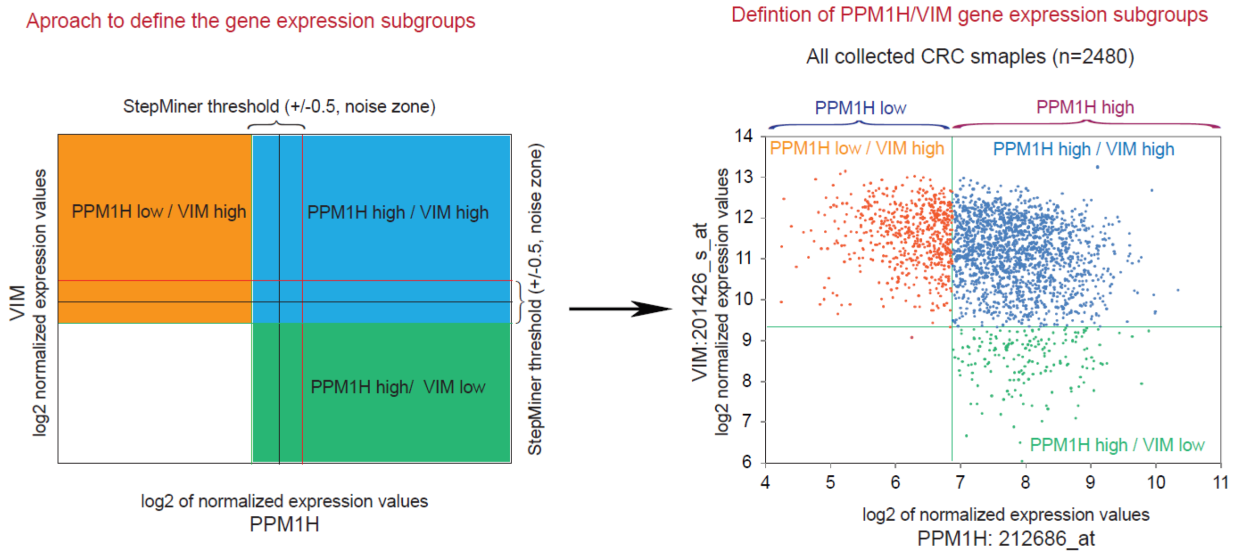

Supplementary Figure 4. **The classification of 3 subgroups based on the expression pattern of PPM1H and VIM.**

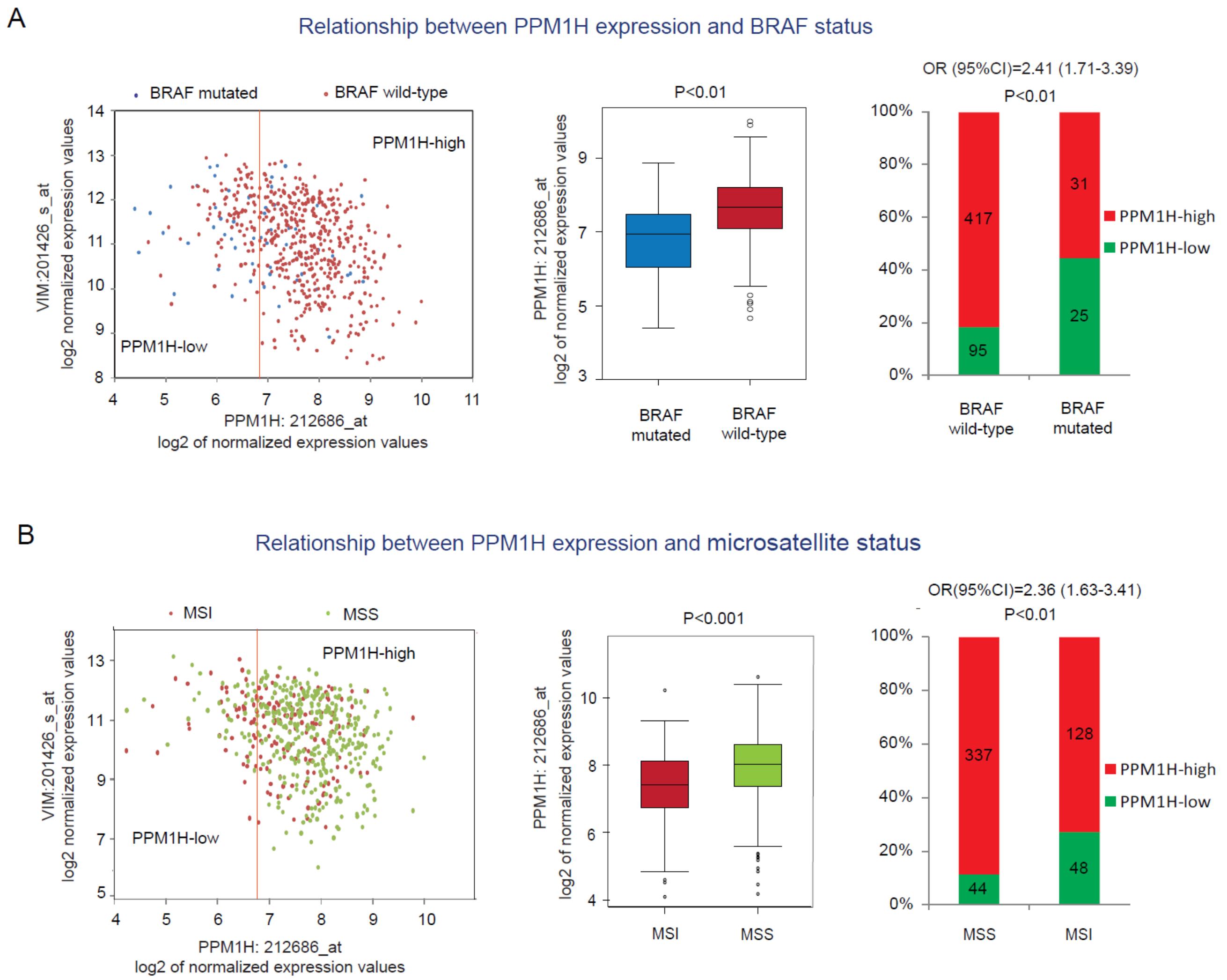

Supplementary Figure 5. **Relationships between PPM1H mRNA expression and BRAF mutations (A) or microsatellite status (MSI) (B).** The cut-value of PPM1H mRNA expression was identified by the StepMiner approach.

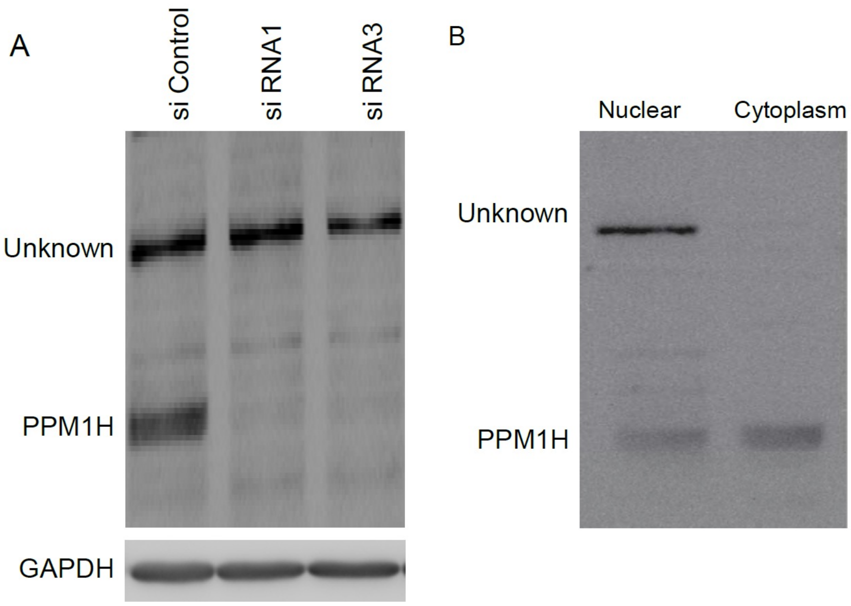

Supplementary Figure 6. **Evaluation the specificity of anti-PPM1H used in this study with Western Blot examination.** (A) anti-PPM1H (SAB1301160, Sigma-Aldrich, USA) used in this study showed 2 bands and the lower one is specific to PPM1H siRNA duplexes interference; (B) cytoplasm PPM1H could be detected with the anti-PPM1H used in this study characteristically.

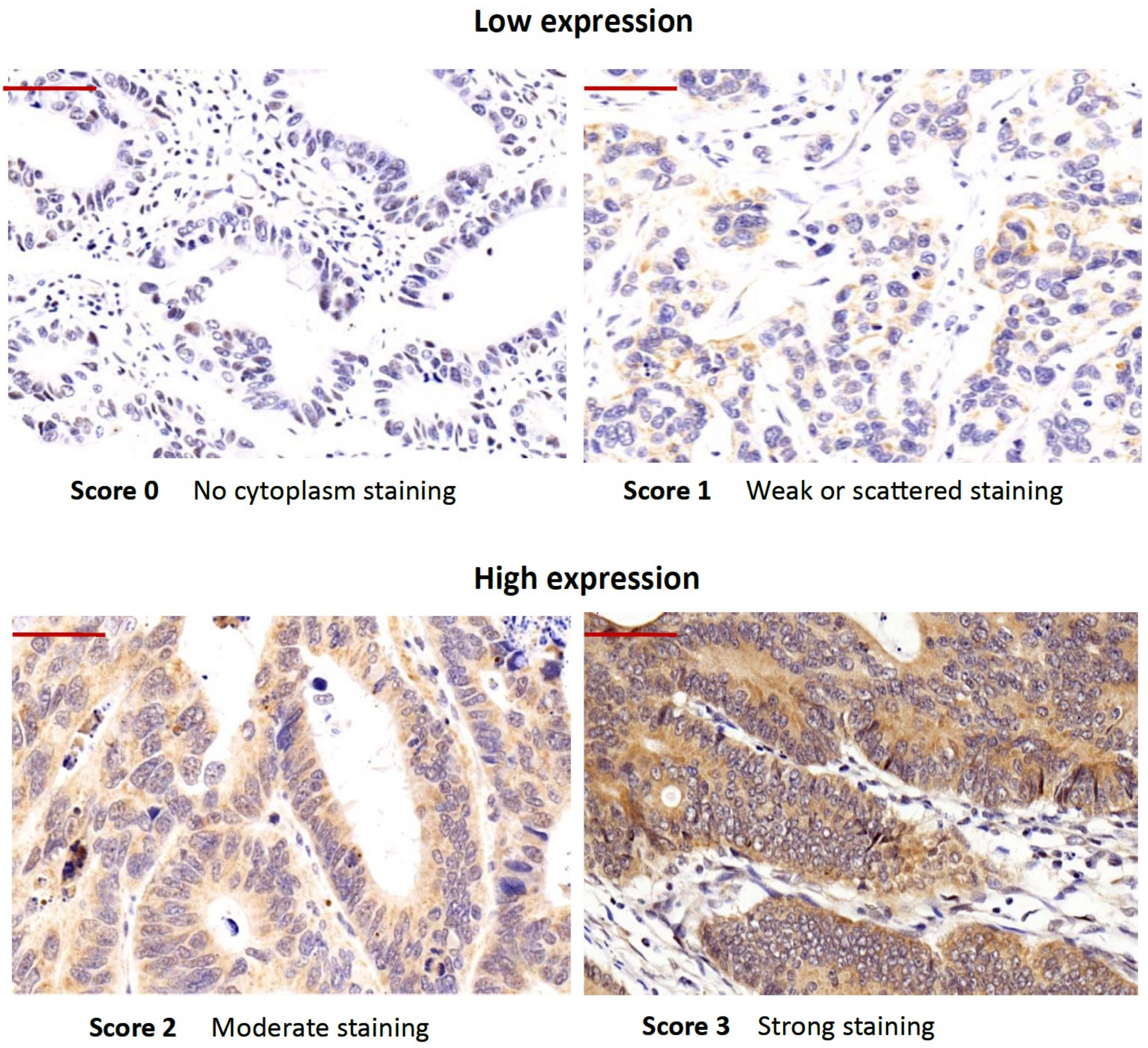

Supplementary Figure 7. **Immunohistochemistry scoring system for PPM1H protein expression in colorectal specimens.**

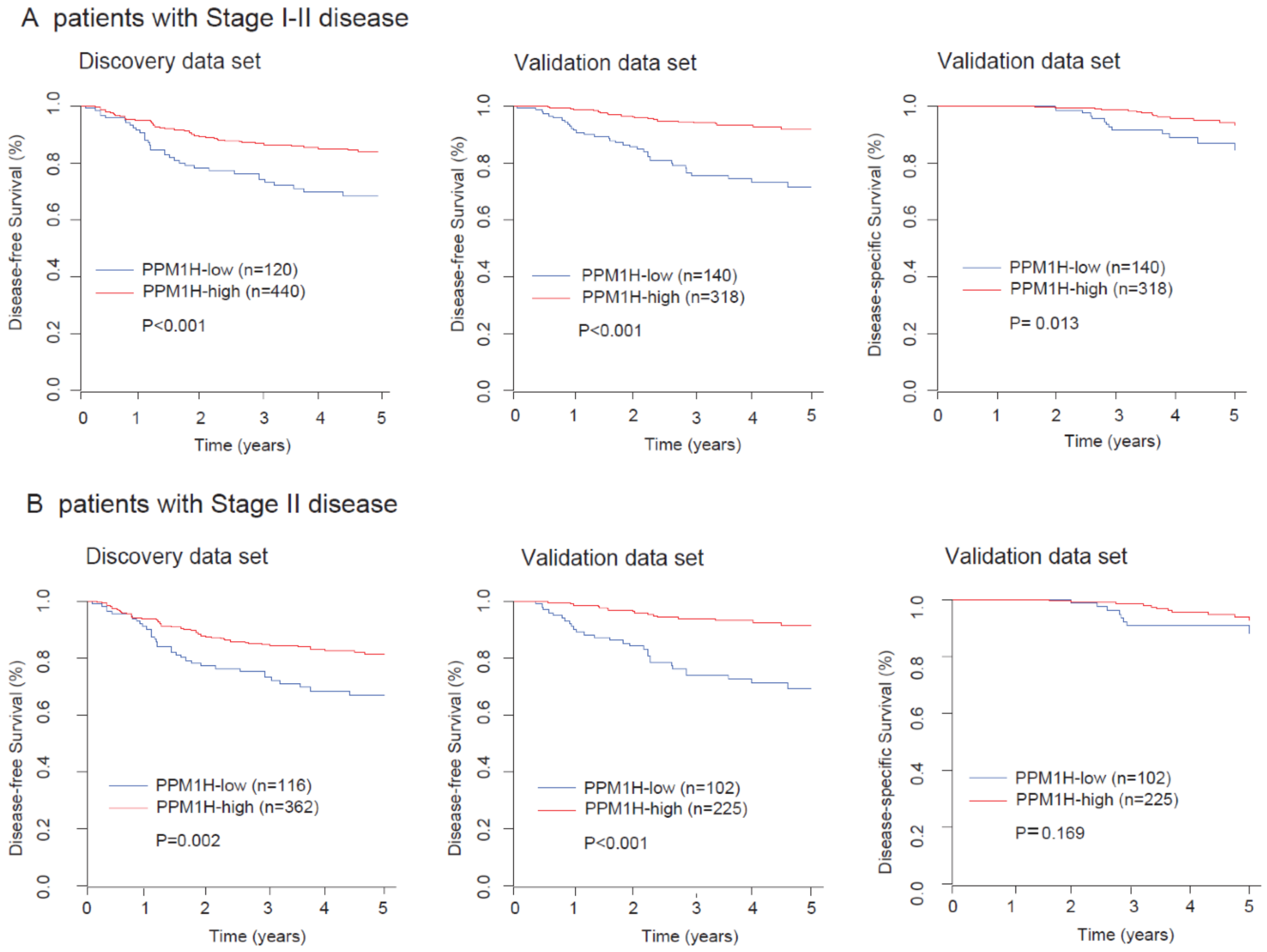

Supplementary Figure 8. **Patients with PPM1H-low tumors had poor survival outcomes at early stage CRC.** (A) PPM1H expression and patients' survival outcomes for CRC patients with stage I or II tumor; (B) PPM1H expression and patients' survival outcomes for CRC patients with stage II tumor.

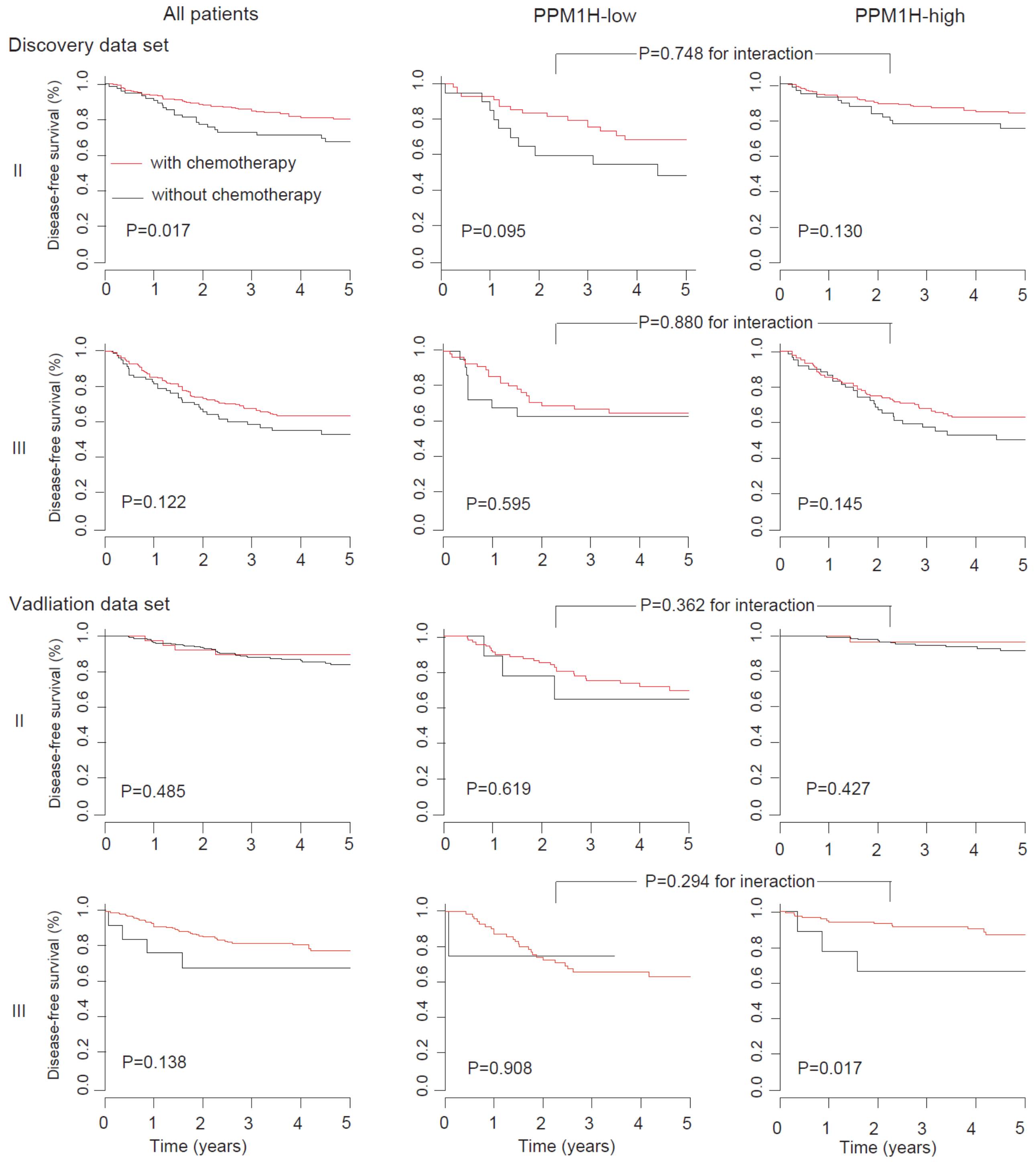

Supplementary Figure 9. **Relationship between PPM1H expression and patients' benefit from adjuvant chemotherapy.**

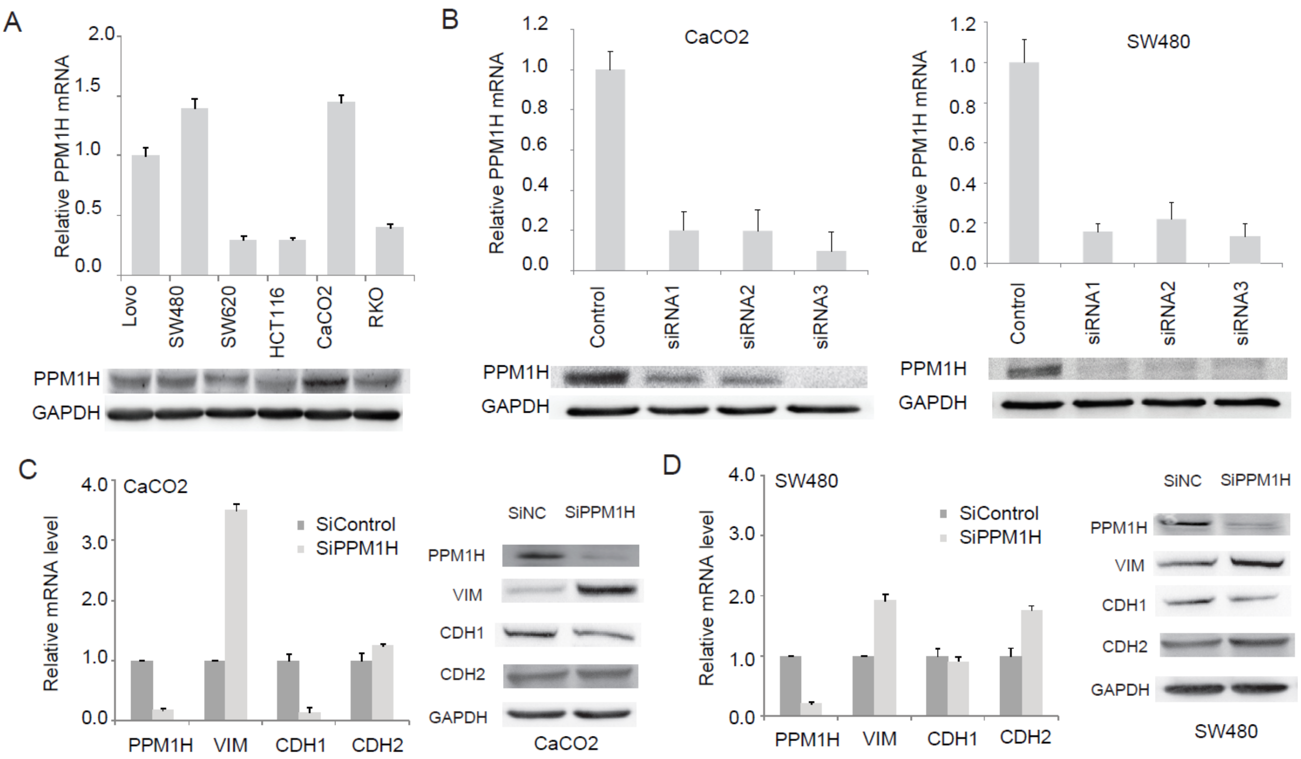

Supplementary Figure 10. PPM1H knockdown elevated the expression of VIM in the cells of colorectal cancer**.** (A) The baseline expression of PPM1H in the involved CRC cells examined by qPCR (upper panel) and Western Blot (lower panel); (B) The efficiencies of PPM1H siRNA pairs 1-3 reducing PPM1H expression in the indicated cells detected by qPCR (upper panel) and Western Blot (lower panel); (C, D) The effect of PPM1H on the EMT markers examined by qPCR (upper panel) and Western Blot (lower panel).

A

PPM1H score of individual tissue cores		Observer #1			
		Score 0	Score 1	Score 2	Score 3
Observer #2	Score 0	61	7	0	0
	Score 1	15	145	2	0
	Score 2	0	1	337	35
	Score 3	0	0	10	78

Kappa with linear weighting = 0.885, 95%CI=0.859-0.910

B

PPM1H score of individual tissue cores		Observer #1	
		PPM1H-neg	PPM1H-pos
Observer #2	PPM1H-neg	228	2
	PPM1H-pos	1	460

Kappa with linear weighting = 0.990, 95%CI=0.979-1.000

Supplementary Figure 11. **Inter-observer agreement between 2 observers in the scoring of PPM1H immunostaining.** The Cohen's Kappa Index was calculated for the concordance analysis between the two observers. The result is considered an excellent concordance when the Cohen's Kappa Index more than 0.8. The agreements showed quite excellent both in terms of cytoplasm PPM1H staining scoring of both the individual scores (A) and the final scores (B) for the individual patients.

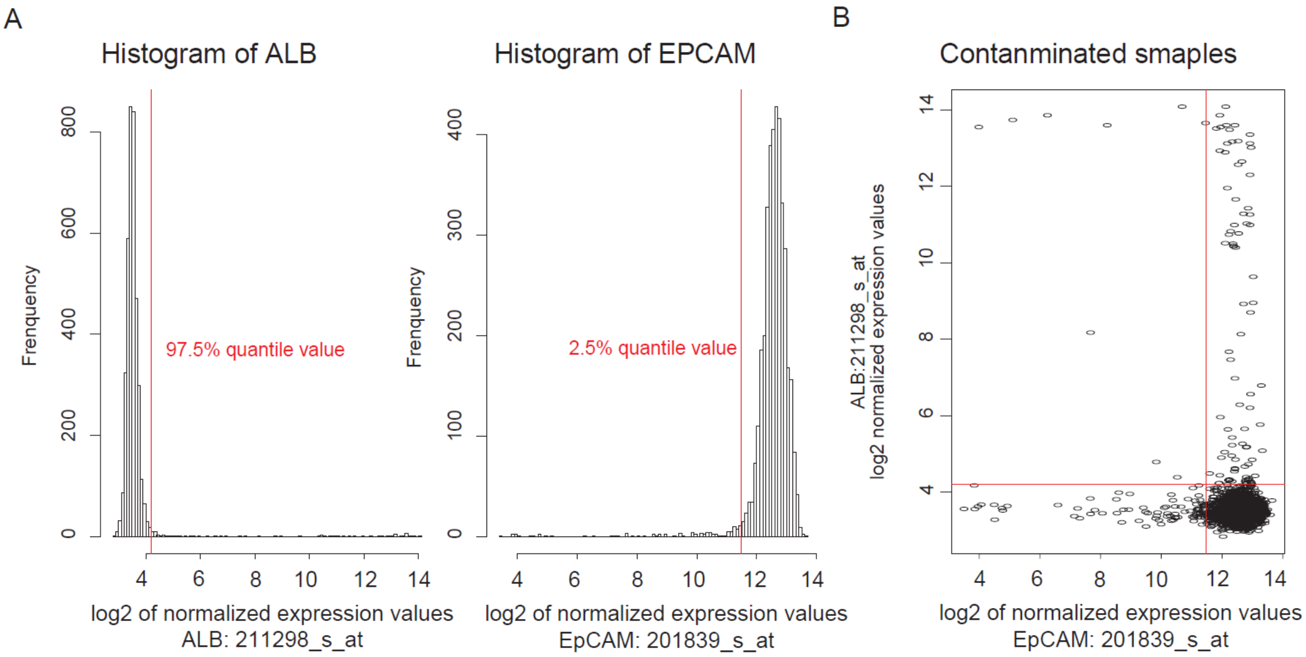

Supplementary Figure 12. **Exclusion of the samples which might be affected by poor quality.** (A) Identification of the cut-points of ALB (a positive marker for the presence of hepatocytes contamination) and EpCAM (a positive marker for the presence of colon epithelial cells). (B) The included samples were located in the lower right quadrant.
